# The mind & muscles: Introducing a validated EEG/EMG protocol for recording cognitive-muscular interactions in experimental archaeology

**DOI:** 10.1371/journal.pone.0324103

**Published:** 2025-05-23

**Authors:** Brienna Eteson, Simona Affinito, Fotios Alexandros Karakostis

**Affiliations:** 1 DFG Center for Advanced Studies “Words, Bones, Genes, Tools”, Department of Geosciences, Eberhard Karls University of Tübingen, Tübingen, Germany; 2 Paleoanthropology, Senckenberg Centre for Human Evolution and Palaeoenvironment, Department of Geosciences, Eberhard Karls University of Tübingen, Tübingen, Germany; 3 Integrative Prehistory and Archaeological Science, University of Basel, Basel, Switzerland; Tehran University of Medical Sciences, ISLAMIC REPUBLIC OF IRAN

## Abstract

Despite extensive research into the biomechanical and cognitive dimensions of early hominin material culture, no study has explored these aspects together in the context of stone tool production and use. In contrast to fields like rehabilitation and sports science, where electroencephalography (EEG) and surface electromyography (sEMG) are often integrated, experimental archaeology lacks such a combined approach. This paper introduces and validates a new protocol that integrates EEG and sEMG to measure neuromechanical activity during a classic stone tool task: cutting leather with a flake. Our experimental design divides the task into three phases: Hold, Aim, and Execute. Consistent with our expectations, results show that all eight muscles are most active during task execution, with the non-dominant hand playing a key role in stabilization during both the Aim and Execute phases. In the preparatory Aim stage, we observed increased beta power in the left frontal region (linked to planning, problem-solving, and working memory) as well as heightened motor activity associated with using the non-dominant hand, which contributes to the stabilization of the target material during this stage. During the Execute phase, beta power in these cortical areas decreased, with peak muscle activation occurring alongside suspected beta desynchronization in the motor region, reflecting intensified movement activity. Overall, these findings closely align with our expectations, validating our combined EEG-sEMG protocol and highlighting the importance of segmenting tool-using tasks into distinct phases, which allows for the identification of dynamic brain-hand interactions throughout the process. The proposed step-by-step protocol offers a new methodological basis for future research into the complexities of hominin behaviors and tool use.

## Introduction

Elucidating the physiological and cognitive implications of hominin tool-using behaviors represents one of the main goals of evolutionary sciences. Many studies have previously reported on the various requirements to perform stone tool tasks (i.e., knapping [1–7], cutting [8–11], and pounding activities [1,11–14]). However, there is yet to be a study that combines the use of multiple methodologies within the same analytical framework, to simultaneously assess both the biomechanical and cognitive requirements of stone tool production and use. More broadly, there appears to be a significant scarcity of detailed step-by-step experimental protocols in the archaeological sciences. This gap often results in a lack of methodological consistency across studies that use similar recording methods, making it difficult for new researchers to learn, replicate, and advance work in this important field.

The functional demands necessary for early hominin stone tool production and use have been a major focus of archaeological sciences for over 20 years [6,8,10,15]. In fact, experimental archaeology as a field has grown in response to an increasing range of archeological material being discovered, including proposed stone tools dating from ~3 MYA [16,17]. Tool use is not only undoubtedly essential to human life, but also a crucial part of many other primate societies [18–20]. This behavior, previously thought to be unique to humans [21], has continually intrigued anthropologists, leading to many experimental studies exploring the foundations of early stone tool production and use, from focus on the biomechanical requirements and grasping patterns required to wield such tools [6,8,10,15,22], to the cognitive activation necessary to perform these tasks [3–5,7,23]. However, despite the various modes of tool use witnessed within the primate order, there is a specific early stone tool task that seems exclusive among hominins (at least in the wild): stone flake-cutting. The earliest use of cutting is reported to date back to ~3 MYA, reflected in the presence of archaeological material such as cut marks on bone [17,24,25] and the emergence of intentionally produced Oldowan flakes [17]. The Oldowan succeeds the proposed Lomekwian industry (dated ~3.3 MYA [16]) and is often referred to as the oldest persistent stone tool industry, both temporally and spatially [17]. Given the critical importance of this tool task for exploring the origins of hominin behavior and considering the need to develop protocols that address both the cognitive and biomechanical demands of stone tool use, this paper aims to introduce a new combined methodology using surface electromyography (sEMG) and electroencephalography (EEG) at the same time, to simultaneously analyze both muscular and cognitive activity during the widely studied Oldowan flake-cutting task.

Previous sEMG studies on the biomechanical requirements of early stone tool use have provided key insights into the grasping patterns and necessary muscular activation [6,8–10,15] to perform such tasks. Additionally, multiple studies have found certain grips are repeatedly recruited during stone tool tasks [9,26–30], which have laid the groundwork for understanding some of the key muscles, grips, and digits most often recruited during lithic production and use. Additionally, over the past 20 years, several studies have worked on another physiological aspect of stone tool experience, by analyzing the cognitive activity in modern humans during and after performing various tasks, such as knapping stone tools [3–5,23]. These various works have yielded particularly insightful results. By analyzing different tool technologies these studies have explored the underlying cognitive processes and activation of brain regions during these tasks [3–5,7,23]. Additionally, some studies have attested that the areas of the brain activated after performing such tasks are linked to regions associated with communication and language processing [31]. However, despite these promising results, there is yet to be a study that analyzes both aspects, muscular and cognitive activation, within the evolutionary sciences. This leaves a major gap within this field, despite other fields of study, such as biomedical rehabilitation [32–34] and the sports sciences [35–38], regularly using this combined methodology within their research.

Previous neuroimaging techniques used in experimental archaeology, including fMRI, PET, and fNIRS [3–5,23], have been instrumental in advancing our understanding of the cognitive demands of stone tool production. These methods offer high spatial resolution, allowing researchers to pinpoint specific brain regions activated during tool-related tasks, thus establishing a foundation of brain areas relevant to these activities [39,40]. However, some of these techniques come with practical challenges, i.e. fMRI and PET have a low temporal resolution, require expensive, non-portable equipment, and often limit participant movement, making it difficult to observe the distinct, structured phases of human-like tool use in real-time [5,23]. Furthermore, the combined use of these neuroimaging methodologies with muscle-recording approaches (e.g., sEMG) within a single experimental design remains rare, resulting in gaps in capturing the dynamic interplay between cognitive and motor demands during actual tool use [3–5,23]. By analyzing the neuro-mechanical processes involved in tool use in real-time, we can gain deeper insights into the requirements for each stage within a tool-using task. For example, understanding the role of brain regions associated with the use of specific muscles for specific stone tool tasks can enhance our comprehension of the energetic demands (ergonomics) associated with each tool-producing or using activity [6,8,11]. Moreover, previous analyses of modern tool use (e.g., [41–44]) have provided valuable insights into the dynamic cognitive patterns required during specific phases or segmentation (such as a preparatory or planning stage and a preview stage).

To bridge the current gap between cognitive and biomechanical studies on stone tool use, we developed a new protocol for the combined use of EEG and sEMG. This protocol was already successfully used in two recent studies that focused on the results of each technique separately [11,45]. These recording methods were chosen for their high temporal resolution (millisecond-level), which allows for accurate, simultaneous recordings of both EEG and sEMG signals [32–38]. Their portability also enables recording during active movement. Drawing on successful applications in other fields [32–38], we developed a protocol that ensures appropriate preprocessing (including cleaning), and analysis of the data resulting from the two combined methods utilized here. In this study, we validate the use of this integrative protocol by focusing on the widely-studied task of Oldowan flake-cutting, which is divided here into three distinct phases (also see [11,45]); the first stage, Hold, involves picking up the tool with the dominant hand; the second stage, Aim, entails positioning the tool at the target object (faux leather) in preparation, whilst stabilizing the fabric using the non-dominant hand; and the final stage, Execute, focuses on cutting through the faux leather using the flake (which is still stabilized by the non-dominant hand). These separate phases allow us to explore the intricate demands of each part of the task.

Focusing on this data, we predict that muscular activation will steadily increase throughout the task in all muscles recorded, with a peak activation occurring in the Execute stage. This is due to an expected increased ergonomic demand during cutting, due to sustained pressure, necessary manipulation of the tool cutting edge, and resistance applied against the cutting object (i.e., hide) [9,10,26,46,47], which requires greater force and dexterity than the previous phases. We also expect this pattern to be reflected in the motor cortex, with increased activation occurring during the Execute stage. Cognitive tool use studies typically demonstrate engagement in specialized regions within the frontal, temporal, and parietal cortices during preparation and execution, due to their role in problem-solving, action planning, understanding of tool functions, sequenced motor skills, and planning of goal-directed movements [48–55]. Previous cognitive studies on stone tool production have demonstrated increased activity in the dorsolateral prefrontal cortex (PFC), and higher-level executive functions are associated with the ventral prefrontal cortex [3,23,31,56,57], whilst the left inferior parietal lobe (IPL) is crucially involved in understanding tool function, mechanical knowledge, and problem-solving [52,53,55]. Additionally, the temporal cortex is known to be critical for tool-related tasks, as it integrates sensory and semantic information [58,59]. While all these regions play a vital role in the planning and execution of tool use tasks, this protocol validation paper will focus its analyses on the frontal region, to demonstrate the utility of the combined EEG/EMG methodology outlined below. This was decided based on the inclusion of EMG, which enables direct links with the motor cortex, as well as the consideration of several other EEG studies reporting increased prefrontal activation during the preparational phases for voluntary motor tasks (including generalized tool use [48–51]). Additionally, the widely discussed role of increased PFC activity as a crucial aspect of modern human brain reorganization further justifies an explicit focus on this brain region [23,60]. More particularly, in this study, we primarily expect increased activation in the frontal (FC) and prefrontal (PFC) cortex during the Aim stage, as we expect participants at this point to be planning the necessary sequence of movements (i.e., motor planning), deciding in the order in which to make the necessary cuts within the time limit, and eventually initiating fine-motor control actions.

## Materials and methods

The protocol described in this peer-reviewed article is published on protocols.io (dx.doi.org/10.17504/protocols.io.36wgqnxbygk5/v1 [61]) and is included for printing purposes as S1 File. To validate this protocol, we conducted a re-analysis of data from two recent studies of ours that employed this protocol and yielded meaningful results on sEMG [11] and EEG [45] separately. However, the present lab protocol paper is the first to present a combined EEG and sEMG study on the same task concurrently, demonstrating the unique advantages of this integrative approach.

In summary, as described in two previous studies [11,45], we recruited twenty-five volunteer participants across two recruitment periods (May 1–31, 2022, and January 10–April 30, 2023), in accordance with the guidelines of Tübingen University’s Ethics Committee for Psychological Research (in line with the Declaration of Helsinki, 1964, revised in 2013), which have approved the experiments presented in this study. All individuals were adults and provided informed written consent. Using the Edinburgh Handedness Inventory [62], two participants were identified as being left-handed, and these participants were removed from the study to create a homogeneous sample, due to the known relationship between handedness and brain lateralization [63,64]. Therefore, the final number of participants was 23 (14 biological females and 9 biological males), between the ages of 22–55. All participants declared no physical injury that would prevent natural tool use and no history of neurological disorders. Stone tools were knapped (*n* = 26 Oldowan-style flakes) by an expert tool knapper (ETM; see Acknowledgments) in accordance with documented proportions [65–67]. A synthetic faux leather material was used as the object to cut, in place of hide or leather [15,68], to avoid potential variance in consistency (i.e., thickness, torque, strength). The fabric was also marked by three stenciled 3 cm straight lines forming a Z pattern to ensure consistency across all participants. During the processing stages of the recorded EEG and sEMG data, they were subjected to scrupulous cleaning and processing (see the detailed protocol and our previous research [11,45] for more), to ensure the data displayed true muscular and brain signals while reducing noise as much as possible [69–72]. However, in tool-using experiments involving EEG, the presence of artifacts in some channels is often inevitable [71,73–75] (for more information, see the Discussion). These may consist of internal artifacts; ocular (blinks and side eye movements), motion/muscular (excessive movement of the body or tension in the neck), and external noise (electrical equipment, electrode pops, cable movement, or bad channel connection) [71]. There is yet to be a processing technique that guarantees the complete removal of high frequency artifacts, particularly in tasks requiring movement [72], that ensures the real EEG signal is not lost [76,77].

### EEG and SEMG channel selection

The EEG aspect of the experiment relied on a 32-channel EEG cap, following the 10–20 system configuration, using Brain Vision Recorder software (version 1.24.0101, Brain Products GbmH, Gilching, Germany) [78]. The areas of particular interest in this paper are, the motor area, due to the nature of the task, requiring precisely executed voluntary movement; and the frontal region due to its previously researched importance in motor tasks and association in regards to planning, problem-solving, and working memory [3,48–51,79]. Muscle selection was guided by previous stone tool experiments and studies on hominin skeletal remains, highlighting the established importance of specific muscles for achieving key grasping patterns during stone tool use (in this case, cutting) [8,15,80–82]. These muscles are also crucial for the stabilization of the target object provided by the non-dominant hand and for the force exerted in manual tool engagement [6,8,15,28,30,80,83,84]. Therefore, the following muscles and muscle groups were selected for sEMG recording from the dominant hand; *flexor carpi radialis* (FCR), *flexor carpi ulnaris* (FCU), *flexor pollicis longus* (FPL), the thenar eminence (TE) (consisting of *abductor pollicis brevis*, *flexor pollicis brevis*, and *opponens pollicis*), first dorsal *interosseus* (DI1), and the hypothenar eminence (HTE) (consisting of *abductor digiti minimi*, *flexor digiti minimi*, and *opponens digit minimi*), and two muscles/muscle groups from the non-dominant hand; first dorsal *interosseus* (ndDI1) and the thenar eminence (ndTE). All anatomical placements were based on descriptions provided in standard anatomical textbooks [83]. Some muscles were grouped because the overlaying skin (onto which the electrode attached) corresponded to more than one muscle underneath (i.e., the thenar and the hypothenar eminence muscles; (for more, see [11]). For a detailed breakdown of the forearm and hand muscles selected for sEMG recording and analysis, and the channels and configuration used in EEG recordings, see dx.doi.org/10.17504/protocols.io.36wgqnxbygk5/v1 [61] and [45]. Once cleaned and exported, all sEMG muscular signals were transformed into a percentage maximum voluntary contraction (%MVC) [8,11,85,86]. These allowed participants to be directly comparable with one another despite interindividual differences in body size and muscle strength [8,11,85,86]. For a detailed breakdown of the MVC-obtaining process, see dx.doi.org/10.17504/protocols.io.36wgqnxbygk5/v1 [61].

### Experimental design

The experiment took place in a shielded cabin at the Max Planck Institute for Intelligent Systems in Tübingen (see Acknowledgements). Participants first watched an instructional video detailing the experimental design, EEG and sEMG application, and experiment contraindications (see dx.doi.org/10.17504/protocols.io.36wgqnxbygk5/v1 [61]). Each participant completed an Oldowan-style flake-cutting task, consisting of three phases: picking up the tool (Hold), aiming the tool at the target while stabilizing the faux leather (Aim), and cutting through and stabilizing the faux leather (Execute). Participants were informed that their trials would be assessed for success and were instructed that a successful trial entailed cutting through the fibers of the faux leather entirely (following the indicated Z-pattern), within the provided period of 5 seconds. Participants were not instructed to cut through the marked lines on the fabric in a specific order or to hold the tool in any specific grasp. Therefore, they needed to employ a certain degree of planning and working memory prior to executing the cutting sequence, at the Aim stage. A looped “beep” stimulus is played every 5 seconds to signal and mark the start of each stage (Hold, Aim, Execute). Based on recommendations for EEG signal-to-noise optimization, each participant completed a minimum of 50 trials per task to ensure robust data quality (e.g., see [87–90]). In addition to the stone tool task, a control task was performed. The latter involved a simple motor action (opening and closing the hand) without the use of a tool, which was expected to primarily involve distinctive activation in the motor region. Comparing tool-using tasks and the control condition is crucial because it allows us to distinguish between generic hand use and stone tool usage per se [90]. The European standards for sEMG analyses (SENIAM project) were followed [91]. For a more detailed breakdown of the recording and processing of EEG and sEMG data, see dx.doi.org/10.17504/protocols.io.36wgqnxbygk5/v1 [61], Eteson and colleagues [11] and Affinito and colleagues [45].

### Data visualization and analyses

To analyze the EEG and sEMG data, several visualization techniques and statistical tests were performed. Data visualization was performed in BrainVision Analyzer (version 2.2.1, Brain Products GbmH, Gilching, Germany) [72] and the software package PAST version 4.03. [92]. All statistical analyses were performed in the open-access software PAST version 4.03. [92]. Plots and band maps were produced in the same software [72,92] and modified in Inkscape vector graphics editor version 1.3. [93].

To investigate sEMG muscle synergies, a principal component analysis (PCA) was performed, using all eight muscle recordings (%MVCs) as variables and including all three phases of the tool-using process (Hold, Aim, and Execute) [11]. This approach was chosen to efficiently analyze the interaction among different muscles within the same analytical framework and therefore address our expectation that muscle co-recruitment will gradually increase between the phases of Hold, Aim, and Execute. An additional PCA was performed excluding the two non-dominant hand muscles/muscle groups (ndDI1 and ndTE), to ensure results were not skewed, as only the Aim and Execute phases recruited the non-dominant muscles during the task (for stabilization of the faux leather). The PCAs were performed based on a correlation matrix (Table 1 and S2 Table). The PCAs did not assume groups *a priori*, and the three phases were simply color-labeled to differentiate between them. Statistical comparisons were then performed on relevant principal component (PC) scores, with the broken-stick model used to determine the number of PCs to extract [94]. In particular, prior to further statistical analysis, a Shapiro-Wilk normality test was run on the PC scores of each axis, to ensure that the extracted values followed an approximately normal distribution. Additionally, outliers were detected using the interquartile range approach [95]. Subsequently, repeated-measures ANOVAs were performed on the extracted PC scores of the task phases (Hold, Aim, and Execute), to assess if the muscular activation significantly varies across phases [8,11,95,96], effectively addressing our predictions outlined in the Introduction. Furthermore, Tukey’s post hoc pairwise tests were run to determine which phases exactly differ between them. In addition, to visualize the differences in muscular activation across the three phases within each participant, stem graphs were generated.

**Table 1:** List of eigenvalues, percentages of variance, and factor loadings for the first two principal components of the PCA.

				Factor loadings							
Task	Principal Component	Eigenvalue	% of variance	DI1	HTE	FCR	FCU	FPL	TE	ndDI1	ndTE
**Flake-Cutting**	PC 1	5.14	64.29	0.39	0.33	0.37	0.39	0.38	0.32	0.32	0.32
	PC 2	0.75	9.39	−0.09	0.42	−0.23	−0.26	−0.45	0.56	0.40	−0.16

Muscle abbreviations are as follows: FCR, *flexor carpi radialis*; FCU, *flexor carpi ulnari*s; FPL, *flexor pollicis longus*; TE, thenar eminence (consisting of *abductor pollicis brevis*, *flexor pollicis brevis*, and *opponens pollicis*); DI1, first dorsal *interosseus*; HTE, hypothenar eminence (consisting of *abductor digiti minimi*, *flexor digiti minimi*, and *opponens digit minimi*)*; ndDI1,* non-dominant first dorsal *interosseus*; ndTE, non-dominant thenar eminence.

In this lab protocol validation study, to visualize the EEG data recorded in the same individuals in tandem with sEMG, standard analytical processes were followed. A Fast Fourier Transformation (FFT) was applied with a 10% Hanning window, to compute the power spectrum of the segmented EEG data in the Brain Vision Analyzer (version 2.2.1) software [72]. All trials were then averaged to create a mean power spectrum for each participant. For a detailed breakdown of all pre-processing steps, see dx.doi.org/10.17504/protocols.io.36wgqnxbygk5/v1 [61] and Affinito and colleagues [45]. Grand average FFT band maps were exported from Brain Vision Analyzer (version 2.2.1) [72] in the relevant frequency bands. This study focuses on the beta (12.5–30 Hz) [97] frequency band, due to its known association with increased alertness during task performance [36,98–102]. All band maps were scaled between 0.1 µV² and 0.6 µV². These band maps visually depict the squared amplitude of EEG in the beta frequency during each stage of the task as well as the control task. They are used to compare levels of beta power in the frontal and central regions of the brain, which have been associated with fine motor control, planning, decision-making, and working memory [3,48–51,79]. Finally, band maps were then imported into Inkscape [93], alongside the sEMG PCA plot, to facilitate the visualization of the two integrated methodologies jointly. At the same time, individual phases were initially compared against the control task, allowing us to determine common brain region activation during both the stone flake-cutting task and the control task.

### Expected results

All plots, graphs, and tables mentioned in Materials and Methods that do not appear in the results section are available in the linked protocol (see dx.doi.org/10.17504/protocols.io.36wgqnxbygk5/v1 [61]) or in the supporting information. For all statistical tests, an alpha level of 0.05 was used. According to the Shapiro-Wilk tests, all %MVC values and PC scores showed an approximately normal distribution (*p* > 0.05). Moreover, no extreme outliers were detected. Therefore, parametric tests were conducted when analyzing the sEMG data.

### Predictions of muscle use (sEMG)

**Fig 1:**
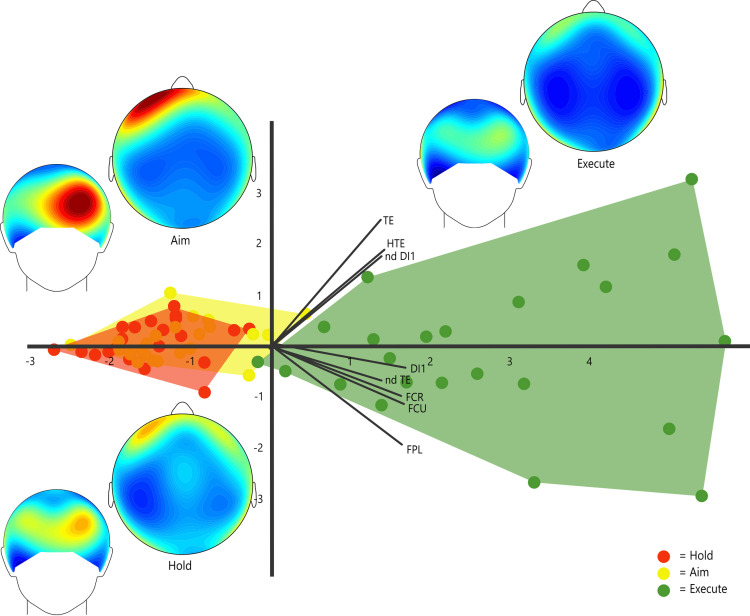
%MVC sEMG PCA plot (PC 1 **= 64.29% of variance; PC 2 = 9.39% of variance).** PCA plot summarizing overall muscular recruitment, with accompanying EEG bands maps (beta) displaying beta power levels throughout the cutting task (i.e., during Hold, Aim, and Execute). Phases are color-labeled (Red = Hold; Yellow = Aim; Green = Execute). PC 1 (64.29% of variance) reveals a clear contrast between Execute and the other phases of the task (Hold and Aim), with the former displaying increased activation across muscles (factor loadings are listed in Table 1).

**Fig 2:**
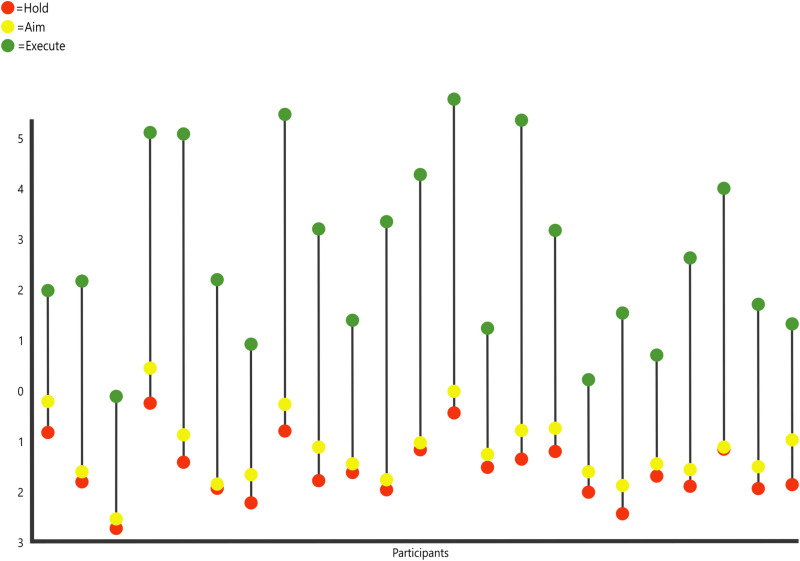
Stem plot of PC 1 scores (vertical axis), representing all three phases within participants Hold (red); Aim (yellow); Execute (green). Each stem represents a participant. All participants’ PC1 scores for the Execute stage were larger than their Hold and Aim PC1 scores.

To observe variation in relative muscle recruitment across the three phases of the cutting task, a PCA was performed on sEMG data. PC 1 (64.29% of variance) showed most of the variance explained, with all variables (muscles) loading positively, indicating that variation on PC1 reflects overall muscle recruitment (across variables; also see the Discussion subsection below for further interpretations of the observed patterns). Following the indication of the broken-stick technique, only the first principal component (PC 1) was extracted. The PCA plot clearly distinguishes between the phases, with flake Execute showing increased activation in all muscles (both dominant and non-dominant hand) in all participants, compared to Hold and Aim (see Table 1, Figs 1 and 2), and the Hold stage demonstrating the lowest overall muscular activation, as hypothesized. Descriptive statistics (Table 2) further supported the results shown in the %MVC sEMG PCA plot (Fig 1). The relevant PC1 scores were then analyzed using repeated measures ANOVA, which displayed significant differences across phases (df = 2 *F* = 196.2, *p* < 0.001). Furthermore, a Tukey’s Pairwise test indicated that both Hold (*p* < 0.001) and Aim (*p* < 0.001) are significantly different from Execute, however, Hold and Aim are not significantly different from each other (*p* = 0.24). A stem graph (Fig 2) displays the variation in muscular activation across PC 1 scores of each stage. This stem graph further confirms our prediction that the Execute stage involves significantly higher muscular activation in all eight muscles, compared to both the Hold and Aim phases. Muscular activation is also marginally higher in all participants during the Aim stage, when compared to Hold, as predicted. Results remained significant (d.f. = 2, *F* = 80.52, *p* < 0.001) when the non-dominant hand muscles were removed from the analysis (see S3 Fig. for PCA plot). Tukey’s pairwise tests again confirmed that significance was maintained between the Execute phase and both Hold (*p* < 0.001) and Aim (*p* < 0.001), even when the non-dominant hand variables were excluded (see S2 Table for PCA statistics).

**Table 2:** Descriptive statistics of the mean %MVC values for each stage.

Task	Stage	DI1	HTE	FCR	FCU	FPL	TE	ndDI1	ndTE
**Flake**	**Hold**	1.61	2.97	3.19	1.62	2.43	4.19	0.54	0.96
	**Aim**	1.95	2.72	2.82	1.81	2.39	3.38	2.87	8.16
	**Execute**	11.80	6.66	7.11	7.50	9.86	13.52	6.15	17.45

For each muscle, the highest mean value is highlighted in green, and the second highest value is highlighted in amber. Overall, a comparison between phases within the tool-using task shows that the Execute stage consistently recruits more muscular activity across all eight muscles than either Hold or Aim.

### Predictions of brain activation (EEG)

**Fig. 3:**
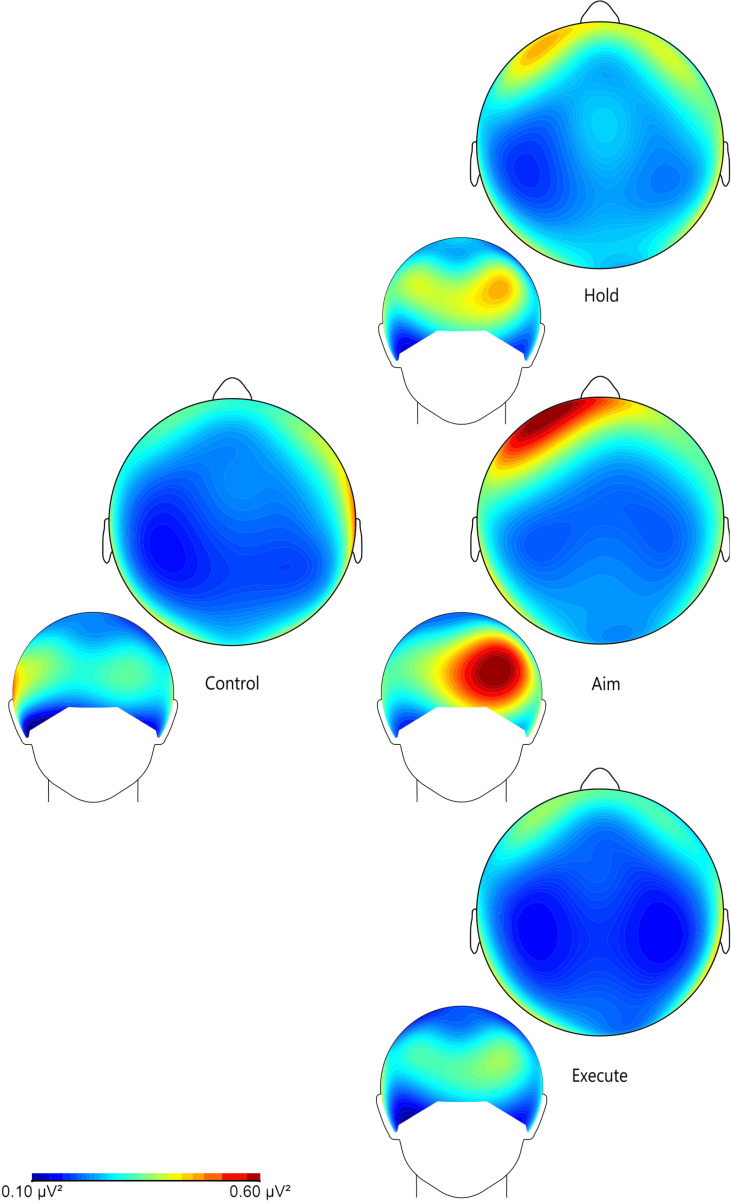
Beta frequency band maps of all three phases within the Oldowan flake-cutting task and the control task Maps are configured at 0.1–0.6 µV². The scale represents lowest to highest power, from blue to red respectively. Band maps shown are from front and top views, focusing on the brain areas of interest.

Initially, all phases’ band maps were compared against the control task (Fig 3). In all cases, beta power displays increased levels across the frontal brain region, when compared to the control task, as expected. Nevertheless, increased power in the frontal region displays minimal differences between the control task and the Execute stage. One reasonable explanation for this may be that the necessary planning process occurs prior to this final stage of the task, during the preparatory phases Hold and Aim (see Discussion for more in-depth detail).

A total of six frequency band maps (two for each stage, focusing on the areas of interest; see Materials and Methods) were produced alongside the muscle recruitment EMG PCA plot (Fig 1). These band maps were comparatively examined to evaluate the expectations of this analysis regarding neural activation patterns in the frontal lobe (for further statistical analyses and results, see Affinito et al. [45]). Based on these (Fig 3), both Hold and Aim can be seen recruiting higher levels of beta power in the left frontal area when compared to the Execute stage. The strongest beta power is observed in the frontal lobe and occurs during the Aim stage, which shows a sharp increase in power (particularly in the left lateral frontal region). This finding clearly aligns with the study’s expectations (see Introduction) as the left PFC has been associated with increased focus on the processing of stimuli and fine-motor planning [3,48–51,79] during precise control of tools, including the processes of adjusting grip strength, positioning the hand/tool, and deciding the angle of the first cut. The Hold stage also seems to recruit increased levels of beta power in the frontal region, compared to the control task and the Execute stage.

In addition to the above patterns of higher beta power in the frontal region during Aim, which has been associated with an increased state of alertness [103–105], a phenomenon known as event-related desynchronization (ERD) is also known in event-related potential (ERP) studies to occur during voluntary movement [38,105–108] which, despite showing a visible decrease in beta power in sensorimotor brain areas, is linked to a state of motor activation [105,107]. This phenomenon is typically explicit to the sensorimotor areas, known to display a pronounced decrease in the beta frequency band [38]. As this study’s cutting task involves voluntary active movements, particularly including shifts from an idle state to an active processing state that involves decision-making, planning, and fine motor control, the ERD phenomenon is a likely explanation for some of our results, particularly in the Execute stage [38,105–109]. We argue that this is to be expected when analyzing such a continuous series of repetitive hand motions (i.e., the consequent cutting motions of the Execute stage and the repetitive finger movements of the control task). At the same time, as previously suggested in Affinito et al. [45], it is also possible that many of the cognitive processes essential for successful task performance occur primarily during the preparatory (Hold and Aim) phases of the overall activity (involving the frontal region), prior to actual “cutting”.

The findings also reveal intriguing patterns in bimanual coordination across different phases of the task. In the cutting task, participants used their non-dominant hand (in this case, the left hand) to stabilize the faux leather during both the preparation phase (Aim) and the cutting phase (Execute). Notably, this additional role of the non-dominant hand is reflected in the motor area (see Fig 3). Specifically, during the Hold phase (unimanual stage), only the left hemisphere of the motor region, which controls movements on the dominant (right) side of the body, displays low levels of beta power. This aligns with the expected motion-related desynchronization pattern observed in similar motor tasks [110,111]. However, in the Aim and Execute (bimanual phases) band maps, both the left and right hemispheres of the motor area show this relatively low level of beta power. Moreover, this is further visually confirmed by the control task band map (simple motor task), which predominantly shows low levels of beta power only in the left hemisphere of the motor region, as only the dominant hand was used.

## Discussion: Interpretations, limitations, and future possibilities

This paper aimed to introduce and evaluate a novel protocol for studying brain-hand interactions during stone tool use by simultaneously recording EEG and sEMG data during the well-studied task of flake-cutting [8,112–115]. We anticipated gradually increasing patterns of muscular activity across the three task phases: beginning with picking up the tool (Hold), followed by preparing and aiming it for action (Aim), and peaking in activation during the actual cutting of faux leather (Execute). Furthermore, we hypothesized that the Aim stage would show an increase in beta power in the PFC and FC, reflecting the task’s preparatory demands, which involve planning, object processing, and precise motor control [3,48–51,79]. Based on the interpretation of the produced beta frequency maps and the sEMG analyses, our core predictions were confirmed (also see previous studies on this data [11,45]), demonstrating that our proposed step-by-step protocol can provide a suitable basis for future experimental research on early stone tool production and use. Furthermore, this validation paper highlights the importance of distinguishing specific phases within each stone tool task during experiments. This segmentation allows for a more precise exploration of the unique biomechanical and cognitive demands required at each stage of the process [11,22,45].

More specifically, we found muscular forces increased during the Execute (cutting) stage, which showed distinctively higher positive PC1 scores. Notably, the muscles/muscle groups with the highest positive PC1 loadings (i.e., *flexor pollicis longus*, the two wrist flexors, and the first dorsal *interosseus*; Table 1) play a central role in performing the pad-to-side precision pinch grips typically required for flake-cutting [27,81,82,116]. Our analyses also successfully identified the increased importance of the non-dominant hand for stabilization of the target object, which was crucial not only during Execute (cutting) but also during the planning stage (Aim). This process (object stabilization) is used to ensure that the correct torque, resistance, and force are applied to the faux leather before pressure is exerted using the dominant hand carrying the flake.

Additionally, our EEG power band maps provided meaningful insights into the observed tool-related patterns of cognitive activation. As expected, these revealed a distinctive increase in beta power in the frontal region (particularly in association with the left PFC) during the Aim phase, compared to all other phases (including the control task). This aligns with findings from a recent human tool use study, which reported that the left frontal region (along with the left parietal region) was primarily activated during the planning phase of novel tool use, indicating that higher-order cognitive processes, such as goal-directed activities, are initiated prior to execution [44]. Other experimental tool use studies also find the frontal cortex critical in the planning and realization of precise motor planning [41,43]. In our study, in contrast to the Aim phase, Execute showed markedly less beta power in the PFC and FC. As we previously suggested in a recent EEG study [45], a likely explanation for this pattern is that the participants processed most of the motor planning, control, and decision-making required for stone tool use prior to the Execute phase onset, thus exhibiting increases in the frontal region during the earlier two phases of the task, but not during the cutting action itself. Alternatively, the decreased levels of beta power in the motor region during the Execute phase could also likely be indicative of increased motion-related brain activity (probably due to the aforementioned effects of desynchronization).

Nevertheless, it is worth noting that the above findings and interpretations regarding the Execute phase diverge from some of the studies mentioned above, which also reported continued frontoparietal involvement during the execution of the task itself [41,43]. In contrast, our analyses found diminished frontal activation once participants began the cutting action, suggesting that key cognitive processes, such as motor action planning, were largely resolved before movement onset (as previously proposed in Affinito et al., 2024 [45]) This discrepancy may stem from the relatively simpler nature of our stone tool task, as more complex or novel tool-using tasks may likely require sustained frontal involvement [41,44]. Another possibility, also proposed in our prior EEG study [45], is that continuous movement during the cutting task’s execution led to sustained beta ERD. Regardless, the observed differences between phases further highlight the importance of analyzing tool use across distinct phases (i.e., planning and execution) for acquiring a more complete understanding of the task’s cognitive demands. Studies and methods that focus solely on execution (e.g., cutting) or the entire task (without distinguishing between phases) risk overlooking critical neural processes involved in the structured process of goal-directed tool use (e.g., [8,15]).

This study’s central objective was to provide a clear validated example of the combined EEG/EMG methodology described in this lab protocol paper. For this purpose, this proof of concept focused on key brain regions (PFC and motor cortex) alongside relevant hand and forearm muscles. More extensive EEG-only data and results on this task have been already provided in a previous study of ours on the same data [45]. Nevertheless, in the context of this methodological work, it would also be relevant to concisely discuss the broader bases of tool use within the complementary fields of neuroscience and biomechanics. As mentioned in the Introduction, there are evidently also other brain regions and muscles involved in human tool use, not covered in this protocol’s case study. Prior research highlights the parietal lobe’s (particularly the left IPL) crucial involvement in tool-related conceptual (especially for understanding tool function, mechanical knowledge, and problem-solving) and motor planning [52,53,55]. The importance of the parietal region, alongside the frontal cortex, has been underscored by neurological lesion studies demonstrating that frontal damage alone may not necessarily cause major impairments, but when combined with parietal lesions, tool use deficits become more pronounced [52]. This broadly suggests a complementary relationship between planning and decision-making (largely occurring in the PFC) and the mechanical and spatial principles of tool use (largely occurring in the left parietal region). Our previous EEG study broadly confirmed this expectation, as increased engagement of the frontoparietal regions was observed during the structured Aim phase, which required participants to sequentially plan and execute three precise cuts in a Z-pattern [45]. In summary, our findings align with the established literature on the joint activity of the left-lateralized prefrontal and parietal regions during tool use. The PFC, particularly the inferior frontal gyrus, appears crucial for action planning, motor imagery, and executive control, while the parietal cortex underpins the practical application of tool knowledge, including mechanical problem-solving and the spatial organization of actions [3,23,31,48–57]. As for muscular recruitment, previous EMG studies have highlighted the importance of other upper arm muscles (biceps brachii, triceps brachii, anterior deltoid) and intrinsic hand muscles (palmar interossei, extensor digitorum) during stone tool use and especially Oldowan knapping tasks [6,8]. However, the muscle selection in this case study, as well as in our previous EMG research [11], relied on the knowledge that the highest activation during stone tool use occurs in the first, second, and fifth digits [6,8].

Given the complexity of this experimental protocol, which strives to address multiple parameters simultaneously, several inherent limitations should be noted. As with most controlled laboratory experiments, it is challenging to perfectly replicate fully natural conditions for task performance while adhering to methodological constraints and recommended ethical standards. Additionally, EEG and sEMG are highly prone to noise artifacts [71,73,76,77], especially in tasks involving extensive movement, such as the manual activities analyzed here (also discussed in [45]). Despite the rigorous recording strategy and processing steps followed to account for motion artifacts (see dx.doi.org/10.17504/protocols.io.36wgqnxbygk5/v1 [61] and [45]), certain channels are widely known to be susceptible to noise, i.e., the mastoids (TP9 and TP10), due to muscular tension, and the location of the carotid artery, causing pulse artifacts [72–75,117]. For this purpose, in addition to implementing a careful experimental design combined with a detailed artifact removal process (as outlined in our step-by-step protocol), we recommend excluding EEG channels from subsequent statistical comparisons that show consistent muscular artifacts (as in dx.doi.org/10.17504/protocols.io.36wgqnxbygk5/v1 [61]).

Additionally, although EEG and sEMG were chosen for their ability to capture cognitive and muscular activity synchronously (due to their high temporal resolution), it should be noted that EEG has limited spatial resolution compared to other neurological methods [118–121]. This can create challenges when the focus is on exact source localization (e.g., when targeting small important areas with major evolutionary implications, such as Broca’s or Wernicke’s areas). In this regard, future studies may greatly benefit from combining EEG with other neuroimaging techniques, such as fNIRS, with improved spatial resolution (as in [122–124]). Finally, the EEG data in this study was processed in the frequency domain, specifically the beta frequency. This is common practice in EEG research focusing on active movement tasks, including general tool use [99,125–130]. However, future studies may benefit from analyzing EEG activity in both alpha (8–12 Hz) and beta frequency bands, to enable a more comprehensive understanding. Analyzing the alpha frequency (and/or other bands) may provide a fuller perspective on cognitive activity during experimental tool use tasks, particularly since this frequency band is known to be active during relaxed wakefulness [131] and has also been linked to motor preparation and active movement [99,125,130,132].

Overall, this methodological study introduced the first combined approach for simultaneously recording and analyzing EEG and sEMG data in experimental archaeology, promoting the use of our step-by-step protocol for exploring brain-hand interactions during stone tool tasks (dx.doi.org/10.17504/protocols.io.36wgqnxbygk5/v1) [61]. We believe that this integrative method offers a currently missing foundation for gaining insights into the complex biomechanical and cognitive requirements of human-like stone tool production and use. Additionally, our studies highlight the importance of segmenting the stone tool task into distinct phases (Hold, Aim, Execute, in addition to a control condition), allowing for comprehensive monitoring of the entire process. This segmentation was shown to reveal valuable muscular and cognitive interactions across phases.

## Supporting information

S1 File Step-by-step protocol, also available on protocols.io (dx.doi.org/10.17504/protocols.io.36wgqnxbygk5/v1 [61]). (PDF)

S2 Table List of eigenvalues, percentages of variance, and factor loadings for the first principal components of the %MVC sEMG dominant hand PCA. Muscle abbreviations are as follows: FCR, *flexor carpi radialis*; FCU, *flexor carpi ulnaris*; FPL, *flexor pollicis longus*; TE, thenar eminence (consisting of *abductor pollicis brevis*, *flexor pollicis brevis*, and *opponens pollicis*); DI1, first dorsal *interosseus*; HTE, hypothenar eminence (consisting of *abductor digiti minimi*, *flexor digiti minimi*, and *opponens digit minimi*).
(PDF)

S3 Fig Dominant hand %MVC sEMG PCA plot (PC 1 = 70.27% of variance; and PC 2 = 11.59% of variance) PCA plot summarizing overall dominant hand muscular recruitment during all three phases of the flake-cutting task. Phases are color-labeled (Red = Hold; Yellow = Aim; Green = Execute). PC 1 (70.27% of variance) maintains a clear distinction between Execute and the other phases of the task (Hold and Aim). The Execute phase displays increased muscular activation across all dominant hand muscles (factor loadings are listed in S2 Table).
(TIF)
