# Supplementary material for: The mind & muscles: Introducing a validated EEG/EMG protocol for recording cognitive-muscular interactions in experimental archaeology
Source: PLoS One. 2025 May 23;20(5):e0324103. doi: 10.1371/journal.pone.0324103 (PMC12101640; doi:10.1371/journal.pone.0324103)
Supplement: S1 File — (PDF) [file pone.0324103.s001.pdf]

Dec 02, 2024

The Mind & Muscles: A Protocol for the simultaneous measuring of cognitive and muscular activation during stone tool tasks using surface Electromyography and Electroencephalography

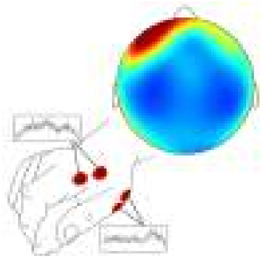

DOI

dx.doi.org/10.17504/protocols.io.36wgqnxbygk5/v1

Brienna Eteson[1], Simona Affinito[1], Fotios Alexandros Karakostis[1,2,3]

[1]DFG Center for Advanced Studies “Words, Bones, Genes, Tools”, Department of Geosciences, Eberhard Karls University of Tübingen, Tübingen, Germany;

[2]Paleoanthropology, Senckenberg Centre for Human Evolution and Palaeoenvironment, Department of Geosciences, Eberhard Karls University of Tübingen, Tübingen, Germany;

[3]Integrative Prehistory and Archaeological Science, University of Basel, Basel, Switzerland

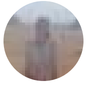

Brienna Eteson

DFG Center for Advanced Studies “Words, Bones, Genes, Tools”...

OPEN ACCESS

DOI: dx.doi.org/10.17504/protocols.io.36wgqnxbygk5/v1

Protocol Citation: Brienna Eteson, Simona Affinito, Fotios Alexandros Karakostis 2024. The Mind & Muscles: A Protocol for the simultaneous measuring of cognitive and muscular activation during stone tool tasks using surface Electromyography and Electroencephalography. **protocols.io** **https://dx.doi.org/10.17504/protocols.io.36wgqnxbygk5/v1**

License: This is an open access protocol distributed under the terms of the **Creative Commons Attribution License**, which permits unrestricted use, distribution, and reproduction in any medium, provided the original author and source are credited

Protocol status: Working
We use this protocol and it's working

Created: October 12, 2024

Last Modified: December 02, 2024

Protocol Integer ID: 109742

Keywords: EEG, EMG, stone tools, human evolution, cognition, muscular activation

Funders Acknowledgement:
Deutsche
Forschungsgemeinschaft
(DFG)
Grant ID: DFG FOR 2237

Disclaimer

DISCLAIMER – FOR INFORMATIONAL PURPOSES ONLY; USE AT YOUR OWN RISK

The protocol content here is for informational purposes only and does not constitute legal, medical, clinical, or safety advice. Information presented in this protocol should not substitute for independent professional judgment, advice, diagnosis, or treatment. Any action you take or refrain from taking, using, or relying upon the information presented here is strictly at your own risk. You agree that neither the Institute nor any of the authors, contributors, administrators, or anyone else associated with **protocols.io**, can be held responsible for your use of the information contained in or linked to this protocol or any of our Sites/Apps and Services.

Abstract

This protocol presents the first detailed step-by-step pipeline for a combined methodology to record and pre-process data from surface electromyography (sEMG) and electroencephalography (EEG) simultaneously, in experiments focusing on the evolution of human manual behavior (e.g., stone tool use). This integrative approach enables monitoring both muscular and cognitive activation during specific stone tool tasks, allowing for accurate combined analysis of both functions to the millisecond. Data collection and preprocessing are conducted using BrainVision hardware and software (Brain Products GbmH, Gilching, Germany) [1,8]. **BrainVision Recorder (version 1.24.0101)** [1] captures the sEMG and EEG signals, while **BrainVision Analyzer (version 2.2.1)** [8] was utilized for cleaning and pre-processing the data.

This protocol outlines an experiment monitoring participants during a simple and widely studied stone tool task: Oldowan flake cutting. This task uses replica Oldowan flakes, due to the tools' importance as one of the first sharp-edged tools within the hominin archaeological record. Participants are required to hold, aim, and then accurately cut pieces of faux leather using the stone tool, while their muscular and brain activation levels are recorded.

Image Attribution

EEG FFT Top Band Mapping View and sEMG averaged DI1 and TE signal of the Flake Aim stage from **BrainVision Analyzer software (version 2.2.1, Brain Products GbmH, Gilching, Germany)** [8]. Modified sketch from Eteson et al. [12] demonstrating "precision” (pad-to-side) grip, as in the Flake task. Modified in **Inkscape vector graphics editor (version 1.3)**. [97].

Guidelines

This protocol describes the entirety of the process of simultaneously measuring cognitive and muscular activity using Electroencephalography (EEG) and surface Electromyography (sEMG), from set-up and application to recording and processing of data. Examples of the software and hardware used are provided within each step. To apply this protocol to multiple muscles or brain regions, repetition of the steps, as mentioned in the protocol is crucial.
For further explanation of why certain 'Parts' or sections are essential to the process, refer to the 'Note' posted before the first step. All additional information is included in a 'Note' under a step.

In Parts 2, 4, and 5, there are two Step-Cases, accessible in the drop-down menu. These are broken down into the respective methodologies outlined in this protocol; EEG and sEMG. Whilst the methods are recorded simultaneously, they must be applied, pre-processed, and analyzed separately. Select Step-Case 1 for EEG and Step-Case 2 for sEMG. To correctly follow the entire protocol, return to the EEG Step-Case to view the relevant steps for both EEG and sEMG in Parts 3, 4, and 5, as it is not possible to continue to these from the sEMG Step-Case.

Materials

The standard EMG and EEG set-up consists of the following materials:

- 10 ml LuerLock Solo syringes (ref: 4617100V, Braun Omnifix)
- 34 actiCAP slim active electrodes system (ref: BP-135-1501/BP-235-2120/BP-235-2110, 32 channels + 1 ground and 1 reference electrode)
- Abrasive Electrolyte-Gel (ref: 219-001-6-R, EasyCap Abralyt HiCl)
- Alcohol wipes N94842 (ref: 501 075, Winner Medical Co., LTD)
- Baseline BIMS grip and pinch strength dynamometers (ref: 12-0092/12-0072/12-0082, functional model, Fabrication Enterprises)
- Blunted needles (ref: DISP0001800, Spec Medica)
- BrainVision Analyzer software (version 2.2.1, Brain Products GbmH, Gilching, Germany)
- BrainVision BIP_2AUX Adapters (ref: label 001 11/2014)
- BrainVision LiveAmp actiCAP Adapter (ref: BP-210-2100)
- BrainVision LiveAmp Sensor and Trigger Extension (STE) (ref: BP-210-2000)
- BrainVision LiveAmp USB Bluetooth Adapter (model: SE-UBT21-1)
- BrainVision LiveAmp wireless amplifier (ref: BP-200-3000)
- BrainVision Recorder software (version 1.24.0101, Brain Products GbmH, Gilching, Germany)
- Cartridge Press (ref: MG350, Wolfcraft GmbH)
- Cotton wool swabs
- EasyCap 32Ch actiCAP snap cap (CLAPS-32-SCMW-various sizes)
- EasyCap Multitrode electrodes B18 (ref: B18-HSR-120)
- EasyCap SuperVisc High-Viscosity Electrolyte-Gel for Active Electrodes (ref: 719-001-5-R)
- Finger cots (size: medium, finger gloves without latex)
- Kinesiotape TrueTape (True Tape Sports GmbH)
- LiveAmp Belt System (Brain Products GbmH, Gilching, Germany)
- MES flexible/fabric tape measure (MES Forschungssysteme GmbH)
- Plastic colander/strainer
- PowerCore 13000 (model: A1215, Anker)
- Sealant gun
- Toothbrush (article number: 2540A9, Prokudent)

The use of additional recording or analyzing software (beyond the ones already used) is possible, although this may inevitably result in individual necessary changes to be made to the presented protocol. All materials and equipment are outlined within this protocol. We recommend that you familiarize yourself with the BrainVision software and hardware, and thoroughly read the protocol prior to application, as some steps relate to one another.

Materials for the experimental task presented in this protocol and associated validation paper:

- Pleather (faux leather)
- Marker pen
- Wooden chopping board
- Large sandbag
- Foam rectangle
- Scissors
- Macadamia nuts

- Oldowan replica Senonian flint flakes produced using hard hammer percussion (5 – 7 cm in length [94,95])
- Quartzite Hammerstones (8 – 14 cm in length [96])

Safety warnings

Participants must have no known cognitive conditions, such as Attention Deficit Hyperactive Disorder (ADHD), Depression, etc., due to the known disruptive effects on frequencies in the electroencephalography (EEG) brain waves [88,89]. Participants were also excluded if they had been prescribed medication to treat any such cognitive conditions [90]. Participants must have no sight impairment or have corrected sight via the use of eyeglasses, contact lenses, laser eye surgery, or any additional sight corrective measures.

Participants were also asked to abstain from consuming caffeine and nicotine on the day of the experiment due to previous studies confirming both substances affect EEG power signals in certain frequency bands [91–93]. Moreover, due to the archaeological element of this experimental study, the use of stone tools, additional precautions should be taken to ensure that no injuries occur during the experiment. Participants were only able to partake in the study if they confirmed all tetanus vaccinations were up-to-date and were instructed to wear disposable finger gloves during the task to ensure safety guidelines were adhered to throughout.

A supplementary video detailing the above, outlining the experimental process, including expectations of what the task involves, and the application of EEG and sEMG, was provided to all participants at least 48 hours before the experiment.

Ethics statement

To ensure standard scientific practices and ethical considerations are upheld, several criteria must be met before participation is accepted. The recommendations in this protocol are in line with the approvals made by the Ethics Committee of the University of Tübingen (in line with the Declaration of Helsinki, 1964, revised in 2013). Participants voluntarily filled out a self-evaluated health check to confirm no current injury to their dominant or non-dominant hand and forearm was obtained at the time of participation, and that all previous injuries were fully recovered.

Before start

This protocol was developed to contribute to the expanding interdisciplinary field of experimental archaeology, specifically aimed at enhancing our understanding of the relationship between cognitive processes and biomechanical activation during stone tool use. By exploring these patterns through rigorous experimental approaches, we can gain valuable insights into how early humans engaged with their environment and refined their skills. This protocol outlines all preparatory stages, cautionary guidelines, experimental setup, recording, and processing of simultaneously recorded EEG and sEMG.

For the experimental analysis used to validate this protocol, we have chosen a simple cutting task, using replica Oldowan flake tools. These tools, dating back to as early as ~3 MYA [84], are often used in experimental studies [12–14] as they represent one of the earliest tool industries in the hominin archaeological record [85,86] and the oldest direct evidence of the use of a forceful pad-to-side precision grip [87]. Participants were asked to perform a three-step task, to pick up the flake, aim the flake at the target, and execute the task by performing three cutting actions onto a piece of faux leather.

Part 1 - Recording Software Setup

1 **Hardware Setup**

Prior to opening the **BrainVision Recorder software (version 1.24.0101, Brain Products GbmH, Gilching, Germany)** [1], ensure the following has been performed:

1.1 Insert a memory card into the **BrainVision LiveAmp 24-bit amplifier (ref: BP-210-2000/BP-200-3000)** card slot.

> Note
>
> In our experiment, the **BrainAmp DC amplifier** (stationary) was used. However, we propose a protocol for recording data with **BrainVision LiveAmp 24-bit amplifier (ref: BP-210-2000/BP-200-3000)** (mobile), as it enables mobile EEG applications—a significant advantage for experimental archaeology studies that aim to capture brain and muscle activity. While the outputs and steps to perform are essentially the same, one difference is **BrainAmp DC**'s capability to record at sampling rates exceeding 1000 Hz. Nonetheless, a sampling rate of 1000 Hz is more than adequate for both EEG and EMG recordings.

1.2 Connect the **BrainVision LiveAmp Sensor and Trigger Extension (STE) (ref: BP-210-2000)** to the **BrainVision LiveAmp 24-bit amplifier (ref: BP-210-2000/BP-200-3000)** and **USB-Power bank (PowerCore 13000 (Anker model: A1215))**.

1.3 Connect the **BrainVision LiveAmp actiCAP Adapter (ref: BP-210-2100)** cable, along with the ground and reference electrodes for EEG to the **BrainVision LiveAmp 24-bit amplifier (ref: BP-210-2000/BP-200 3000)**.

1.4 Ensure all eight **BrainVision BIP$_2$AUX adapters (ref: label 001 11/2014)** are connected to the **BrainVision LiveAmp Sensor and Trigger Extension (STE) (ref: BP-210-2000)**. The number of adapters should correspond with the number of sEMG electrodes (muscles) monitored in this experiment.

> Note
>
> Optionally, a trigger can be added using an additional auxiliary (AUX) channel. This AUX can be connected to the computer to play a trigger sound to notify participants of a task's beginning or end. This can improve pre-processing for experiments such as event-related potentials (ERPs). In our experiment, we used a .mp4 file of a repeating 5-second beeping noise to notify participants of the start of each stage within the stone tool task.

1.5 Place two electrodes (+ and −) into each **BrainVision BIP$_2$AUX adapter (ref: label 001 11/2014)**.

Note

Pair cables by color to simplify the sEMG application process (see figure 1).

1.6 Add one additional electrode to one **BrainVision BIP_2AUX adapter (ref: label 001 11/2014)**. This electrode should be placed in the center **GND** AUX.

1.7 Attach all 34 **actiCAP slim electrodes (32 channels + 1 ground and 1 reference channel, ref: BP-135-1501)** to the **EasyCap 32Ch actiCAP snap cap (CLAPS-32-SCMW-various sizes)**.

Note

This protocol uses the international 10-20 system with 32 channels. However, adjustments to the number of electrodes used can be made easily while following this protocol.

The number of electrodes (i.e. 32) used within this protocol is relatively low compared to other EEG studies that study voluntary motor tasks, including tool use [2–4]. However, our research has been shown to provide meaningful results [5]. Using fewer channels also enabled us to save time on application and ensured we met the guidelines of our ethics approval.

1.8 Place the ground and reference electrode in the pre-defined channels, **FCz** and **GND** as determined by the standard 10-20 32-channel antiCAP snap positioning [1].

2 Software Setup

2.1 Set up the computer using the **Brain Products Recorder Dongle (version 1.24.0101, Brain Products GbmH, Gilching, Germany)** [1] and **BrainVision LiveAmp USB Bluetooth Adapter (model: SE-UBT21-1)**.

2.2 Ensure all LiveAmp components are switched on and properly connected. This system is composed of five items: **BrainVision LiveAmp 24-bit amplifier (ref: BP-210-2000/BP-200-3000)**, **BrainVision LiveAmp actiCAP Adapter (ref: BP-210-2100)**, **BrainVision LiveAmp Sensor and Trigger Extension (STE) (ref: BP-210-2000)**, **BrainVision LiveAmp USB Bluetooth Adapter (model: SE-UBT21-1)**, and a **USB-Power bank (PowerCore 13000 (Anker model: A1215))**.

2.3 Attach all components, excluding the **USB Bluetooth Adapter (model: SE-UBT21-1)**, to the **LiveAmp Belt System (Brain Products GbmH, Gilching, Germany)**, which allows the participant to move freely during the experiment (see figure 1).

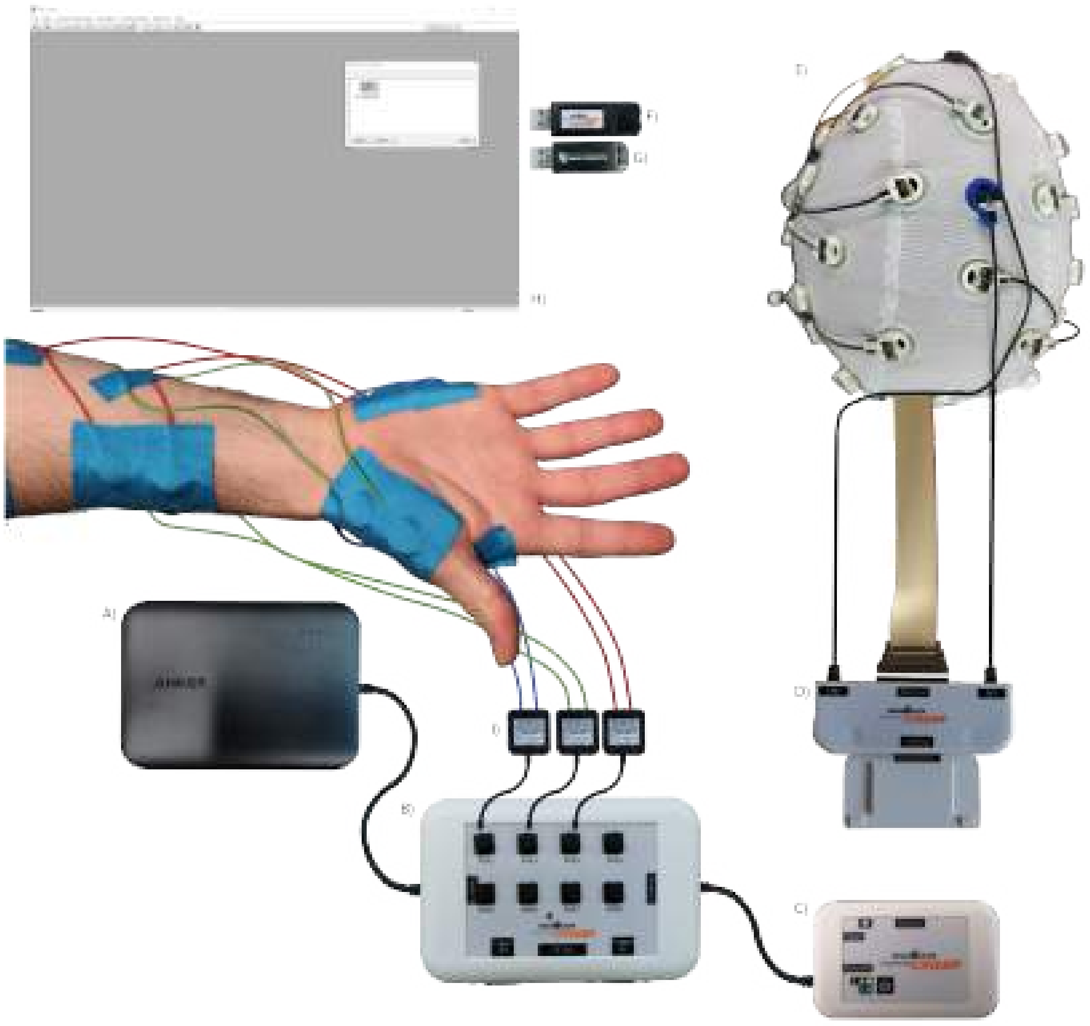

Fig 1. Components for EEG and sEMG setup:
A) **USB Power bank: PowerCore 13000 (model: A1215, Anker)**
B) **BrainVision LiveAmp Sensor and Trigger Extension (STE) (ref: BP-210-2000)**
C) **BrainVision LiveAmp wireless amplifier (ref: BP-200-3000)**
D) **BrainVision LiveAmp actiCAP Adapter (ref: BP-210-2100)**
E) **EasyCap 32Ch actiCAP snap cap (CLAPS-32-SCMW- various sizes) with EasyCap Multitrode electrodes B18 (ref: B18-HSR-120)**
F) **BrainVision LiveAmp USB Bluetooth Adapter (model: SE-UBT21-1)**
G) **BrainVision Recorder** dongle
H) **BrainVision Recorder software (version 1.24.0101, Brain Products GbmH, Gilching, Germany)**
I) **BrainVision BIP$_2$AUX Adapters (ref: label 001 11/2014)**

2.4 Open **BrainVision Recorder software (version 1.24.0101, Brain Products GbmH, Gilching, Germany)** [1] on your computer. Ensure the **BrainVision Recorder** dongle and **BrainVision LiveAmp USB Bluetooth Adapter (model: SE-UBT21-1)** are properly connected to the computer USB port.

2.5 On the LiveAmp Console window, choose **Search for LiveAmp** and select your amplifier (see figures 2 and 3).

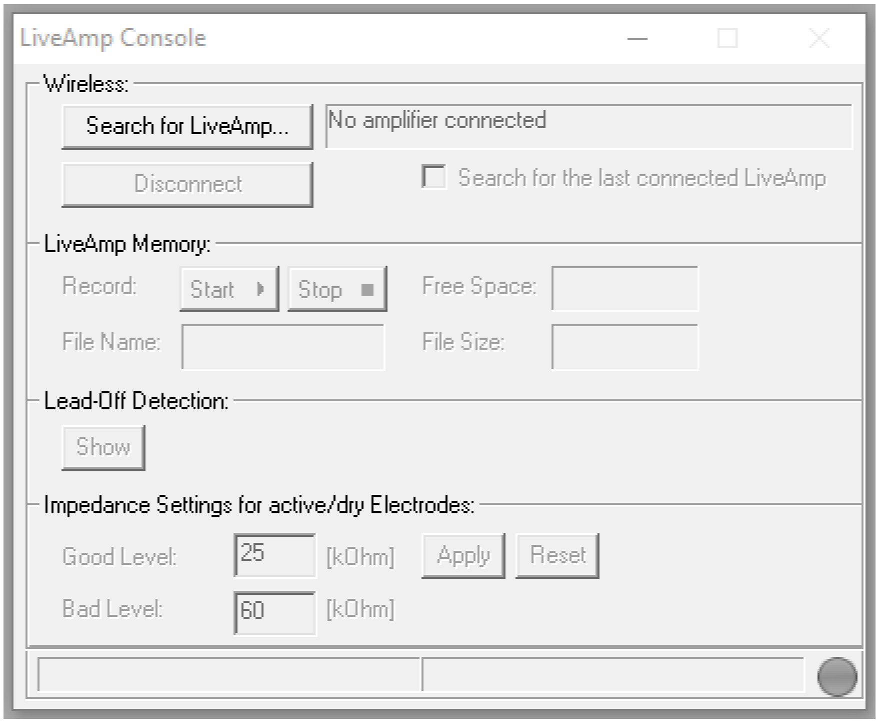

Fig. 2 **LiveAmp Console** window on Windows PC.

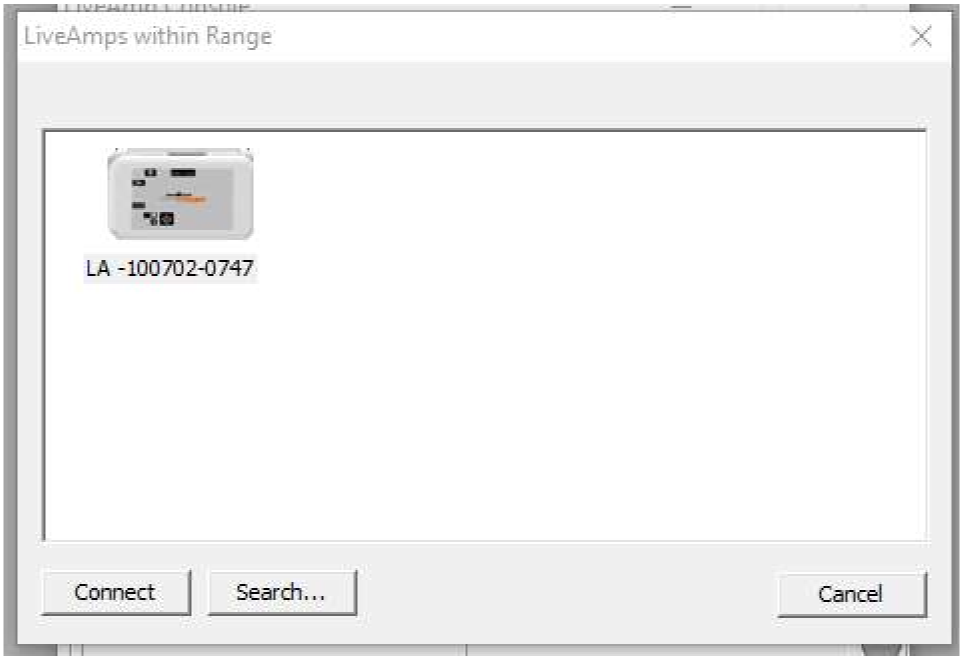

Fig 3. LiveAmp search window on Windows PC.

Note

BrainVision LiveAmp wireless amplifiers (ref: BP-200-3000) can be identified by their serial numbers.

2.6 The **BrainVision LiveAmp wireless amplifier (ref: BP-200-3000)** is properly connected when its wireless LED blue light is flashing.

2.7 Monitor the connection quality in the bottom right-hand corner of the **LiveAmp Console** window, (green=good, yellow=weak, red=bad) (see figure 4).

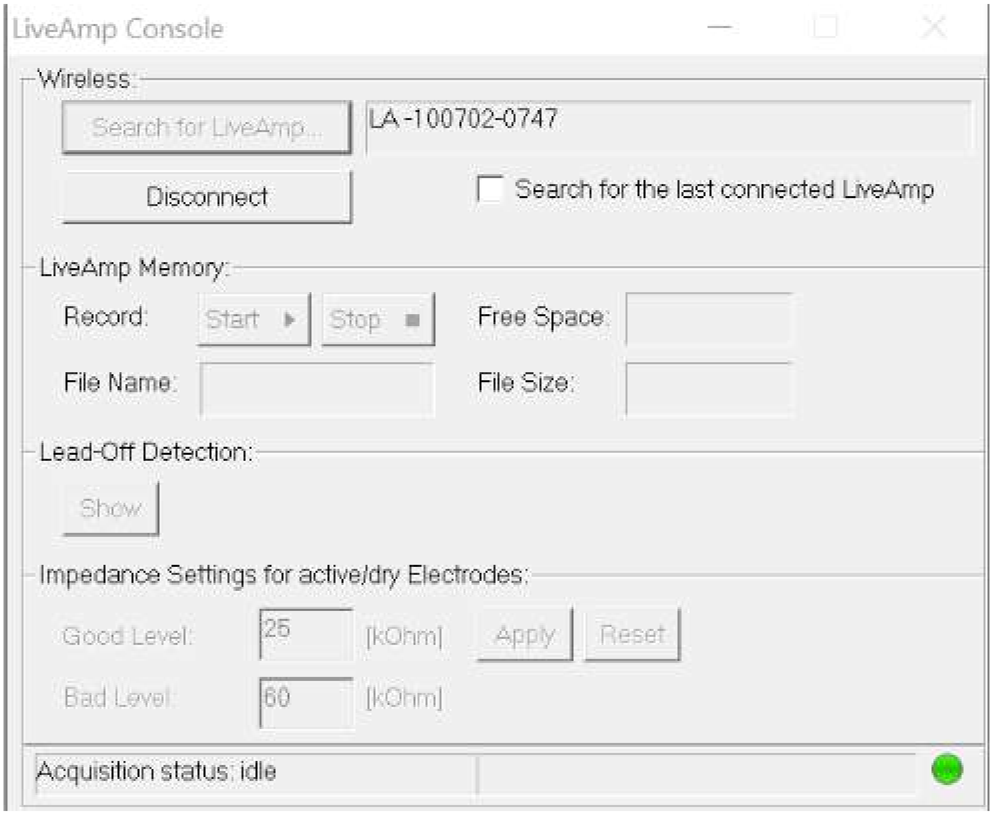

Fig. 4. **BrainVision LiveAmp wireless amplifier (ref: BP-200-3000)** successfully connected to the **BrainVision Recorder software (version 1.24.0101, Brain Products GbmH, Gilching, Germany)** [1].

2.8 Go to **File** and select **New Workspace**. A pop-up window should appear. Specify the destination directory for the EEG data in the **Raw File Folder**.

2.9 The **Amplifier Settings** contains amplifier-specific parameters and the channel table. Import the correct electrode names, topographies, and physical channels by navigating to **Use Electrode Position File** in the bottom left-hand corner. Check the box **Read positions from Electrode Position File** (see figure 5). **Browse** to locate the .bvef file format.

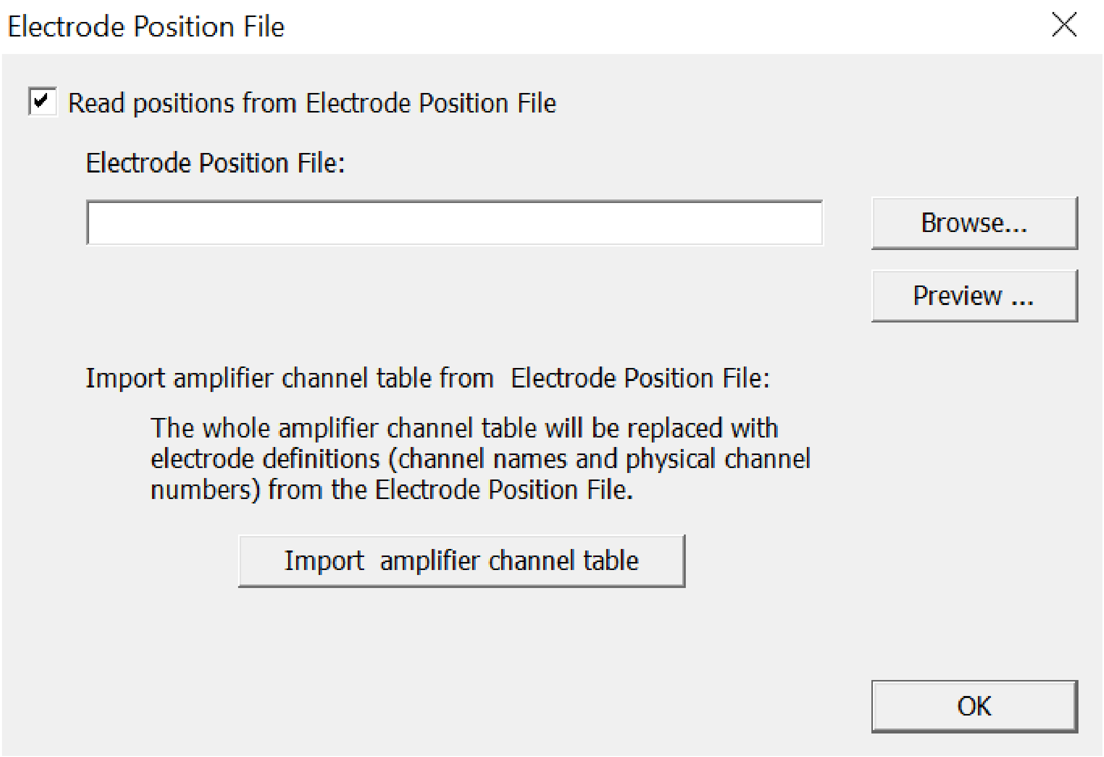

Fig. 5. **Electrode Position File** search window on Windows PC.

2.10 Select **Import amplifier channel table**. Recorder loads this file every time a new or existing **Workspace** is opened. To stop the automatic import, deselect the **Read positions from Electrode Position File** box.

2.11 On the main **Amplifier Settings** window, select the number of channels for EEG (i.e. 32) and set the sampling rate between 500 Hz – 1000 Hz [6,7] (see figure 6). EEG and EMG require a sampling rate of at least two times greater than the Nyquist frequency to ensure adequate sampling [6].

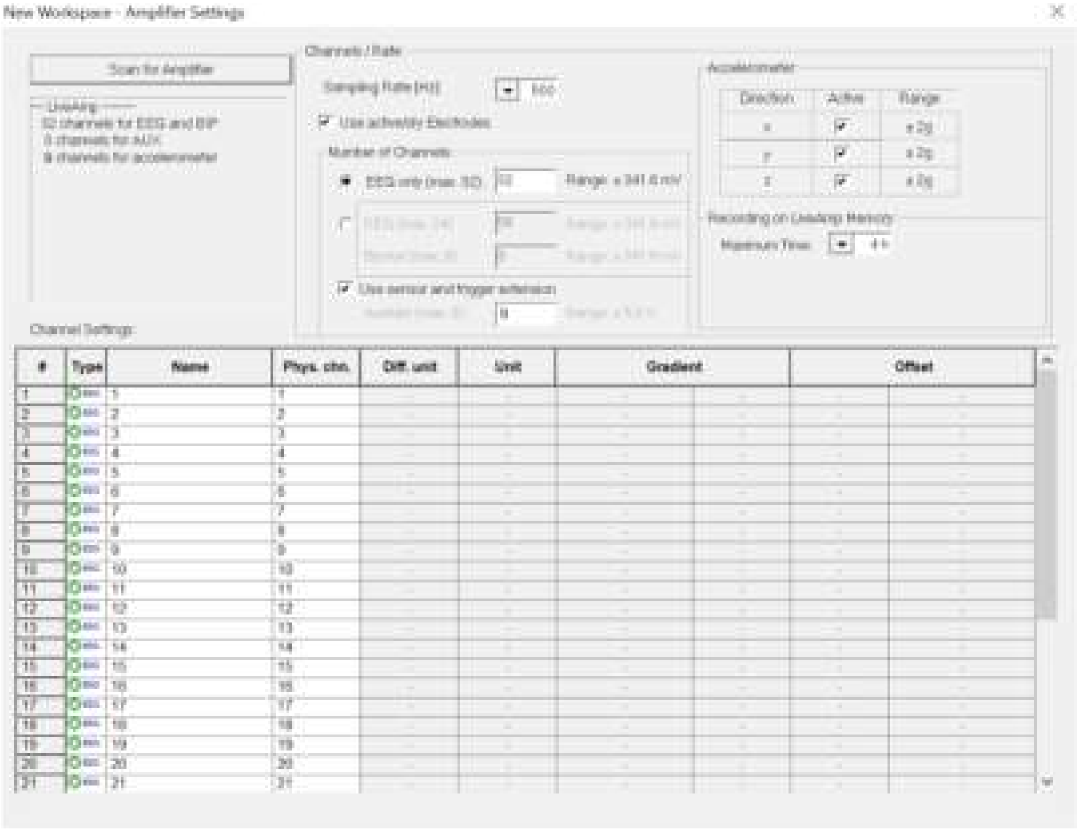

Fig. 6. **Amplifier Settings** window with recommended parameters.

2.12 Ensure the **Use active/dry Electrodes** and **Use sensor and trigger extension** boxes are checked. Add the number of AUX channels used in the experiment (i.e. number of muscles monitored).

2.13 Scroll down to the AUX channels and rename each channel to each corresponding muscle that will be recorded. Update all AUX channels **Unit** to "µV" and **Gradient** to "0.1" (see figure 7).

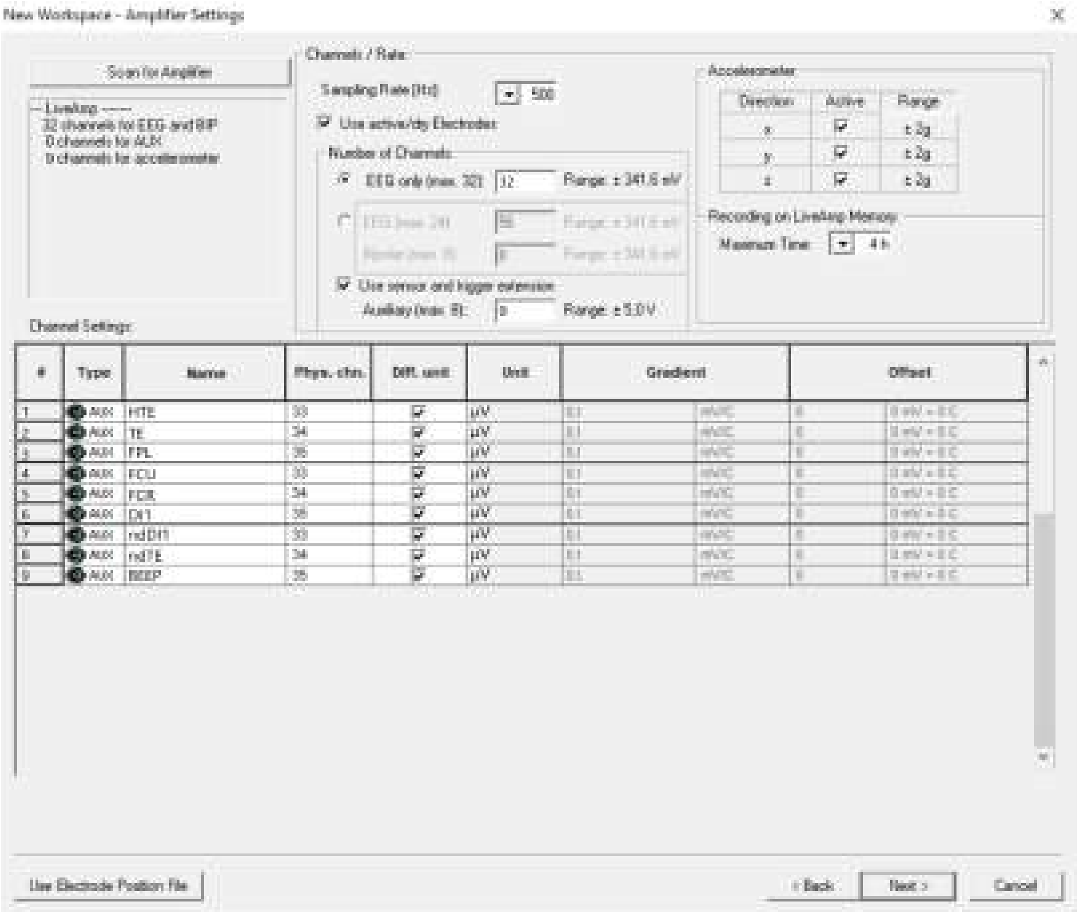

Fig. 7. Edit AUX channels **Name**, **Unit**, and **Gradient**.

2.14 Optionally, in **Filter Settings** you can apply a **Display Filter**. Select **Enable Filters**, then select "50 Hz" in the **Notch Filter** drop-down menu [1] (see figure 8). This filters the noise from the main line and varies depending on your region, either 50 Hz (e.g. Germany), or 60 Hz (e.g. the United States). Select **Use Individual Settings** and enable the notch filter for all EEG channels. This is a feature that allows you to switch between viewing the data filtered or unfiltered instantaneously during the recording, but the raw data will remain unaffected. Do not apply any other filters, this can be done post-recording in the **BrainVision Analyzer software (version 2.2.1, Brain Products GbmH, Gilching, Germany)** [8].

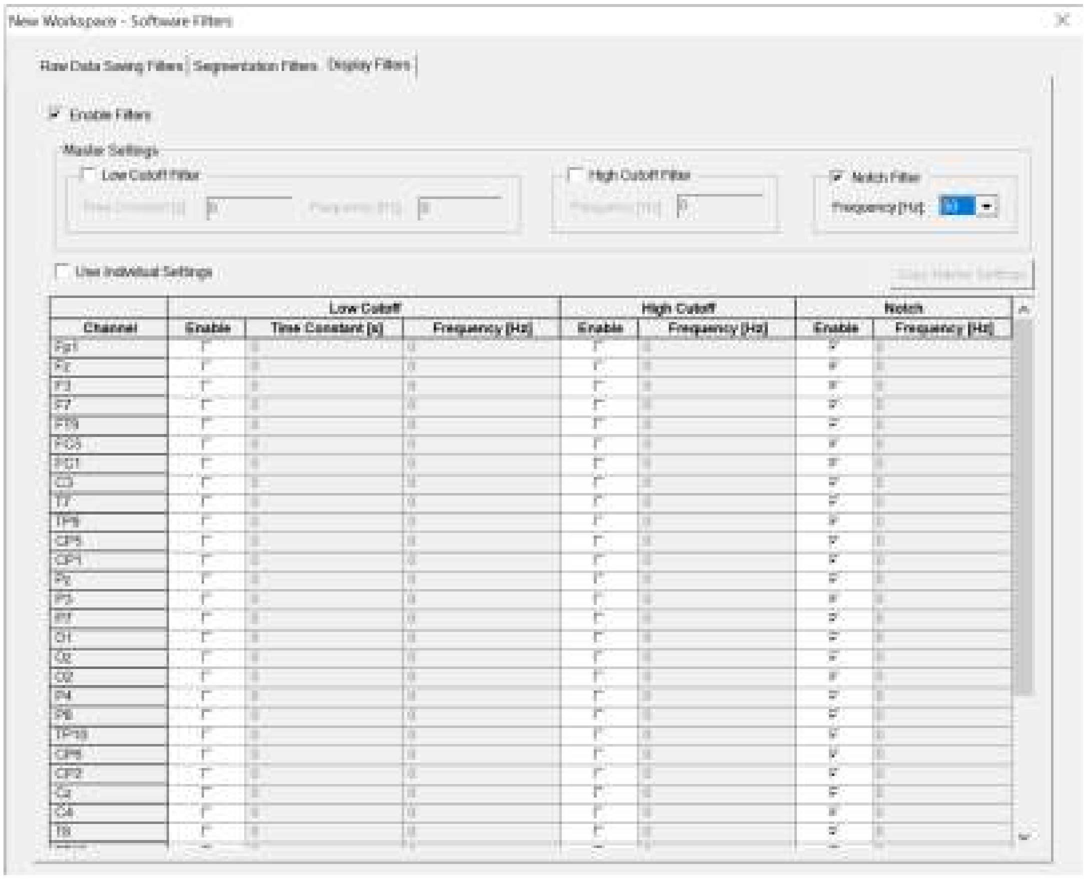

Fig. 8. Enabling display filters in the **Software Filters** window.

2.15 Leave the dialog box **Segmentation/Averaging** blank and select **Finish**.

2.16 Once the workspace is set up, the application of the EEG and sEMG can begin. Navigate to the **Impedance Check** button (electrode icon) on the toolbar and begin the application of the EEG and sEMG electrodes.

3 Placement Preparation

Note

The following Part consists of two Step-Cases, one for each methodology outlined in this protocol. Select Step-Case 1 for EEG and Step-Case 2 for sEMG. To follow the entire protocol, return to the EEG Step-Case, as it is not possible to continue to Parts 3, 4, and 5 from the sEMG Step-Case 2.

Note

EEG and sEMG electrodes must be applied with care as improper treatment can result in
inaccurate or unusable data.

STEP CASE

Electroencephalography From 73 to 122 steps

3.1 Before EEG application ensure the participant's hair is clean, with no products or hairstyling equipment in place.

3.2 Take the following three measurements of the participant's head (in cm): circumference of the head, nasion to inion, and between either ear-channel opening [23].

3.3 The participant's head circumference determines the appropriate **EasyCap 32Ch actiCAP snap cap (CLAPS-32-SCMW)** size. Place the corresponding sized cap onto the participant's head.

Note

Participant's head circumference (measured around the occiput and over the supraorbital ridges [9]) should be measured to determine the appropriately sized **EasyCap 32Ch actiCAP snap cap (CLAPS-32-SCMW)**. Measurements should be taken in centimeters (cm). Caps are sized in even numbers, e.g. 54/56/58/60. If participants' head circumference is an odd number, +1 to this measurement and use the corresponding cap size (i.e. *55 cm head circumference + 1 = cap size 56*).

3.4 The two other measurements taken ensure symmetrical and accurate placement of the cap, which in turn ensures accurate placement of the electrodes. Ensure the electrode channel **Cz (no. 24)** is positioned centrally between the two points of the following measurements: Nasion to Inion, and between ear-channel openings [23]), see figure 12.

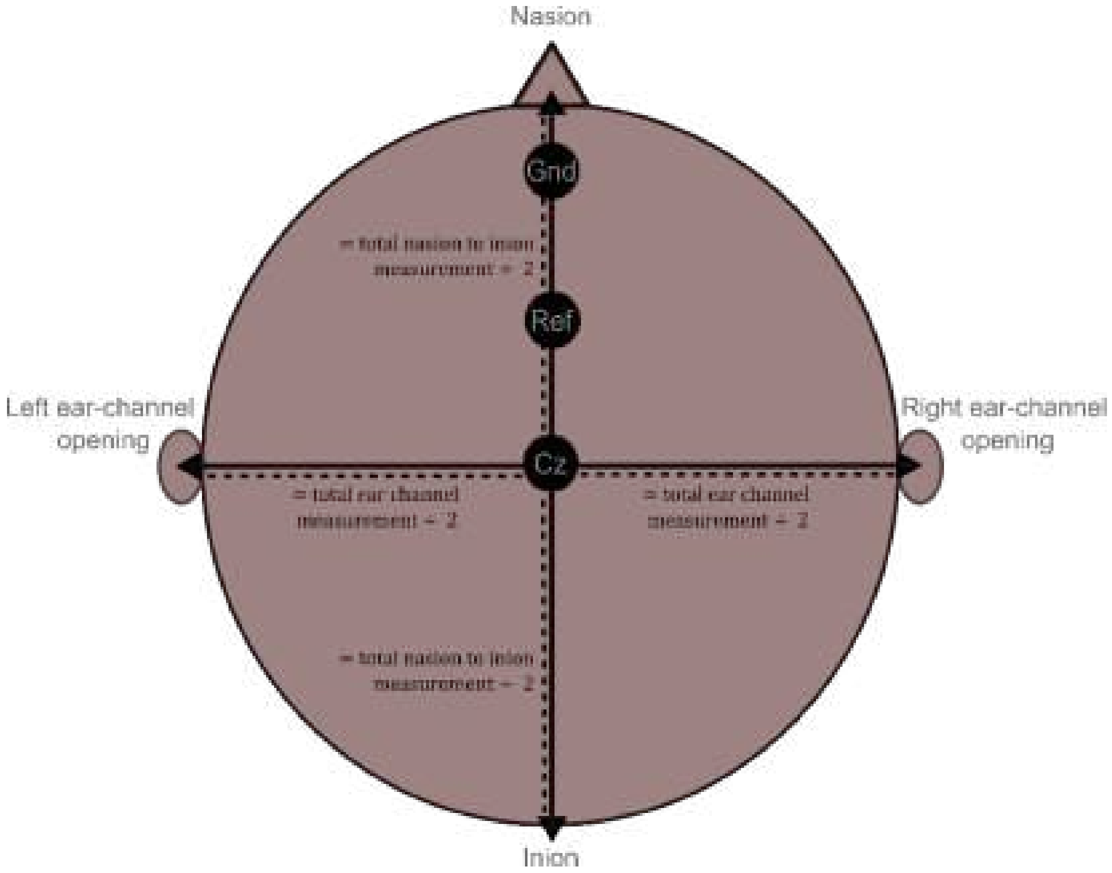

Fig. 12. 10-20 system cap placement measurement. Based on EEG EasyCap Cap Handling Flyer [23].

Note

Correct placement of EEG is assisted by the internationally recognized electrode positioning 10-20 system (The 10-20 system refers to the spatial orientation between adjacent electrodes, which are either 10% or 20% of the distance, from left-to-right or front-to-back, of the skull [1,24]), which ensures placement standardization. Additionally, a ground and online reference channel are placed in the pre-defined channels, **FCz** and **GND** [25], respectively. The 10-20 system ensures equidistance between each electrode, proportional to the shape and size of the individual's head, and broadly captures all brain regions [24]. Although understudied within stone tool use, previous neurological research, using fMRI, PET, and fNIRS, has concluded that cognitive activation during stone tool production occurs primarily in the frontal, pre-frontal, temporal, and parietal regions [26–32].

4 **Application of Electrolyte Gel**

4.1 Once the cap is positioned correctly, navigate to **Impedance Check** (electrode icon) (see figure 13) on the **BrainVision Recorder software (version 1.24.0101, Brain Products GbmH,**

Gilching, Germany) [1]. All LEDs on the electrodes should light up in red and an interactive channel map should be displayed on your computer screen.

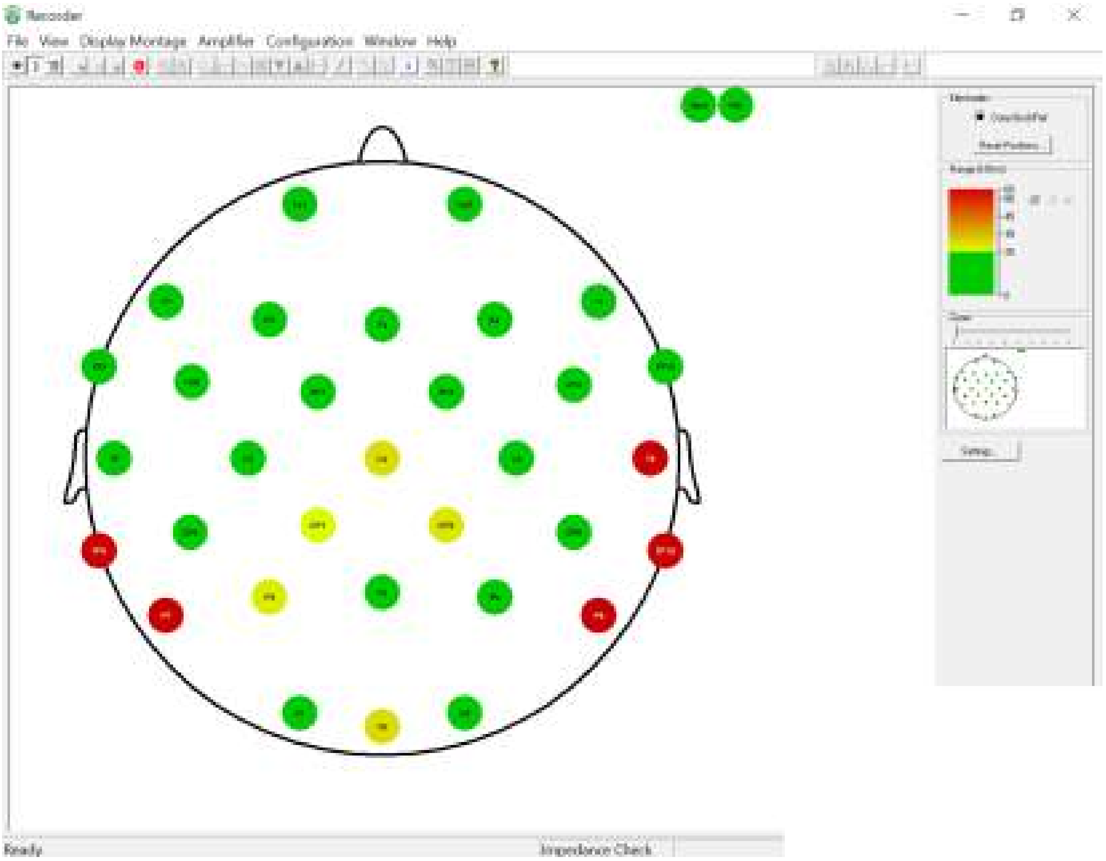

Fig. 13. Impedance Check in **BrainVision Recorder software (version 1.24.0101, Brain Products GbmH, Gilching, Germany)** [1] displaying 10-20 32-channel electrode positioning. All electrodes should ideally have an impedance of less than 10 kΩ (dark green) [33]. Red indicates impedance between 55 – 60 kΩ, and yellow indicates impedance between 25 – 45 kΩ.

4.2 Use a sealant gun to insert 10 ml of **EasyCap SuperVisc High-Viscosity Electrolyte-Gel for Active Electrodes (ref: 719-001-5-R)** into a **LuerLock Solo syringe (Braun Omnifix 10 ml ref: 4617100V)**.

4.3 Using the **LuerLock Solo syringe (Braun Omnifix 10 ml ref: 4617100V)**, begin inserting a small amount of Electrolyte-Gel into each electrode. It is crucial to begin with the Reference (FCz) and Ground (GND) electrodes.

4.4 Spread the **EasyCap SuperVisc High-Viscosity Electrolyte-Gel for Active Electrodes (ref: 719-001-5-R)** with the blunted needle of the **LuerLock Solo syringe (Braun Omnifix 10 ml ref: 4617100V)** inside the electrode, rotating it slowly in a circular motion to ensure good contact between the electrode sensor (placed below the LED) and the participant's scalp.

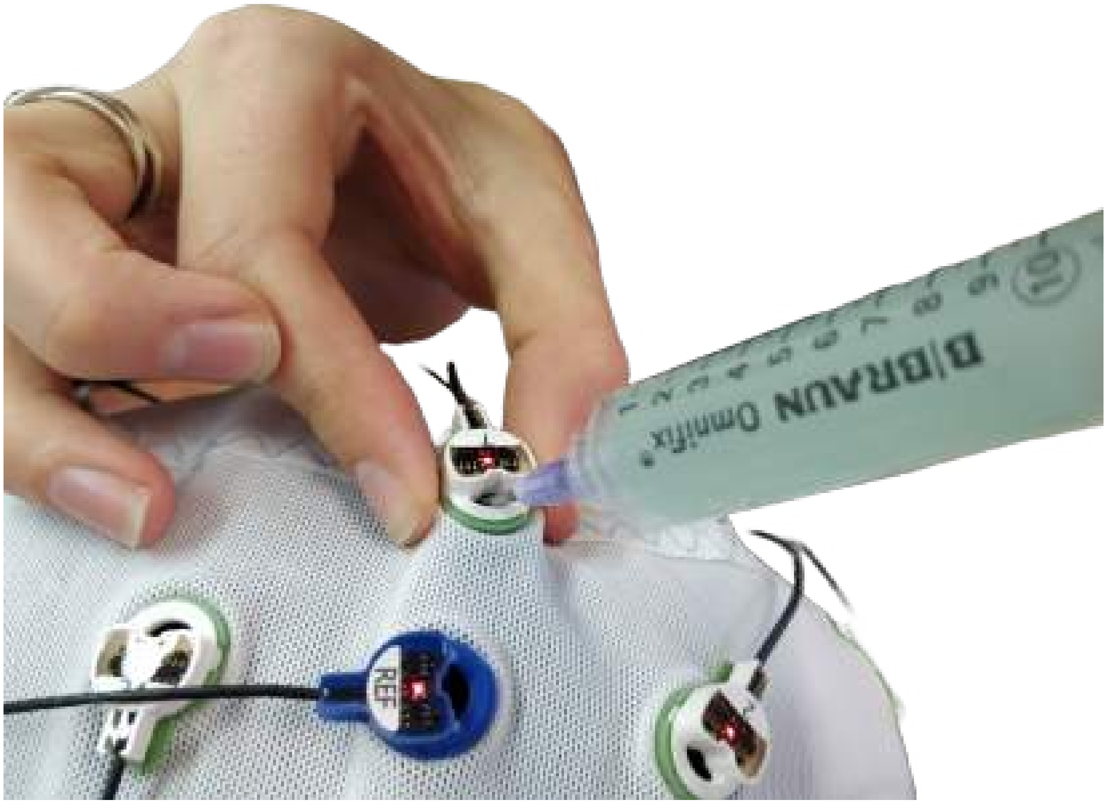

Fig. 14. Insertion of **EasyCap SuperVisc High-Viscosity Electrolyte-Gel for Active Electrodes (ref: 719-001-5-R)** into an electrode on the **EasyCap 32Ch actiCAP snap cap (CLAPS-32-SCMW)**.

4.5 Slowly remove the **LuerLock Solo syringe (Braun Omnifix 10 ml ref: 4617100V)**, whilst placing enough **EasyCap SuperVisc High-Viscosity Electrolyte-Gel for Active Electrodes (ref: 719-001-5-R)** into each electrode to ensure no air pockets are left once the syringe is removed (see figure 14). If electrodes continue to have poor impedance, repeat Steps 4.2 to 4.5 without over-filling and/or slightly pressing on the electrode.

Note

The LEDs should turn orange and then green as the impedance improves. All electrodes should have an impedance of less than 10 kΩ [33]. This is crucial to ensuring a high signal-to-noise ratio. The impedance between 25 and 60 kΩ remains orange. Usually, the impedance improves with time. Therefore, orange electrodes can be left and may become green whilst other electrodes are being filled.

Note

Bridging is a common problem to be aware of during EEG application. Impedance lower than 100 Ω often indicates bridging [34]. Bridging occurs when too much gel has been inserted into an electrode causing contamination between two neighboring electrodes. This results in these two or more bridged electrodes containing similar signals, introducing spatial smearing, meaning all bridged electrodes are no longer displaying their true signal [35,36].

Part 3 - Recording of the Stone Tool Task

5 **Pre-Recording Setup**

5.1 Navigate to the **Start Monitoring** (eye icon) on the toolbar.

5.2 Prior to recording, check all EEG and sEMG channels to ensure each channel is connected properly and displaying normal levels of activation (no dead or noisy channels, see figure 11). This helps to establish any abnormalities and enables you to fix them to ensure good data collection.

5.3 Adding markers can assist in segmentation during pre-processing of the data. Select **Predefined Annotations** under **Configurations** to timestamp specific stages or tasks within the experiment (see figure 15).

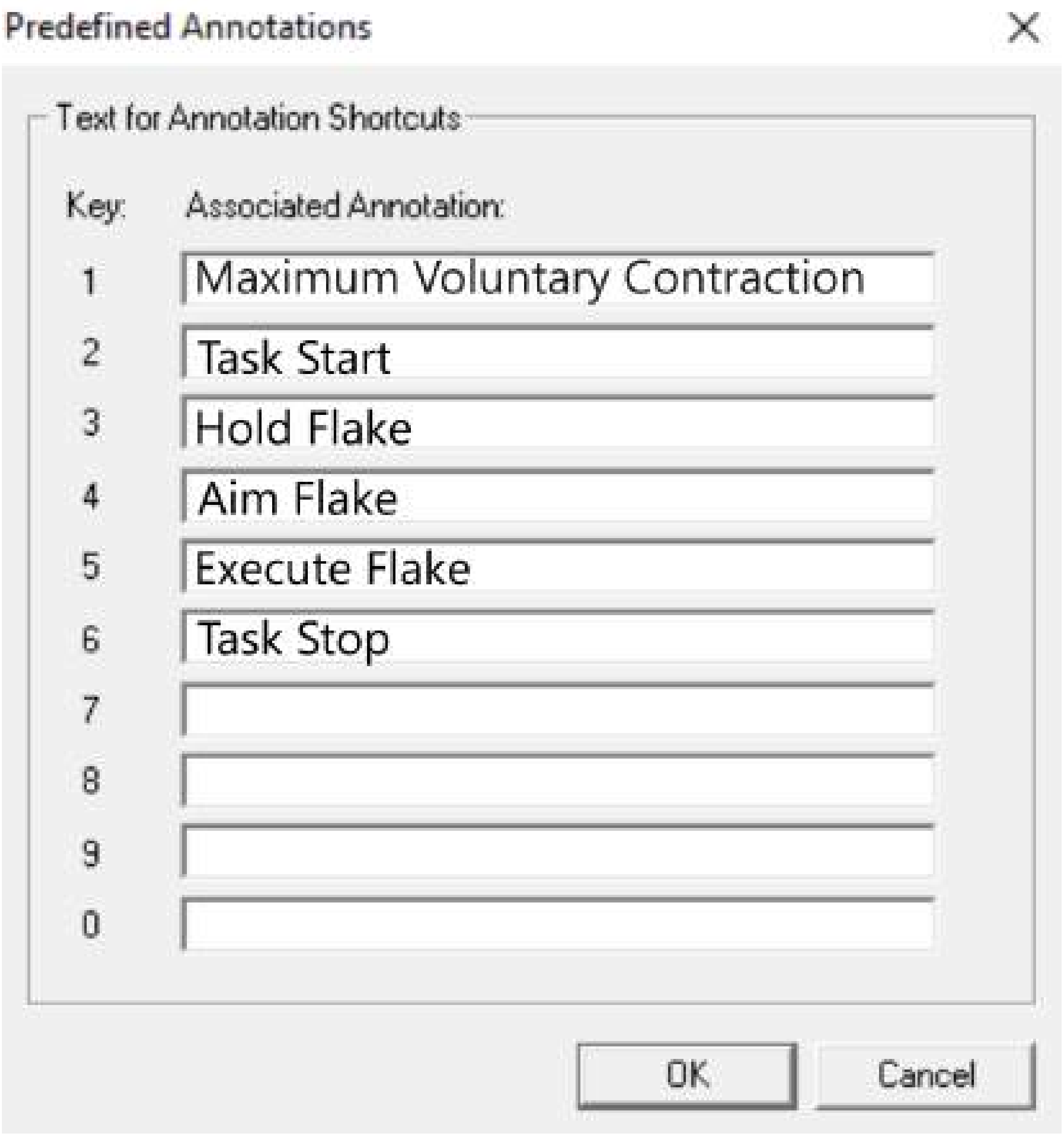

Fig. 15. **Predefined Annotations** window. Each annotation is associated with a keyboard key that, once pressed, triggers a timestamped marker in the data.

Note

Each **Predefined Annotation** is linked to a keyboard key, which when activated, correlates to a specific marker that is timestamped in the recorded data. Examples used in this protocol are: “Maximum Voluntary Contraction", “Task Start", "Hold Flake", "Aim Flake", "Execute Flake", and "Task Stop".

5.4 Once setup is complete, click the green play button to begin the recording. This creates a new **Workfile**. Once the **Workfile** has been saved, recording begins.

Note

During recording, insert any time-relevant notes, i.e. “participant movement causing noise/external artifacts”. These notes can then be viewed post-recording, and during pre-processing to help understand and improve the data recorded.

Note

In addition to **Predefined Annotations**, we recommend using an Experiment Sheet, detailing problems, and successes/failures during the experiment. This sheet should include the following information: when electrode contact occurred, excessive movement, late task initiation, external artifacts, and success, or failure of the task. These notes, alongside video recordings, help during the pre-processing of the data.

6 **Maximum Voluntary Contraction Task**

6.1 Before the experiment starts, take a total of six (3× pinch grip and 3× power grip) maximum voluntary contractions (MVC) readings.

Note

Ensure these readings are labeled with a **Predefined Annotation** so they are easily distinguishable from the experiment tasks or create a separate recording (**Workfile**) labeled as "Maximum Voluntary Contraction".

Note

MVCs are performed in EMG studies to ensure data is comparable between muscles, participants, and tasks [37]. MVCs are calculated using dynamometers. Participants perform strength tests by applying maximum force (pinch and grip). The dynamometer records the participants' strength (readings in kg or lbs). Additionally, the MVC data is used to determine the participants' maximum strength of each muscle. This amplitude is then used to transform the data taken during the experimental tasks into percentage maximum voluntary contractions (%MVCs). This allows all participants’ data to be comparable.

7 **Experimental Stone Tool Use Task**

7.1 Once MVC readings have been taken, begin the experimental task.

Note

In this protocol, we describe a simple stone tool-cutting task. Each stage is initiated by the trigger sound, which notifies the participant to begin. Once the trigger is sounded, the relevant **Predefined Annotation** is also activated by the experimenter. The trigger is sounded every five seconds, signaling the start of a new stage in the task. One complete task repetition is 20 seconds long; 5 seconds for each stage: "Hold", "Aim", and "Execute", and an additional 5 seconds for a "Rest" period. In the Oldowan flake-cutting task, participants must first pick up and hold the flake tool, referred to as "Hold". In the next five seconds, participants prepare and aim the flake at the faux leather square on the table, referred to as "Aim". In the final stage of the task, the cutting action is performed, i.e. cutting a "Z" pattern into the faux leather square, this is referred to as "Execute". The final stage marks a reset and rest period for the participant, known as "Rest". The entire task (including all four stages) is then repeated. This is performed a total of ≥50 times.

Note

For EEG recordings, it is recommended to perform at least 40 repetitions [38–40] in each task for every participant. Whilst this is only necessary for EEG, repetitions allow for the collection of more data on participant EMG muscular activation.

8 **Control Task**

8.1 Participants should also perform a simple motor control task, in addition to the stone tool task.

Note

A control task is a baseline composed of a simple motor action, designed to activate the motor cortex. This enables direct comparison between the control and stone tool task stages ("Hold", "Aim", "Execute"), by highlighting activation in common brain regions, and thereby isolating regions activated exclusively during stone tool use, for analysis [41]. The control task used in this protocol was a simple voluntary movement that involved opening and closing the dominant hand for five seconds. The task was then repeated ≥50 times.

8.2 After the experiment has ended and the recording has stopped, select the red (S) stop icon to save the **Workfile** and stop all monitoring.

Part 4 - Preprocessing of the Data

9 **Analyzer Setup**

9.1 Create a new folder for the EEG and sEMG files. In the folder, create four additional folders labeled: “Export”, “History”, “Raw”, and “Workspace”.

9.2 Copy all raw data files (.eeg, .vhdr, and .vmrk files) from each recording to the “Raw” folder. Ensure no changes are made to the file names, as they are linked internally.

> **Note**
>
> If using a dataset previously uploaded to **BrainVision Analyzer software (version 2.2.1, Brain Products GbmH, Gilching, Germany)** [8], note that **History** files are in the following file format, .ehst2 and .hfinf2.

9.3 Open **File** in **BrainVision Analyzer software (version 2.2.1, Brain Products GbmH, Gilching, Germany)** [8]. Select **New** to create a new workspace.

9.4 In the new window, use the browse function to select the folders you just created as the destination for each file type. These correspond to the pre-made folders e.g. “Export”, “History”, “Raw”, and “Workspace”.

9.5 Save this new workspace in the “Workspace” folder. The workspace is now set, and the raw data should be shown in the **Primary** window. Each folder corresponds to a participant’s recording.

> **Note**
>
> Ensure you are always working on the latest node. You can see the node you are working on directly above the dataset.

10 **Markers**

10.1 Use any video footage and Experiment Sheets to locate all recorded issues, such as artificial noise, electrode contact, failure to perform the task, or late task onset. Additionally, locate all notes made during the experiment. If any notes indicate an issue with the timing of the **Predefined Annotation** (i.e. late participant initiation of the task), remove this marker to avoid inaccurate data gathering.

10.2 To reposition markers, to correctly correspond to the trigger sound (displayed as an AUX channel) navigate to **Edit Markers** in **Dataset Preprocessing** under the **Transformation** tab.

10.3 Select **Graphical** view. A new node will be created. **Markers** can now be dragged to the correct timestamp, correlating to the trigger (see figure 16).

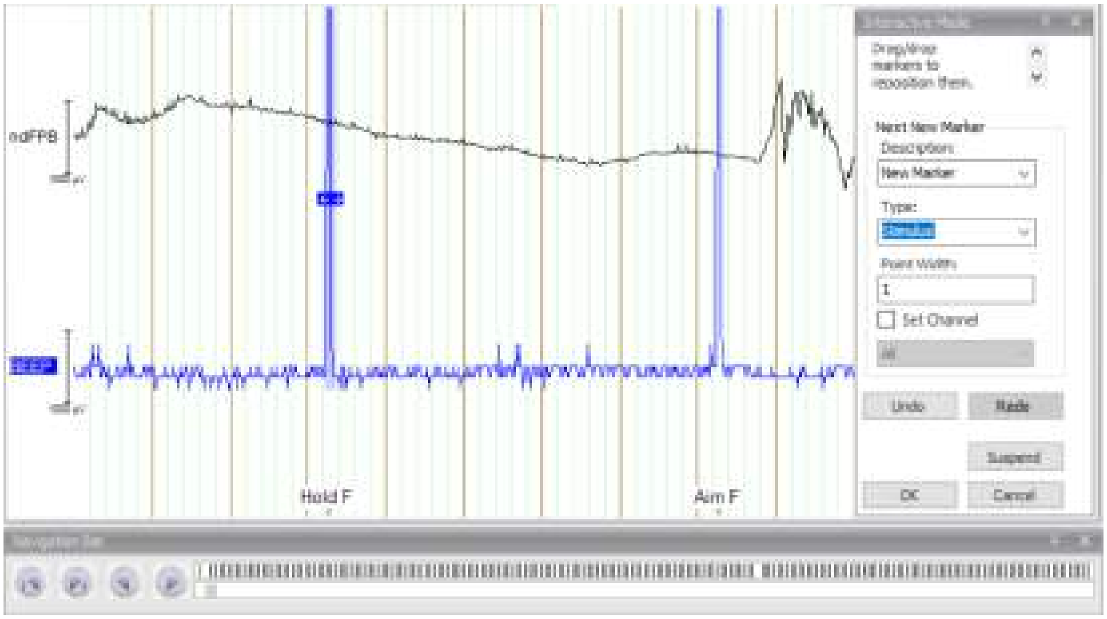

Fig. 16. **Edit Markers** node in **Graphical** view. Trigger AUX channel "BEEP" marking task onset. **Timestamped Annotations** are positioned at the exact moment the trigger sound occurred.

11 **Edit Channels**

Note

The following Part consists of two Step-Cases, one for each methodology outlined in this protocol. Select Step-Case 1 for EEG and Step-Case 2 for sEMG. To follow the entire protocol, return to the EEG Step-Case, as it is not possible to continue to Part 5 from the sEMG Step-Case 2.

STEP CASE

Electroencephalography

From 78 to 89 steps

Preprocessing is an essential part of collecting EEG data for analysis and visualization. The preprocessing steps mentioned in Part 4 are specific to our experimental design and objectives. Below, we provide steps implemented as part of our experimental pipeline, which should serve as a guide for performing your own experiment.

- Edit Channels
- Down Sample (Change Sampling Rate)
- Data Filtering
- Re-Reference
- Raw Data Inspection
- Independent Component Analysis (ICA)
- Inverse ICA
- Topographic Interpolation
- Segmentation
- Baseline Correction
- Condition Segmentation
- Artifact Rejection

- Fast Fourier Transformation (FFT)
- Averaging

11.1 Check the raw data and note all noisy or dead sEMG channels, that may require removal (see figure 11).

- Dead channels appear as a flat line.
- Noisy channels typically display repeating unpatterned, large spikes that are not mirrored in other channels.
- Clipping interference or saturation is when the amplitude of a signal reaches levels beyond the range that can be recorded. This can occur due to high signal amplification or improper electrode attachment to the skin [42].

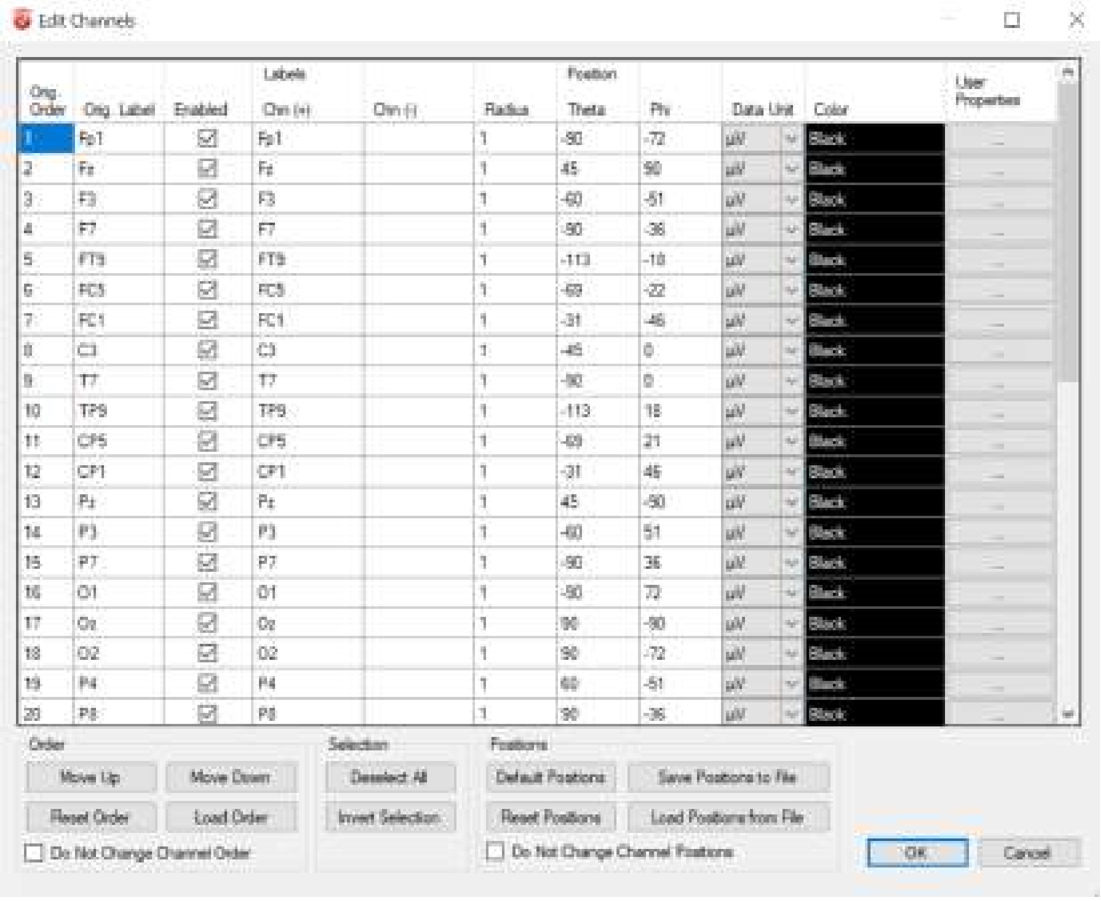

Orig. Order	Orig. Label	Enabled	Labels Chn (+)	Chn (-)	Radius	Position Theta	Phi	Data Unit	Color	User Properties
1	Fp1	☑	Fp1		1	-90	-72	µV	Black	...
2	Fz	☑	Fz		1	45	90	µV	Black	...
3	F3	☑	F3		1	-60	-51	µV	Black	...
4	F7	☑	F7		1	-90	-36	µV	Black	...
5	FT9	☑	FT9		1	-113	-18	µV	Black	...
6	FC5	☑	FC5		1	-69	-22	µV	Black	...
7	FC1	☑	FC1		1	-31	-46	µV	Black	...
8	C3	☑	C3		1	-45	0	µV	Black	...
9	T7	☑	T7		1	-90	0	µV	Black	...
10	TP9	☑	TP9		1	-113	18	µV	Black	...
11	CP5	☑	CP5		1	-69	21	µV	Black	...
12	CP1	☑	CP1		1	-31	46	µV	Black	...
13	Pz	☑	Pz		1	45	-90	µV	Black	...
14	P3	☑	P3		1	-60	51	µV	Black	...
15	P7	☑	P7		1	-90	36	µV	Black	...
16	O1	☑	O1		1	-90	72	µV	Black	...
17	Oz	☑	Oz		1	90	-90	µV	Black	...
18	O2	☑	O2		1	90	-72	µV	Black	...
19	P4	☑	P4		1	60	-51	µV	Black	...
20	P8	☑	P8		1	90	-36	µV	Black	...

Fig. 29. **Edit Channels** window. Only EEG channels selected.

Note

If channels that are noisy or have a signal deadline only occur during a few repetitions (i.e. >40 repetitions of the task remain useable), these sections can be removed at a later point and the channels can be kept in the dataset.

11.2 Remove noisy or dead channels from the analysis by navigating to **Edit Channels** under the **Transformations** tab. Deselect the relevant channels for removal. For EEG preprocessing, all sEMG channels should also be removed as preprocessing differs between the two methodologies (see figure 29). Additionally, deselect the acoustic marker channel (labeled "BEEP" here) used to define task beginning and/or end, to ensure only EEG channels are processed (see figure 17).

12 **Down Sample (Change Sampling Rate)**

12.1 Navigate to the **Transformations** tab and click **Change Sampling Rate**. The current sampling rate is shown under **Current Rate**. Enter the new sampling rate in **New Rate** and select **Spline Interpolation** (see figure 30).

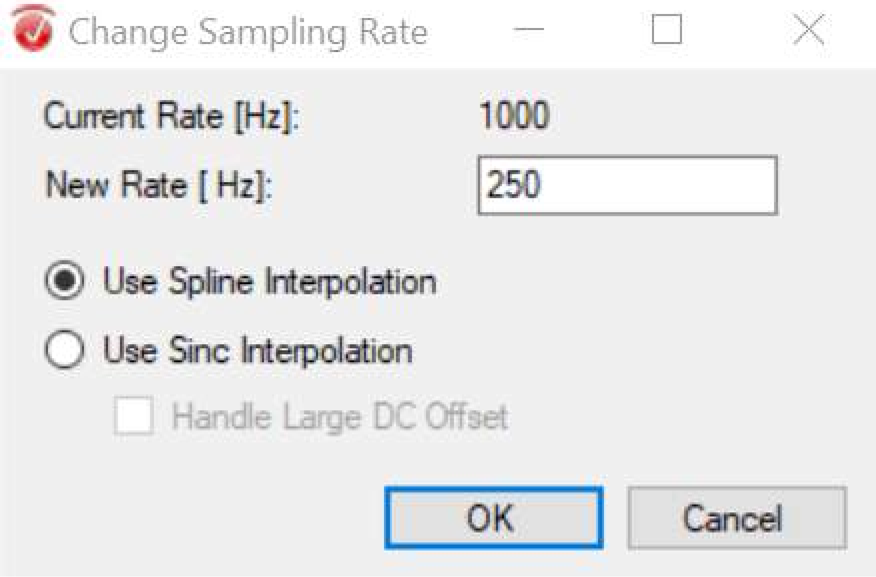

Fig. 30. **Change Sampling Rate** window for EEG data.

Note

The sampling rate differs between EEG and sEMG. Resampling must follow the Nyquist rule, which states the sampling frequency must be at least twice the highest frequency used for analysis. For ERP studies, sampling at 512 Hz is generally accepted [54] and therefore, our protocol resamples at 250 Hz for EEG. Generally, EEG activity of interest is below 30 Hz (including the beta frequency range) [54].

13 **Data Filtering**

Note

Filtering is applied to remove unwanted electrical noise, artifacts, and undesired frequencies. This must be done before segmenting the data [55].

13.1 Go to the **Transformations** tab, click **Data Filtering**, and select **IIR Filters**.

13.2 Enable the **Low Cutoff**, at a frequency of 1 Hz. Then enable the **High Cutoff** at 40 Hz and select **Order** 4 for both. This means that everything below 1 and above 40 is suppressed (see figure 31).

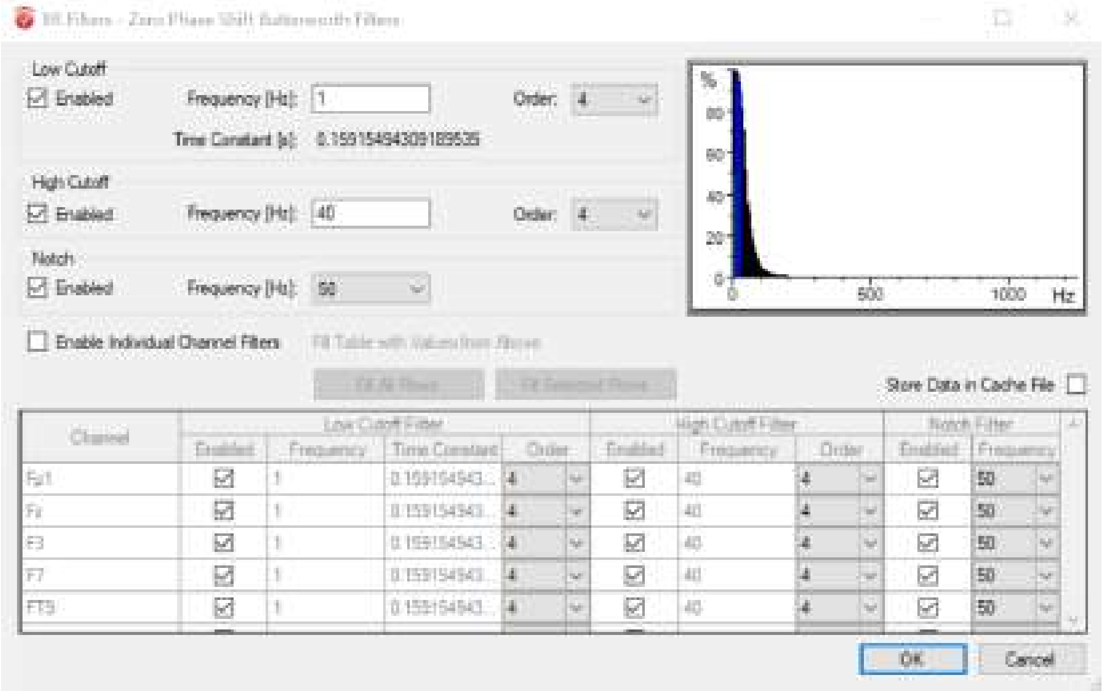

Fig. 31. **IIR Filters** window with recommended EEG data filter settings.

13.3 Enable the **Notch** filter and select 50 Hz as the frequency. The notch filter is adjusted according to national standards. In Europe, the standard is 50 Hz, whilst in the United States it is 60 Hz [47].

Note

To inspect the data filtering, overlay the filtered data onto the previous node ("Edit Channels") for comparison. Select the Edit Channels node and drag the "Filters" node onto the unfiltered data. The filtered data appears in red (see figure 32).

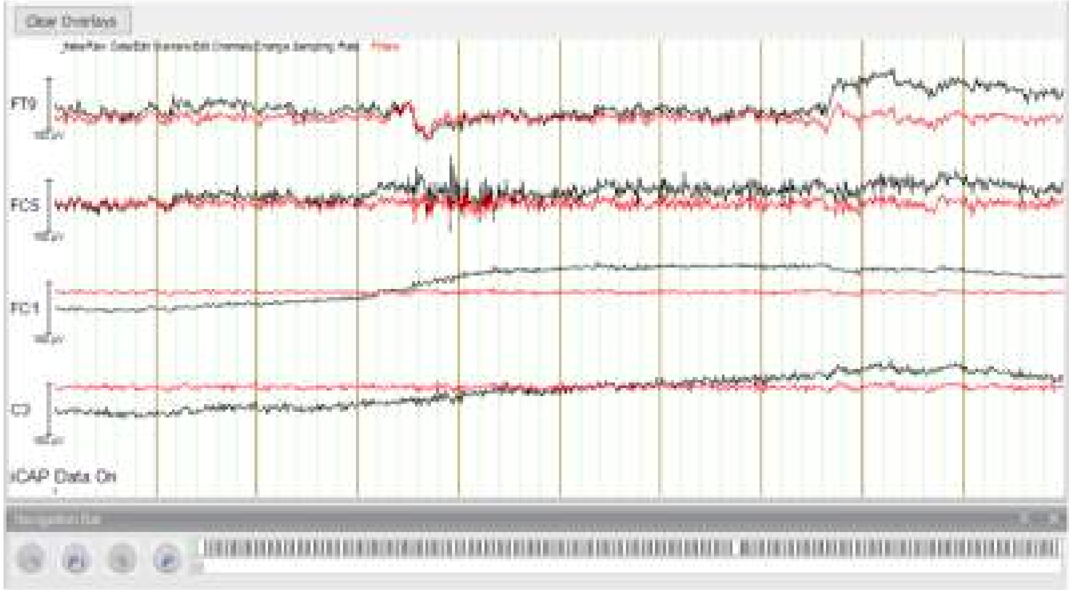

Fig. 32. Filtered data overlays previous **Edit Channels** node.

13.4 Select **Clear Overlays** to return to normal view.

14 **Re-Reference**

Note

Referencing is an important choice, determined by what is being analyzed. Currently, over 10 references are used within EEG studies [56]. Some common references include; average reference (AR), linked-mastoids/ears reference [56], and the predefined reference channel (FCz) [25]. However, in this protocol, we apply the Reference Electrode Standardization Technique (REST) [57]. REST works by referencing the signal to a theoretical neutral point of reference in infinity [25,57].

14.1 Before applying the REST reference to the EEG data, download the open-access plug-in version of the REST tool [57,58], found in Dong and colleagues [58].

14.2 Once downloaded, unzip the compressed file and create a folder in the **C drive**. Ensure all decompressed files are added to this folder.

14.3 In **BrainVision Analyzer software (version 2.2.1, Brain Products GbmH, Gilching, Germany)** [8], select **New Reference** from the **Dataset Preprocessing** tab under **Transformations**.

14.4 First, perform an average reference and include the online reference channel (**FCz**). Move all channels into the **Selected Channels** column and select **Include Implicit Reference into**

Calculation of the New Reference.

14.5 Repeat the process in Step 14.4 on the next page to ensure all channels will have the new reference applied by adding them to the right-hand column, **Selected Channels**.

14.6 Select **Reuse Old Reference Channel**, and name the channel **FCz**.

14.7 Input a name for the reference node, i.e. "Average" or “AVG”.

14.8 Navigate to the **History Template** tab and select **Open**.

14.9 Drag the “restref.ehtp” file in the operation window directly into the new reference.

14.10 Press **File**, then **Load Electrode File** on the pop-up window (this only appears the first time the REST reference is performed).

14.11 Select the “newchan.txt” file from the folder just created in the **C drive**, and select **Calculate Lead File**.

14.12 Once completed, close the window. A new node should appear called “restrefer", this represents the REST reference.

15 **Manual Raw Data Inspection**

Note

Manual data inspection is performed to check for large artifacts that are irregular, both in time and pattern. Ocular (blinks or localized eye movements), muscular (neck and shoulder tension), and cardiac (heartbeats) artifacts [59] are usually not included here as they occur with some regularity. Irregular artifacts must be removed before the Independent Component Analysis (ICA) inspection, as ICA is not suitable for detecting every kind of artifact. It is important to note that repeating, patterned artifacts should be corrected with an ICA to reconstruct the signals without the artifacts, rather than complete removal of a section. For more information on ICA, see Note in Step 16.

15.1 Navigate to **Raw Data Inspection** under the **Transformation** tab and select **Manual**.

15.2 Select and remove sections with large, irregular artifacts (see figure 33). Refer to figure 34 for some examples of common EEG artifacts [59].

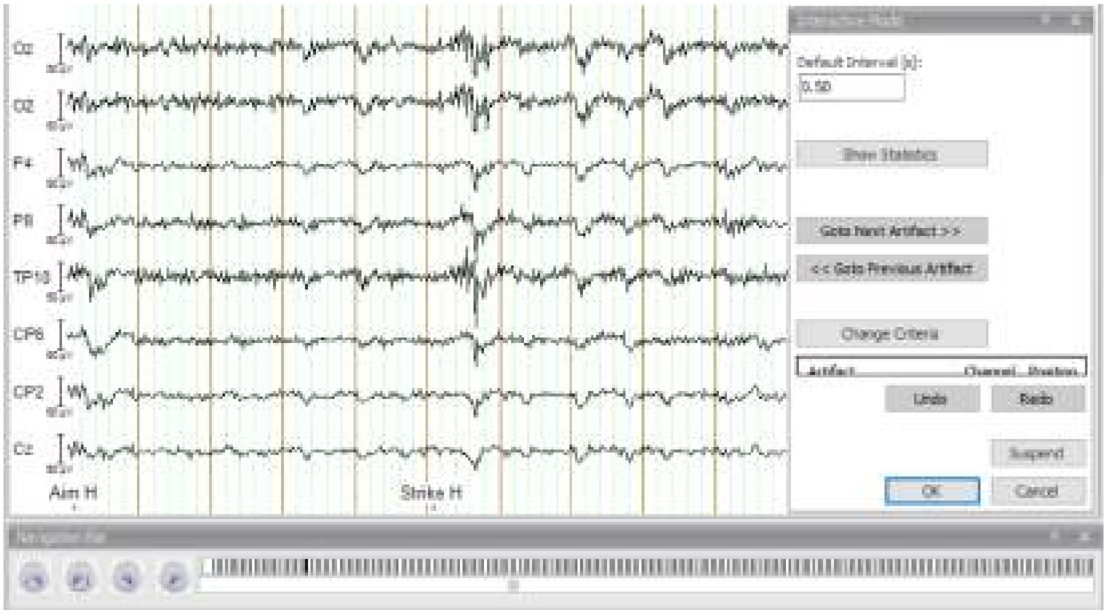

Fig. 33. **Manual Raw Data Inspection Interactive Mode**.

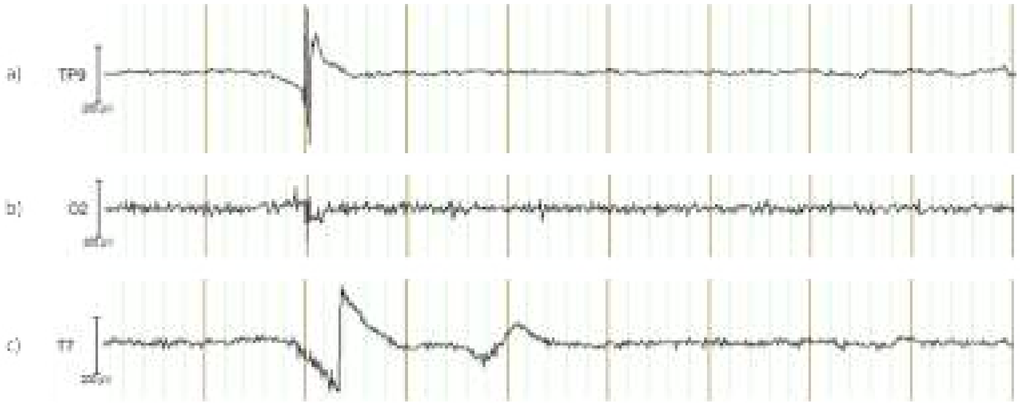

Fig. 34. Some examples of irregular artifacts are muscular artifacts: muscle contraction of the jaw and face (a); continuous neck tension (b); and an electrode pop (c).

16 ICA and Inverse ICA

Note

In general, **ICA** identifies recurring components of data. In EEG, this step is generally used to correct ocular artifacts such as, eye blinks, and muscular artifacts, such as eye movements [59,60]. This analysis must be applied to filtered data.

16.1 Select **ICA** under the **Frequency and Component Analysis** in the **Transformation** tab.

16.2 Uncheck all boxes. Check the **Write to Export Directory** box.

16.3 Select **Enable All** and **Number of Enabled Channels**.

16.4 In the new pop-up window, select **Whole data**.

16.5 In the last window select **Classic PCA, Infomax, Restricted**, **and Energy**. Uncheck **Semiautomatic Mode**.

> **Note**
>
> After **ICA** is complete, the window displays individual components extracted from the EEG data [61].

16.6 Select **Inverse ICA** under the **Frequency and Component Analysis** in the **Transformation** tab.

16.7 Select **Semiautomatic Mode**.

16.8 Adjust the amplitude settings to enable optimal viewing.

16.9 Select **ICA Components** in the **Interactive Mode** display setting.

16.10 Search through the data for deflections [62,63] that represent eye blinks, repeated muscular artifacts, or other repeated artifacts (see figure 35).

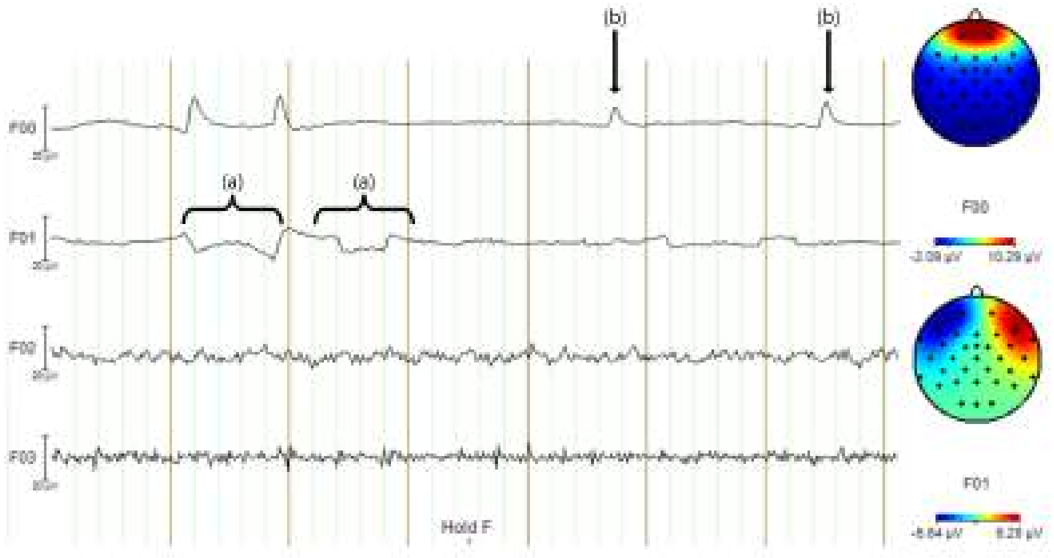

Fig. 35. Example of voluntary horizontal saccades (eye movements) (a) and blinks (b) [61]. Components **F00** and **F01** display negative deflections through segments 2 – 7. The topographic maps correlating to these channels display activity in the frontal region, which represents the channels affected by ocular artifacts.

16.11 Double-click on the component column to remove it. This turns the component box red. Avoid too much data loss by only including components that clearly remove noise (e.g. heartbeat, ocular artifacts) [59–63].

17 **Semi-Automatic Raw Data Inspection**

Note

Semi-automatic data inspection can now be performed to remove artifacts that could not be removed during ICA.

17.1 Navigate to **Raw Data Inspection** under the **Transformation** tab and select **Semi-Automatic**.

Note

Semi-automatic mode allows for manual inspection and editing but highlights data suggested for removal, based on settings adjusted according to the experiment.

17.2 On each tab, adjust the following settings, according to your experiment. This protocol outlines the following recommended settings:

- ***Gradient***:
  – Maximal allowed voltage step: 10 µV/ms
  – Mark as Bad: Before Event: 200 ms / After Event: 200 ms
- ***Max-Min***:
  – Maximal allowed difference of values in intervals: 100 µV

– *Interval Length: 200 ms*
– *Mark as Bad: Before Event: 200 ms / After Event: 200 ms*

- ***Amplitude****:*
  – *Minimal allowed amplitude: -60 µV*
  – *Maximal allowed amplitude: 60 µV*
  – *Mark as Bad: Before Event: 200 ms / After Event: 200 ms*
- ***Low Activity****:*
  – *Lowest allowed activity in intervals: 0.5 µV*
  – *Interval Length: 100 ms*
  – *Mark as Bad: Before Event: 200 ms / After Event: 200 ms*

17.3 Carefully work through the dataset, removing all sections that contain artifacts, as before in Step 15.

18 **Topographic Interpolation**

Note

Topographic interpolation is performed when a channel is contaminated by artifacts. If a channel was removed at the start of the process, due to noise, signal loss, bridging, or anything else, they can be replaced by other clean channels with interpolation. However, this interpolation only represents an estimated signal for the noisy channel and should be interpreted with caution [59].

18.1 Select **Topographic Interpolation** in the **Transformation** tab. Select **Interpolation by Spherical Splines**, check **Default Lambda (1e-5)**, and **Keep Old Channels**.

18.2 Click on **Select from Map**.

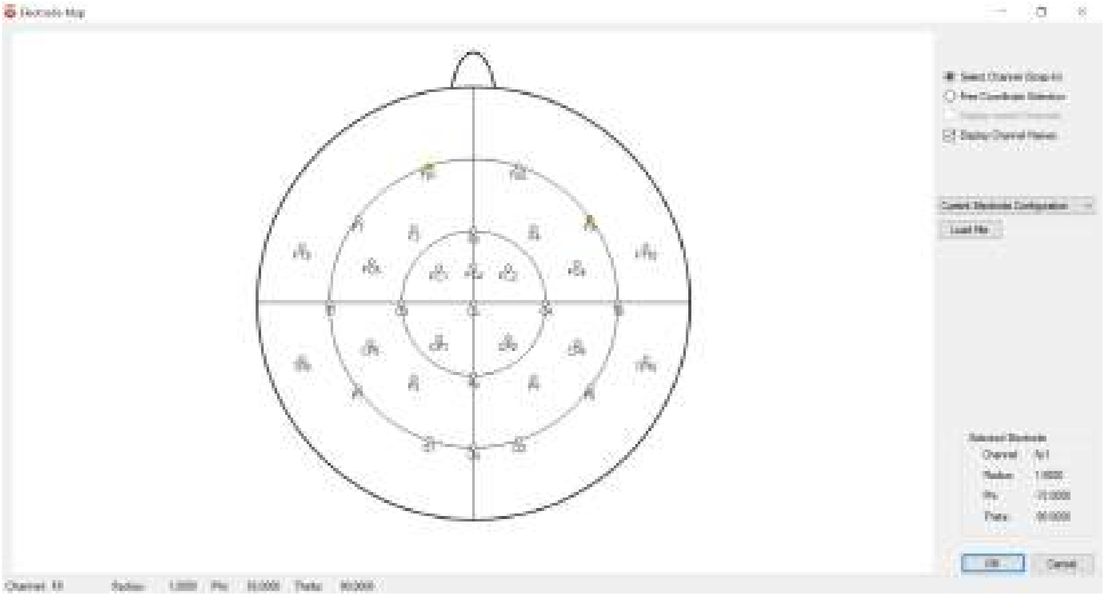

Fig. 36. **Electrode Map** window. Channels previously removed due to signal loss or noise are selected for interpolation using surrounding channels.

18.3 Ensure **Select Channels** and **Display Channels Names** are checked and select the configuration used in the experiment from the drop-down menu. For this protocol, we used a 32-channel 10-20 configuration [1].

18.4 Select the channel(s) removed on the map, and then select **OK** (see figure 36).

19 **Segmentation**

Note

Segmentation is the subdivision of the data into different segments or epochs. **Segmentation** can be performed according to **Predefined Annotations/Markers**. This step extracts every repetition of each stage of the experiment from the recording.

Note

In this study, segments are taken prior to the start of the task/marker to perform a baseline correction on the data (see figure 37). Segments will start 200 ms before the marker, and 15000 ms after the marker (the duration of the complete task, including all three stages, excluding "Rest"). The total length of the segment will be 17000 ms. This varies according to the length of the task and the information important for the study.

19.1 In the **Transformations** tab, under **Segment Analysis Functions** click **Segmentation**. In the pop-up window select **Create new Segments based on a marker position** and **Cache data to a permanent file**. Check **Cache Data on Requested**.

19.2 Select the marker of interest (e.g. “Hold”, “Aim”, and “Execute”) from the **Available Markers** and add them to the column on the right, **Selected Markers**.

19.3 Select **Based on Time** and insert the new time of the segment. If the segment should include data before the marker placement, the **Start** box should begin with a minus sign (–), e.g. "–200 ms".

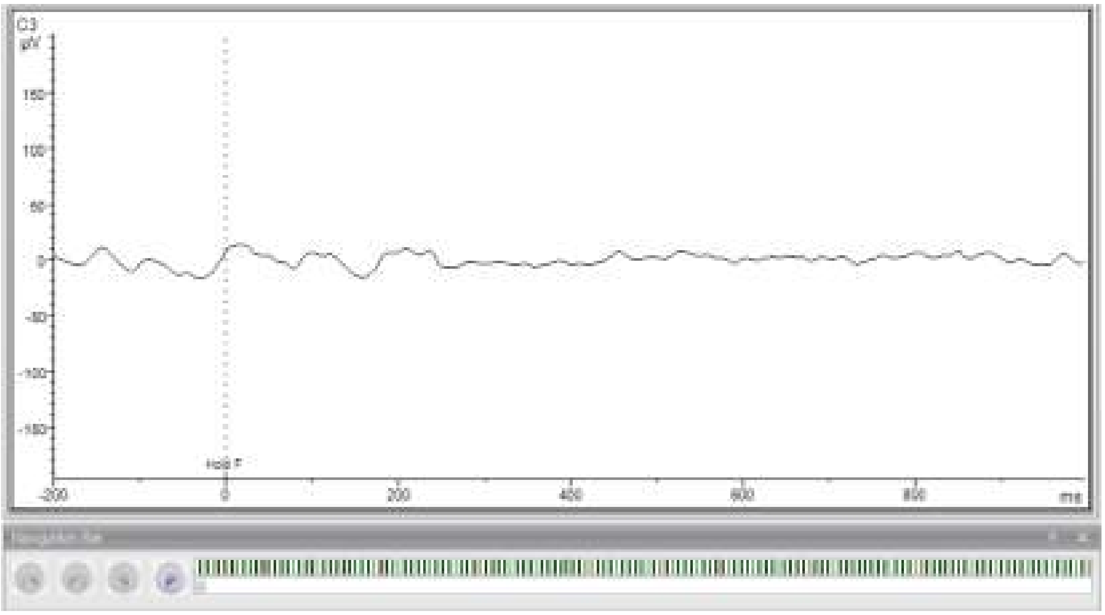

Fig. 37. **Segmentation** of the "Hold" stage of the flake-cutting task. Epoch ranges from –200 ms to 1000 ms. 0 ms represents the trigger onset.

20 **Baseline correction**

Note

Temporal drifts distinct from the experiment often occur in EEG data due to various internal and external sources changing over time [64]. To reduce the effect of these interferences a baseline correction can be applied to the data [64,65]. The baseline should be taken from a period before or after the trigger. This value is then subtracted across the EEG data [66]. Baseline correction is necessary for event-related potential studies [64], however, in other cases, a baseline correction may not be required, depending on the nature of the data and analysis being conducted.

20.1 In the **Transformations** tab, under **Segment Analysis Functions** click **Baseline Correction**.

20.2 Type "–200 ms" in the **Begin[ms]** and "0 ms" in the **End[ms]** box. This ensures the baseline correction is calculated over data before the trigger.

21 **Condition Segmentation**

Note

Condition segmentation further segments the data based on the different conditions of the study, i.e. “Hold”, “Aim”, and "Execute". Each marker is now processed separately.

21.1 Under **Segment Analysis Functions** select **Segmentation**. Select **Create new Segments based on marker positions**.

21.2 Select **Based on Time** and insert the final segmentation of the EEG steps. In this study, the markers were placed at "0" in the **Start [ms]** box and "1000" in the **End [ms]** box. Ensure the **Allow Overlapped Segments** box is unchecked.

21.3 Each node should only contain one step, i.e. “Hold”.

21.4 Rename the **Segmentation** node to something memorable, such as; “Segmentation_Hold”.

21.5 Repeat Steps 21.1 to 21.4 for the other markers of interest, i.e. “Aim” and “Execute”.

22 **Artifact Rejection**

Note

Artifacts are an inevitability during EEG recording. The removal of large artifacts is important to ensure proper processing of the EEG data, however, there is currently no technique that can effectively remove all artifacts, without risking the removal of real EEG signal [21,59,67]. These artifacts are often not visible during ICA, as they do not appear as repeated patterns that can be detected in the software. When these artifacts occur in EEG data, it is important to exclude the areas or participants affected to avoid inaccurate results (see figure 34 for some common examples of irregular artifacts) from statistical analyses [5].

Note

During stone tool motor tasks, such as flake cutting, it is important to take several steps to ensure minimal interference. These include good impedance, reducing unnecessary movements, i.e. side-to-side head movement or excessive upper arm motion, whilst still allowing for a natural range of motion. Particular care should be given to cleaning channels close to areas known to cause muscular tension, for example, in the mastoid region [59].

22.1 In the segmentation node, select **Artifact Rejection** in the **Transformations** tab, under **Artifact Rejection/Reduction**.

22.2 Under **Inspection Method** select **Semiautomatic Segment Selection**.

22.3 In **Channels** click **Enable All**.

22.4 In the **Criteria** pop-up window, input criteria according to your experiment. In this protocol, the following settings are recommended:

- ***Gradient***:
  - *– Maximal allowed voltage step: 50 µV/ms*
  - *– Mark as Bad – Before Event: 200 ms / After Event: 200 ms*
- ***Max-Min****: Leave "Check maximal difference of values in intervals" unchecked.*
- ***Amplitude***:
  - *– Minimal allowed amplitude: -100 µV*
  - *– Maximal allowed amplitude: 100 µV*
  - *– Mark as Bad – Before Event: 200 ms / After Event 200 ms*
- ***Low Activity****: Leave "Check low activity in intervals" unchecked.*
- ***Intervals****: Leave "Limit the check to an interval within segment" unchecked*

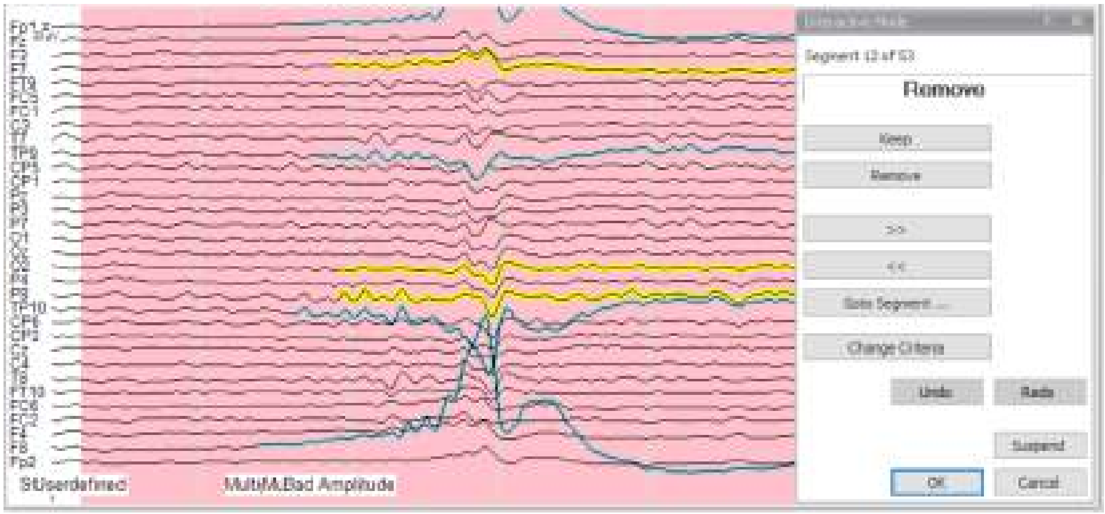

Fig. 38. **Artifact Rejection Semiautomatic Interactive Mode**. Segments outside the criteria set are highlighted for removal.

22.5 Repeat Step 22 for all other segmented nodes.

22.6 Evaluate the data to ensure that either the number of segments suggested for removal is under 10% of the total data set, or 40 repetitions remain in the data set [68] (see figure 38). To find this information, navigate to **Operating Infos** by right-clicking on the **Artifact**

Rejection node. Refer to the **Number of kept segments** and the **Number of removed segments**.

23 **Fast Fourier Transformation (FFT)**

Note

Fast Fourier transformation (FFT) allows us to extract frequency content from an EEG signal. The power spectrum (μV²) is composed of multiple frequencies displaying various amplitude peaks and troughs, which the FFT can use to extract the frequency bands contained within the EEG spectrum [69,70]. For our analysis, we extracted a segment of the EEG signal we wanted to analyze (in this case 0 – 1000 ms) and performed FFT on each individual and repetition, then averaged the FFT (Step 24). The output is the power (μV²) at the selected frequency band. For the active stone tool-cutting task, we are interested in beta (12.5 – 30 Hz) [71].

23.1 In the **Transformations** tab, navigate to **Frequency and Component Analysis** and select **FFT**.

23.2 Ensure the output is set to **Power [μV²]**. In this protocol, we use a 10% Hanning (Hann) window, select **Hanning Window**, and type "10" in **Window Length [%]** under **Parameters** to reduce spectral leakage resulting in improved frequency resolution.

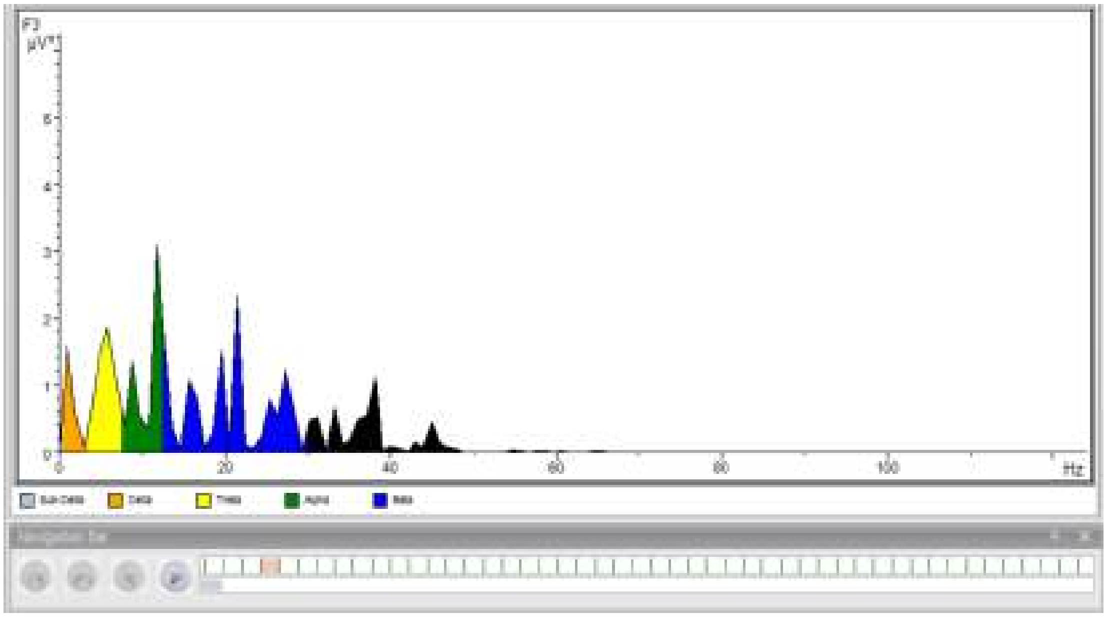

Fig. 39. **FFT** output of an individual's single trial (before averaging). Channel **F3** extracted into frequency bands. Delta (orange); theta (yellow); alpha (green); beta (blue); gamma (black).

23.3 Once performed, the **FFT** extracts the power (μV²) at each frequency band in each segment (see figure 39).

Note

In **BrainVision Analyzer software (version 2.2.1, Brain Products GbmH, Gilching, Germany)** [8], FFT can be used to visualize the EEG spectrum. FFT enables the extraction of frequency bands of interest for statistical analysis and can be used to visualize frequency band maps. This is usually performed after grand averaging (see Part 5).

24 **Average**

Note

Averaging is commonly used to enhance the signal-to-noise ratio. When analyzing power in any frequency band, averaging helps highlight consistent changes in power associated with the task while reducing random noise [72–74]. As each stage/segment ("Hold, "Aim", and "Execute") is time-locked, averaging calculates the arithmetic mean of all segment repetitions in the frequency domain [72–74]. Averaging occurs on the extracted power values in the frequency domain suppressing noise or artifacts that vary across the segments [72–74].

24.1 Navigate to **Average** in **Segment Analysis Functions**, under the **Transformations** tab.

24.2 Select **Full Segment Range**. Averaging should be performed on all segments.

24.3 Repeat this process for the other segments (markers) of interest.

25 **Data Visualization**

STEP CASE

Electroencephalography 14 steps

26 **Grand Averaging**

Note

To best capture the EEG signal in the frequency domain, the grand average can be used to create a band map that includes the average power signal (μV²) of all participants and visually demonstrates the overall cognitive activation occurring in each task.

26.1 To create the grand average for each experimental stage, navigate to **Segment Function Analysis** in the **Transformation** tab, select **Result Evaluation**, and then **Grand Average**.

Note

The grand average is the average of all participants' averaged segments.

26.2 In the pop-up window, add all **History Nodes** to be calculated in the grand average. In this case, ensure all history nodes of participants are correctly labeled, i.e. "Average_Flake_Hold", "Average_Flake_Aim", and "Average_Flake_Execute" (see figure 42).

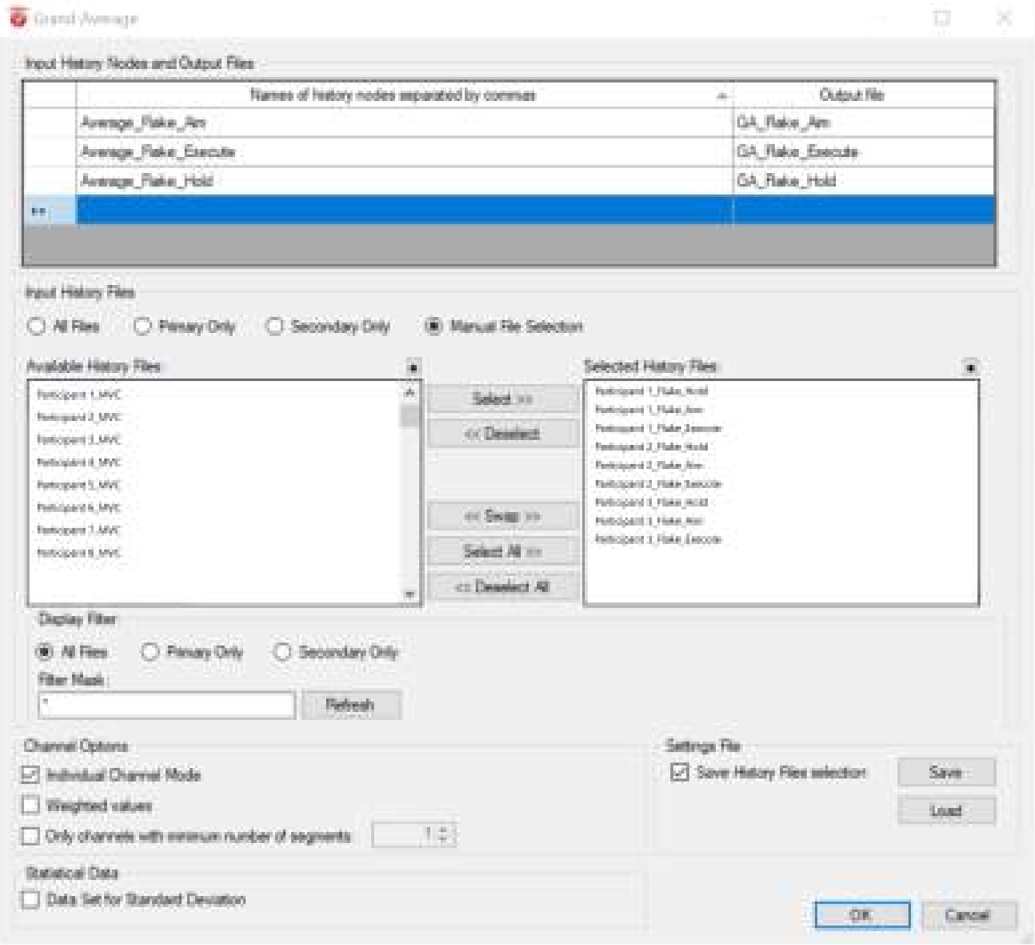

Fig. 42. **Grand Average** window. Creation of the grand average for the three flake cutting stages; "Hold", "Aim", and "Execute".

Note

Ensure all nodes are labeled correctly for each participant, i.e. "Average_Flake_Hold".

26.3 Rename each **Output file**, i.e. “Grand_Average_Flake_Hold”.

26.4 Add all participants' history files into **Selected History Files**.

26.5 Check the **Save History Files selection** and **Individual Channel Mode** boxes.

26.6 The grand averaged data of each experimental stage should now be available in the **Secondary** tab.

27 **Topographic EEG Maps of Spectral Power**

Note

To visualize EEG data effectively, frequency band maps can be generated in **BrainVision Analyzer software (version 2.2.1, Brain Products GbmH, Gilching, Germany)** [8]. These maps display the spatial distribution of power (μV²) across different frequency bands, such as beta, providing insights into participants’ cognitive and motor-related activation levels during the experiment. In this study, the beta frequency band was of particular interest, as variation in beta power is often associated with heightened cognitive and motor-related activity [77]. These band maps reveal varying power (μV²) within the beta frequency. Notably, in the motor cortex, a phenomenon known as beta-desynchronization can occur during voluntary movements, indicating increased motor activation despite a reduction in beta power [78–82]. This pattern, documented extensively in EEG studies, is a well-known response in motor-related tasks [78,79,83]."

27.1 Select the **Raw Data** node of the grand average file, i.e. “Grand_Average_Flake_Hold”, in the **Secondary** tab.

27.2 Right-click on one of the channels, select **Switch View**, then choose **Band Mapping View** (see figure 43).

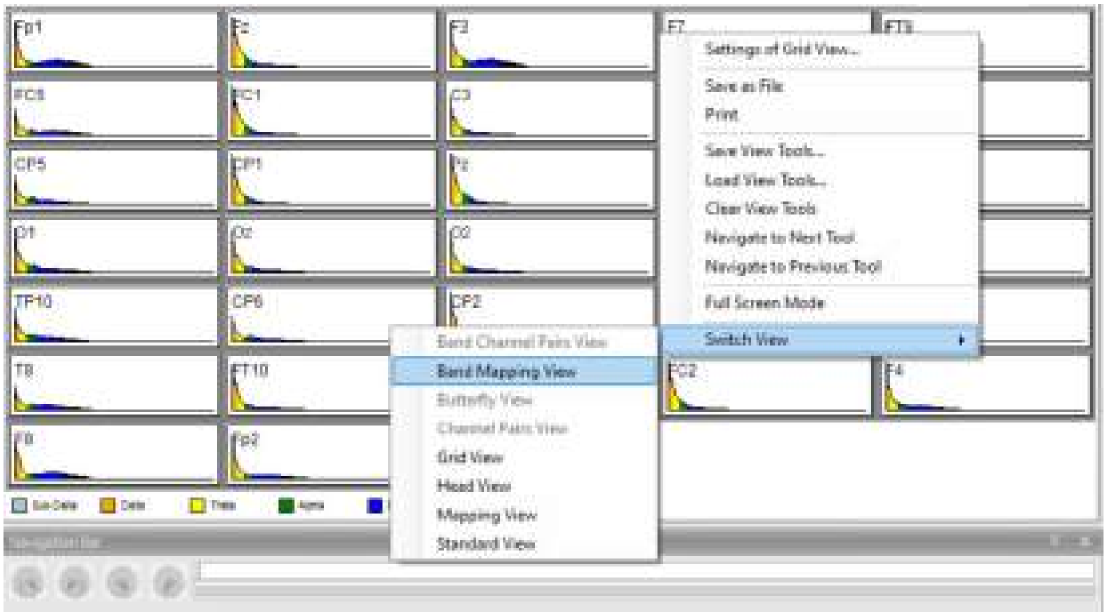

Fig. 43. Pathway to Frequency Band Mapping View from Grand Averaged FFT data.

27.3 To capture all channels, and the frequency bands of interest, right-click onto the band map and select **Settings of Band Mapping View**.

27.4 In **Settings**, ensure **Scaling** is set to **Manual Scaling** which enables you to manually adjust the scale under the band map, as desired. For this task, **Minimum [µV]:** is set to "0.1" μV^2 and **Maximum [µV]:** is set at "0.6" μV^2 for beta power. This can be customized to optimize the display for your experiment. However, the minimum value should be above 0, as these are power values. The **Direction** should display the brain regions of interest, in this case, the **Top** view is chosen. To enable viewing of all EEG channels on one band map, select **All In One Map** (see figure 44).

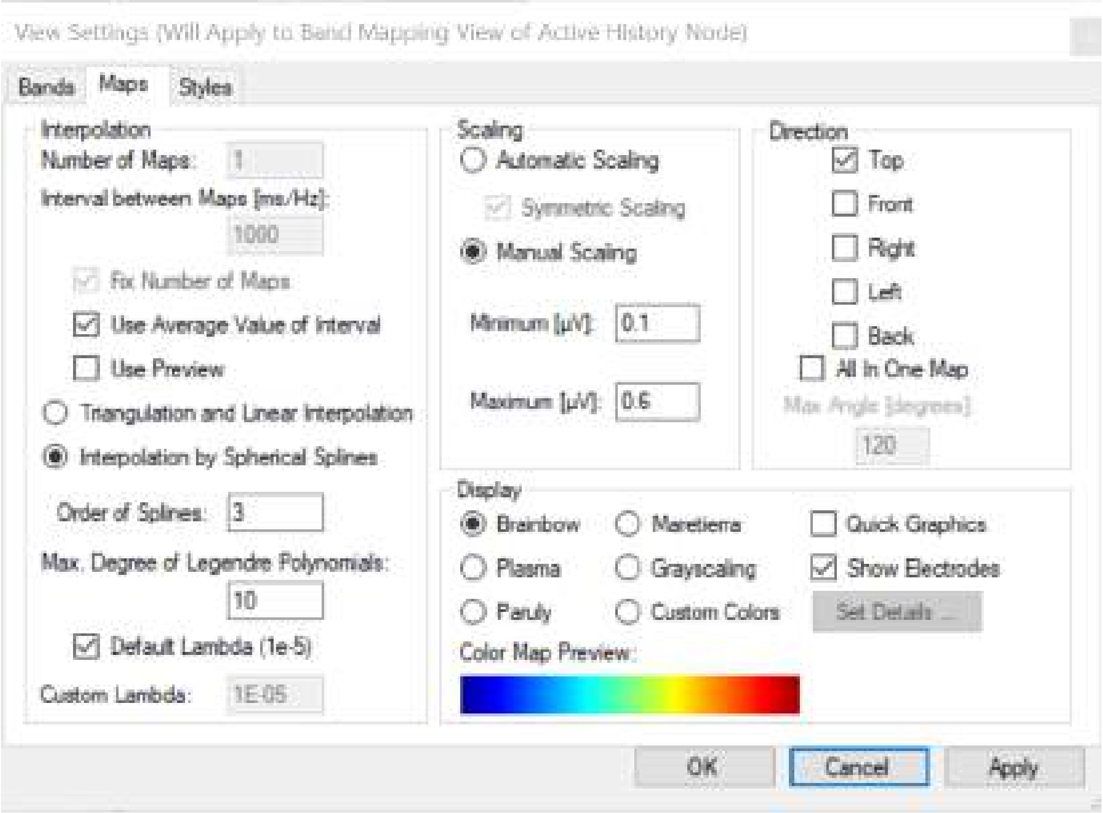

Fig. 44. **Band Mapping View Settings** for the "Aim" stage, **Maps** tab.

27.5 On the **Bands** tab, delete all frequency bands that are not of interest in your study. We only require **Beta** in this study. Finally, select **Apply** (see figure 45).

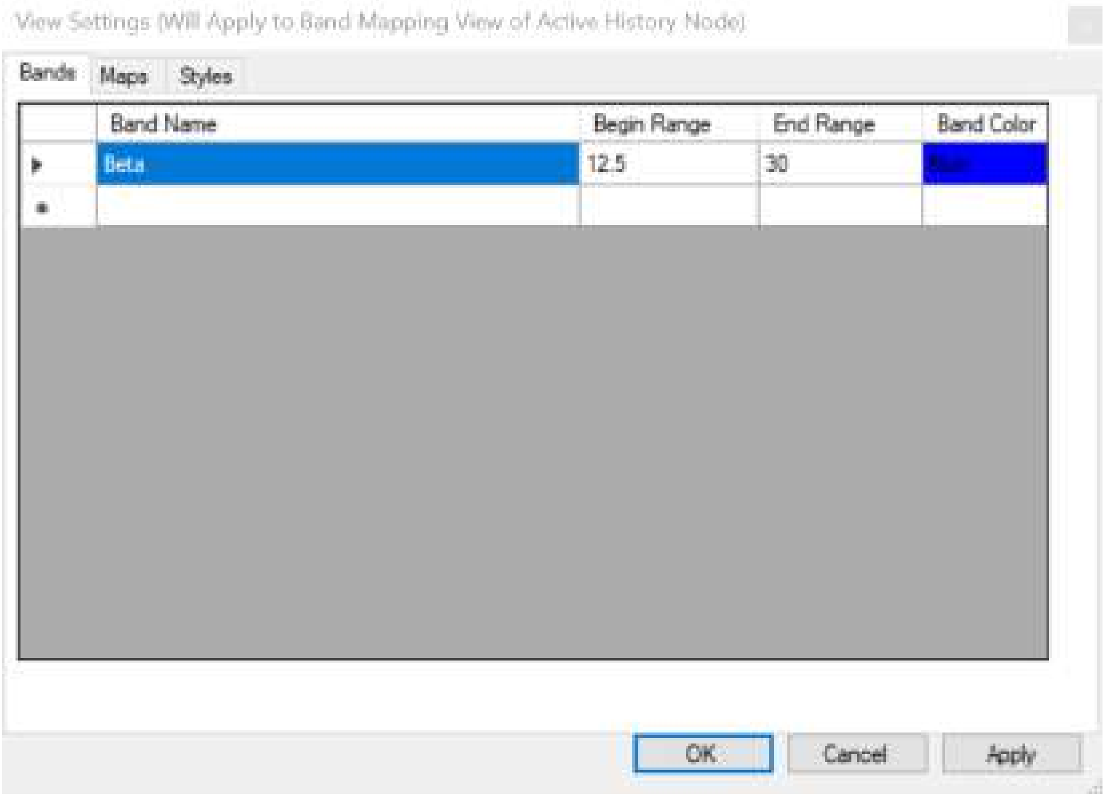

Fig. 45. **Band Mapping View Settings** for the "Aim" stage, **Bands** tab.

27.6 Right-click the band map and select **Save as File** to export as an image file (see figure 46). Save the band map on your computer as an image file by changing the file type to one of the following; .png, .jpeg, .gif, or .tif.

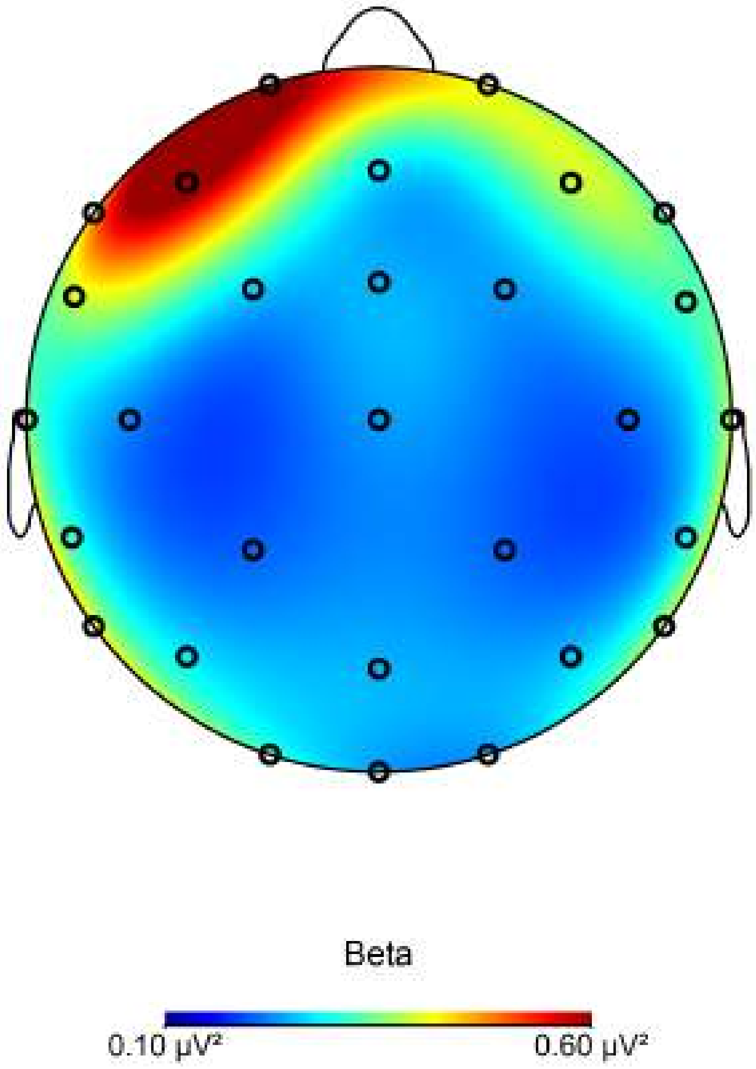

Fig. 46. Example of a grand averaged FFT power (μV²) band map of the "Aim" stage (0 – 1000 ms). Beta (12.5 – 30 Hz) exhibits a decrease in power in the motor cortex and an increase in the frontal region.

Note

During stone tool use tasks, muscular artifacts are often unavoidable in the EEG data, despite the above-mentioned rigorous artifact-cleaning processes. These artifacts frequently appear in channels around the mastoids, often due to neck and shoulder tension, jaw clenching, and the carotid artery, known as the pulse artifact [59]. To mitigate this, it is essential to follow guidelines that minimize facial and upper body movement during recording and to conduct a thorough manual inspection of the raw data.

STEP CASE

surface Electromyography 3 steps

26 **Principal Component Analyses (PCA)**

Note

Principal Component Analysis (PCA) is an effective method for visualizing sEMG data of multiple muscles (sEMG variables) simultaneously, allowing for the observation of variation in muscle coordination patterns across different tool-using tasks and stages (i.e., Flake Hold, Flake Aim, and Flake Execute). In this study, as well as in our previous research [12], the PCA was performed using the software package **PAST, version 4.03** [52]. To account for inter-participant variation in muscle force-producing capacity, the PCA was conducted on the calculated %MVC values and relied on the correlation matrix, to prevent variables with larger variances from disproportionately influencing the principal components [52,75]. The broken-stick model is used to determine the number of principal components (PCs) to retain for plotting and further statistical analysis. Factor loadings are used to interpret the multivariate patterns represented by each component axis [76]. Relevant PC scores are then extracted, which can be used in further statistical analyses, such as assessing significant differences between stone tool stages (Hold, Aim, Execute) using paired t-tests or other approaches (e.g., see [12]). Finally, combining sEMG PCA plots with EEG frequency band maps enabled the simultaneous visualization of both muscular activation levels and cognitive activation patterns associated with each stage.

26.1 Open **PAST (version 4.03)** [52] and format the data, color labeled by task step (see figure 40). Navigate to **Multivariate** and select **Principal Components (PCA)** under **Ordination**.

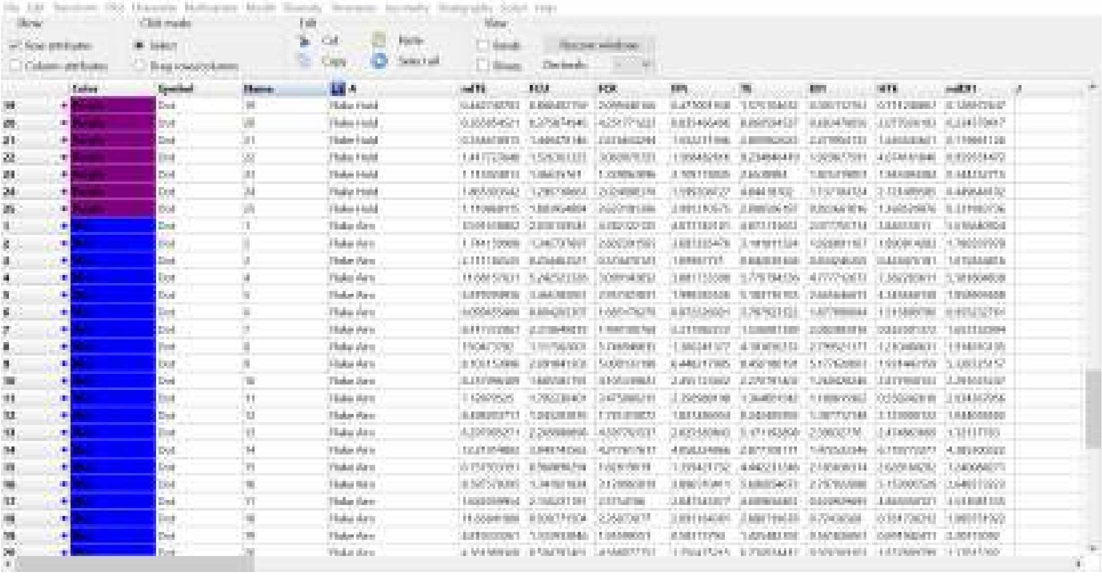

Fig. 40. Formatted, color-coded sEMG %MVC data in **PAST (version 4.03)** [52].

26.2 Select **Correlation** under **Matrix**, to standardize the variables and avoid biasing. Select **Disregard** under **Groups** so no assumptions are made between task steps (see figure 41). The **Scatter plot** tab visually displays the data points in a two-dimensional space. Select **Convex hulls** to group each stage (i.e. Flake Hold), and **Biplot** to display each %MVC loading on the visible components. Select **Broken Stick** in the **Scree plot** tab to determine how many

components represent the majority of the data variance. The **Scores** tab provides the **PC Scores** which can be used for further statistical analyses.

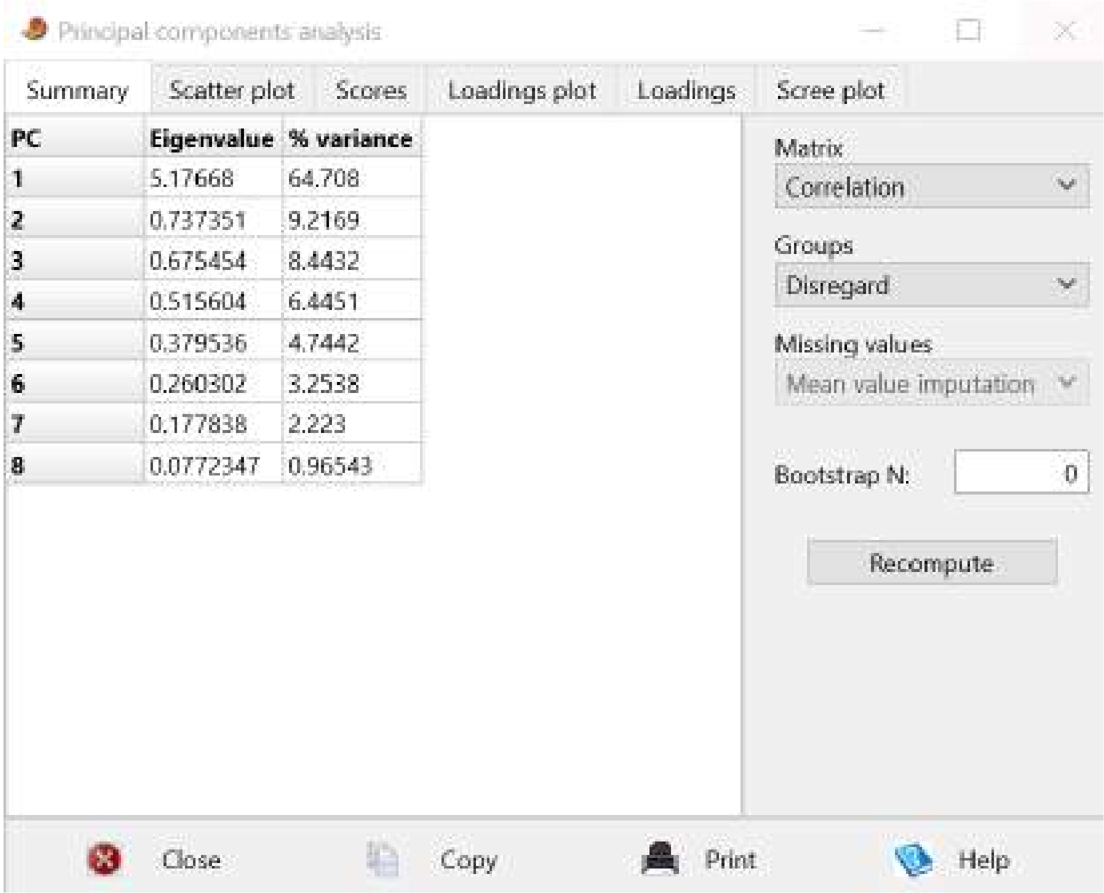

Fig. 41. Principal component analysis summary window in **PAST (version 4.03)** [52] displaying recommended settings for stone tool cutting task PCA.

STEP CASE

surface Electromyography

40 steps

Preprocessing is an essential part of collecting sEMG data for analysis and visualization. The preprocessing steps mentioned in Part 4 are specific to our experimental design and objectives. Below, we provide steps implemented as part of our experimental pipeline, which should serve as a guide for performing your own experiment.

- Edit Channels
- Down Sample (Change Sampling Rate)
- Data Filtering
- Segmentation
- Rectification
- Creating Max Markers
- Averaging
- Exporting
- Calculating Percentage Maximum Voluntary Contraction (%MVC)

11.1 Check the raw data and note all noisy or dead sEMG channels, that may require removal (see figure 11).

- Dead channels appear as a flat line.
- Noisy channels typically display repeating unpatterned, large spikes that are not mirrored in other channels.
- Clipping interference or saturation is when the amplitude of a signal reaches levels beyond the range that can be recorded. This can occur due to high signal amplification or improper electrode attachment to the skin [42].

Note

If channels that are noisy or have a signal deadline only occur during a few repetitions (i.e. >40 repetitions of the task remain useable), these sections can be removed at a later point and the channels can be kept in the dataset.

11.2 Remove noisy or dead channels from the analysis by navigating to **Edit Channels** under the **Transformations** tab. Deselect the relevant channels for removal. For sEMG, all EEG channels should also be removed as preprocessing differs between the two methodologies (see figure 17). Additionally, deselect the acoustic marker channel (labeled "BEEP" here) used to define task beginning and/or end, to ensure only sEMG channels are processed.

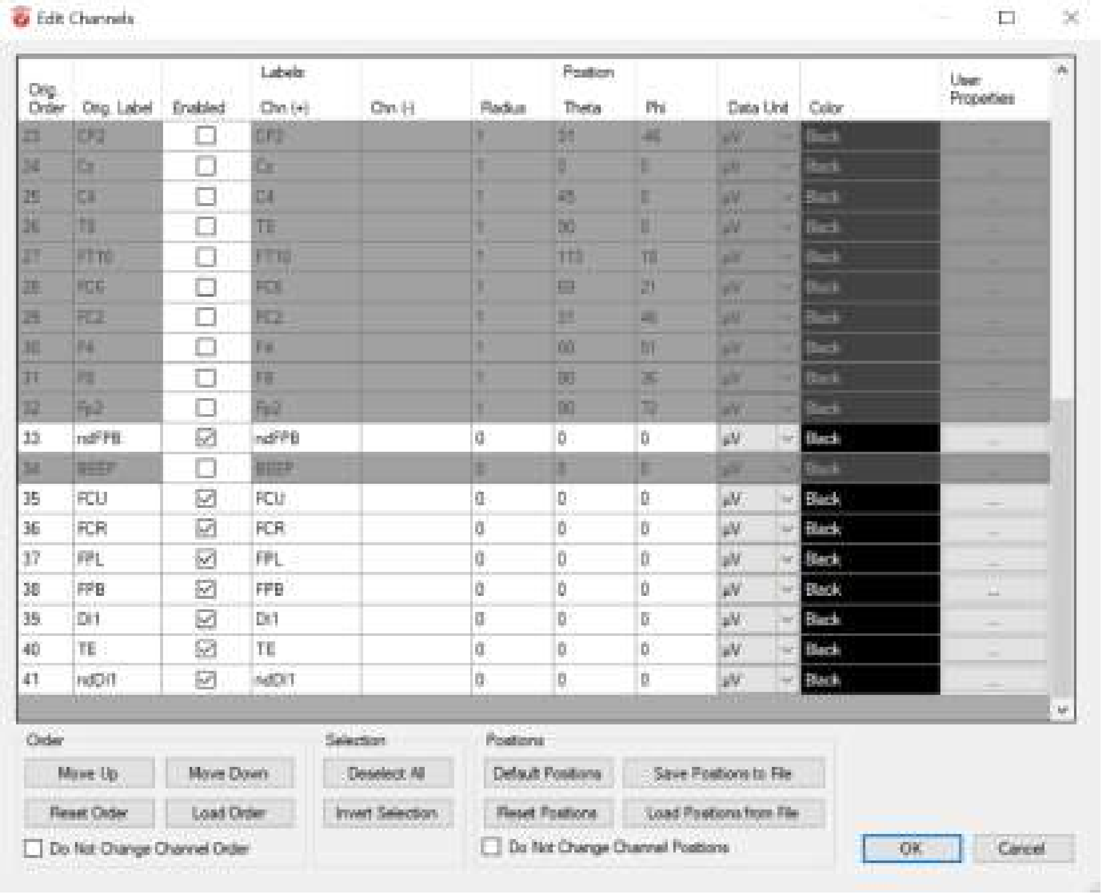

Fig. 17. **Edit Channels** window for sEMG preprocessing. All EEG channels have been deselected.

12 Down Sample (Change Sampling Rate)

12.1 Navigate to the **Transformations** tab and click **Change Sampling Rate**. The current sampling rate is shown under **Current Rate**. Enter the new sampling rate in **New Rate** and select **Spline Interpolation** (see figure 18).

Note

Resampling must follow the Nyquist rule, which outlines that the sampling frequency must be at least twice the highest frequency used for analysis. 500 Hz is recommended for the down sample rate, as EMG activity occurs between 5 and 450 Hz [6,7].

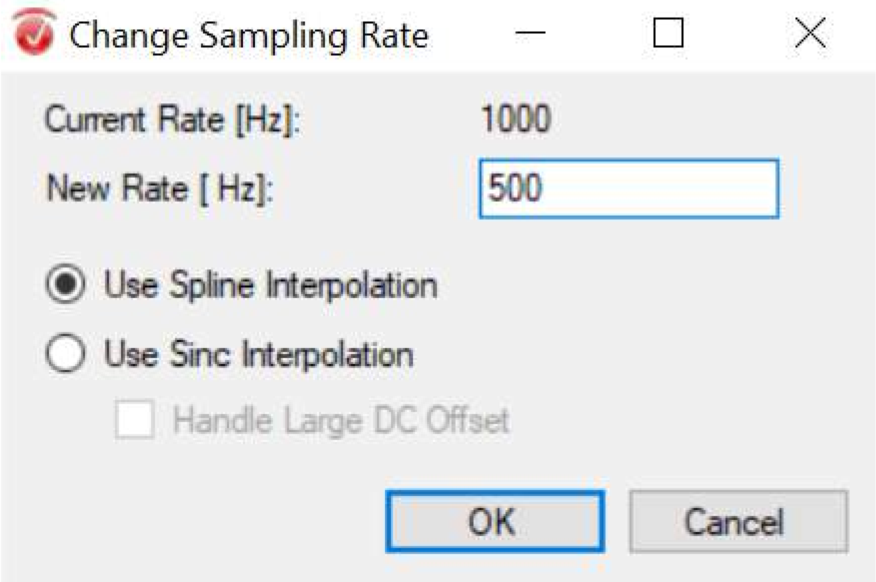

Fig. 18. **Change Sampling Rate** window for sEMG data.

13 **Data Filtering**

Note

Filtering is applied to remove unwanted electrical noise, artifacts, and undesired frequencies. This must be done before segmenting the data [11,43].

13.1 Go to the **Transformations** tab, click **Data Filtering**, and select **IIR Filters**.

13.2 Enable the **Low Cutoff** (high pass filter), at a frequency between 10 – 20 Hz. Then enable the **High Cutoff** (low pass filter) between 500 – 1000 Hz and select **Order** 4 for both [44–46], to conserve the most important frequencies of EMG [11] (see figure 19).

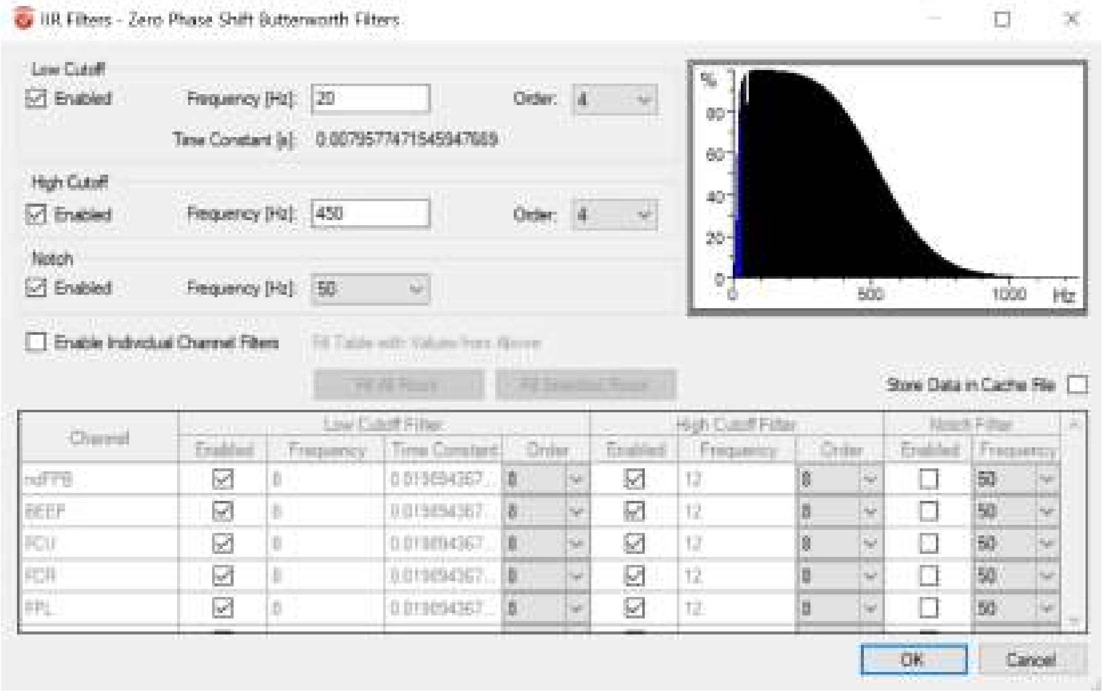

Fig. 19. **IIR Filters** window with recommended EMG data filter settings.

13.3 Enable the **Notch** and select 50 Hz as the frequency. The notch Filter is adjusted according to national standards. In Europe, the standard is 50 Hz, whilst in the United States it is 60 Hz [47].

Note

To inspect the data filtering, overlay the filtered data onto the previous node (**Edit Channels**) for comparison. Select the **Edit Channels** node and drag the **Filters** node onto the unfiltered data. The filtered data appears in red (see figure 20).

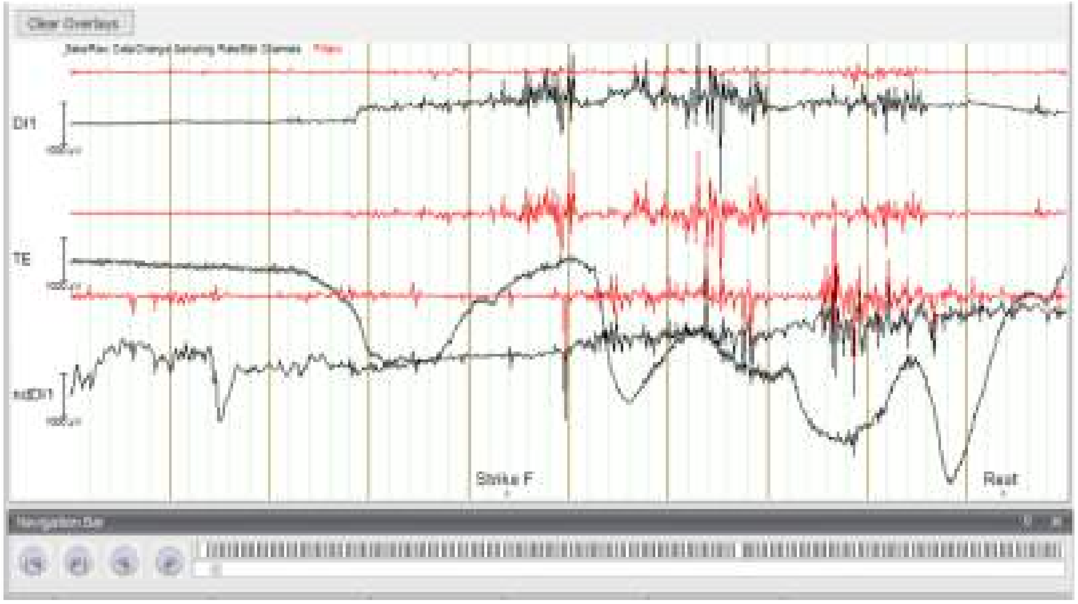

Fig. 20. Filtered data overlays previous **Edit Channels** node.

13.4 Select **Clear Overlays** to return to normal view.

14 Manual Raw Data Inspection (EEG-only)

Note

Manual data inspection should be performed to remove any artifacts or segments where the signal does not show a true muscular EMG signal.

14.1 Navigate to **Raw Data Inspection** under the **Transformation** tab and select **Manual**.

14.2 Select and remove sections contaminated by artifacts, see figure [11] for some common artifact types in sEMG.

15 Segmentation

Note

Segmentation is the subdivision of the data into different segments or epochs. Segmentation can be performed according to **Predefined Annotations/Markers**. This step extracts every repetition of each stage of the experiment from the recording.

15.1 In the **Transformations** tab, under **Segment Analysis Functions** click **Segmentation**. In the pop-up window, select **Create new Segments based on a marker position** and **Cache data to a permanent file**. Select **Cache Data on Requested**.

15.2 Select a marker of interest (e.g. "Aim") from the **Available Markers** and add it to the **Selected Markers** column on the right-hand side.

15.3 Select **Based on Time** and insert the new segment length, e.g. **Start [ms]:** "0" and **End [ms]:** "1000".

Note

In EMG, segments should only capture the muscular activation during a task. EMG data is more easily distinguishable from noise and inactivity than EEG due to the unique EMG signal signature and higher resting amplitude.

Note

Segmentation **Based on Time** is recorded in milliseconds (ms). If muscle activation is observed from task onset, segment 0 ms for the start.

15.4 Repeat this process for the other markers of interest, i.e. "Hold" and "Execute" (see figure 21).

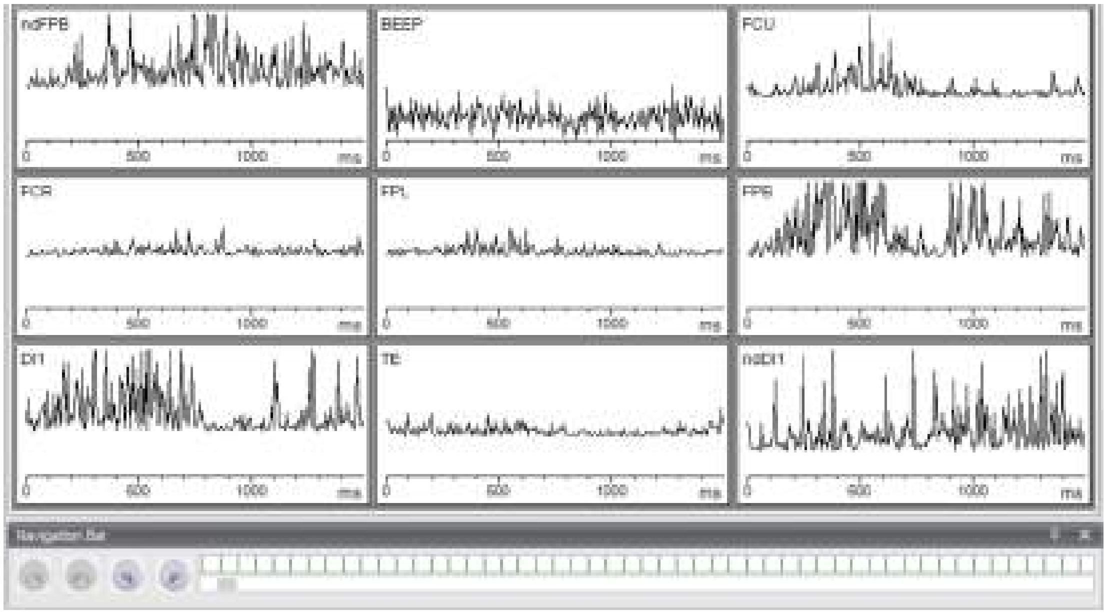

Fig. 21. Segmentation of the stone tool cutting task. Each green segment in the **Navigation Bar** represents a repetition of a stage (i.e. "Aim").

16 Rectification

Note

This protocol outlines one method of EMG signal processing, **Rectification** [11]. **Rectification** is an important step, as the EMG signal naturally has a mean close to zero due to the oscillations (positive and negative) of the recorded muscular signal [48]. By rectifying the data, all values are transformed into positive values, creating more meaningful data (see figure 22) [49,50].

16.1 Navigate to the **Transformation** tab, and select **Rectify**, under **Data Preprocessing**.

16.2 Ensure all channels are enabled, and check **Keep Remaining Channels**.

16.3 Select OK. Channels should now look like an envelope of the original signal (see figure 22).

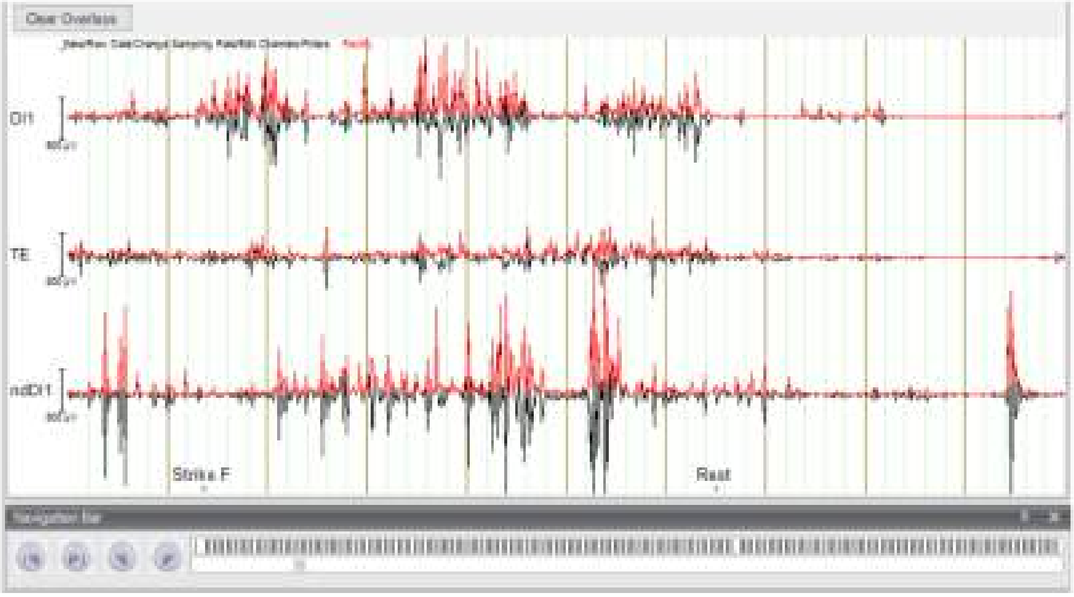

Fig. 22. Rectified data (red) overlaying a previous node (black).

17 **Creating Max Markers**

Note

Max Markers are used to find the MVC peaks during the dynamometer task. These peaks determine the benchmark used to create the %MVC values of the experimental tasks. Max Markers are only calculated for the dynamometer MVC tasks.

17.1 Select the MVC dynamometer task segment node.

17.2 Navigate to the **Solutions** tab, and select **Min_Max Markers**, under **Markers**.

17.3 Max peaks should then be added as markers on the data (see figure 23).

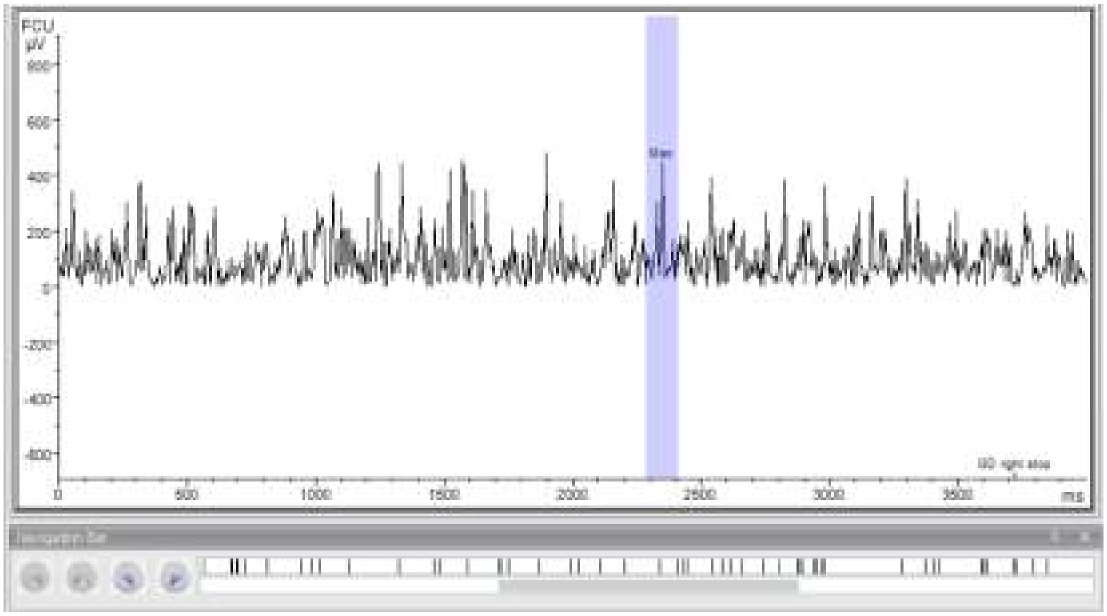

Fig. 23. **Max Marker** labelled in the MVC segment of the FCU muscle.

17.4 Go to **Export** and select **Peak Information**. Input **Max** into **Peak Name**, add the node **MinMax Markers** and select all participant's MVC data nodes. Rename the Output to "Max MVC" and check the **Export Mean Value Around Peak** box (see figure 24).

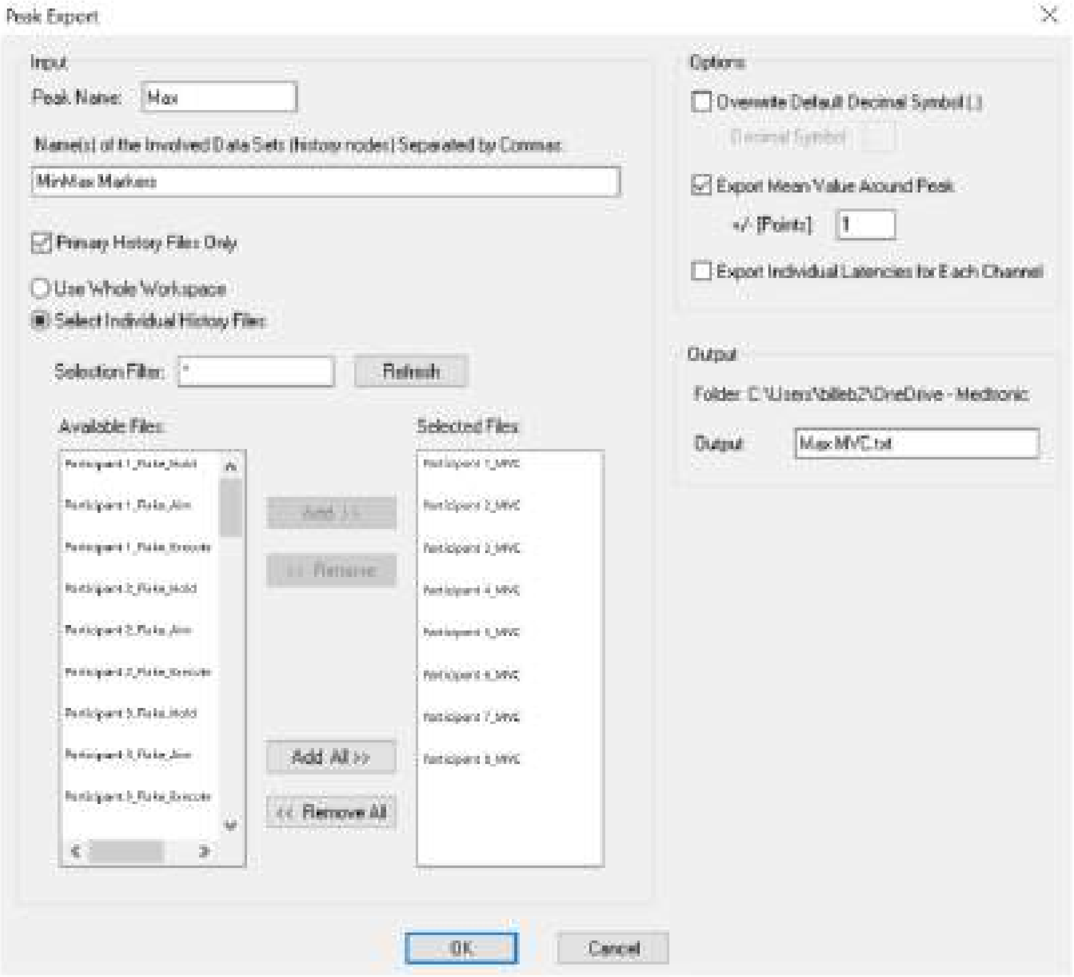

Fig. 24. **Peak Export** window to extract MVC values.

18 **Average**

Note

Averaging is used to extract the real signal from the noise in EEG. As both methodologies (EEG and sEMG) are recorded simultaneously, there are also ≥50 repetitions in the sEMG data. To extract a single data point for each muscle in each stage ("Hold", "Aim", and "Execute") all repetitions must be averaged.

18.1 Navigate to **Average** in **Segment Analysis Functions**, under the **Transformations** tab.

18.2 Select **Full Segment Range**. Averaging should be performed on all segments.

18.3 Repeat this process for the other markers of interest (i.e. all flake cutting stages, "Hold", "Aim", and "Execute").

19 **Exporting**

Note

Exportation is necessary to extract the EMG data for analysis in external software. Exported files can be produced in .txt, .dat, and .vhdr format. This protocol outlines the exportation of data as a .txt file. This enables the data to be imported into most statistical software programs, such as; **Microsoft Excel** [51], **PAST** [52], and **SPSS** [53].

19.1 To export the data as a .txt file, go to **Export** and select **Generic Data**.

19.2 Leave the **Base Name** in its default form "($h_$n)", and ensure the extension is .txt (see figure 25).

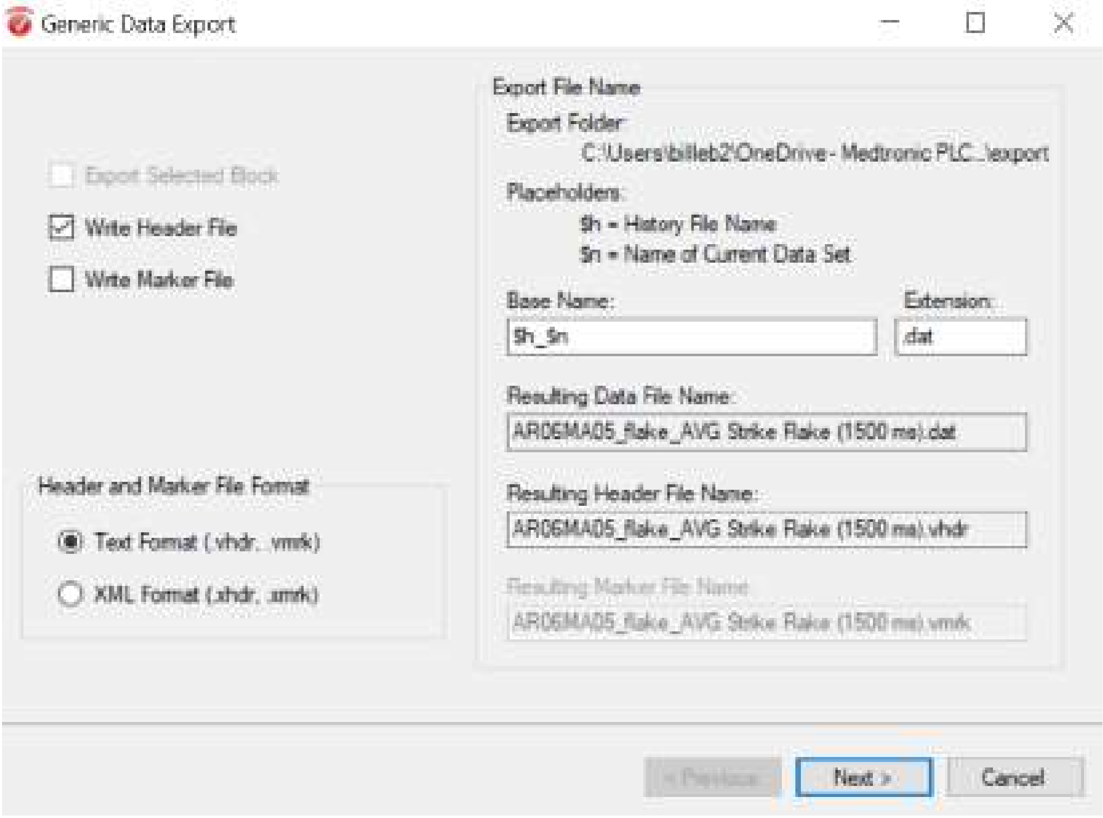

Fig. 25. **Generic Data Export** window default settings.

19.3 Select **Add Channels Names to the Data File** and select how values should be separated in the file, the data is separated with a space by default.

19.4 Once the data has been exported in a .txt format, you can import it into a spreadsheet program, such as **Microsoft Excel** [51] to create the %MVC values that will be used for data analysis and visualization. Open the .txt file in **Microsoft Excel**. In the **Text Import Wizard** window choose **Delimited** file type. Select the **Delimiter** that correctly separates your data (this was selected in Step 19.3). Finally, select **General** in **Column data format** (see figure 26).

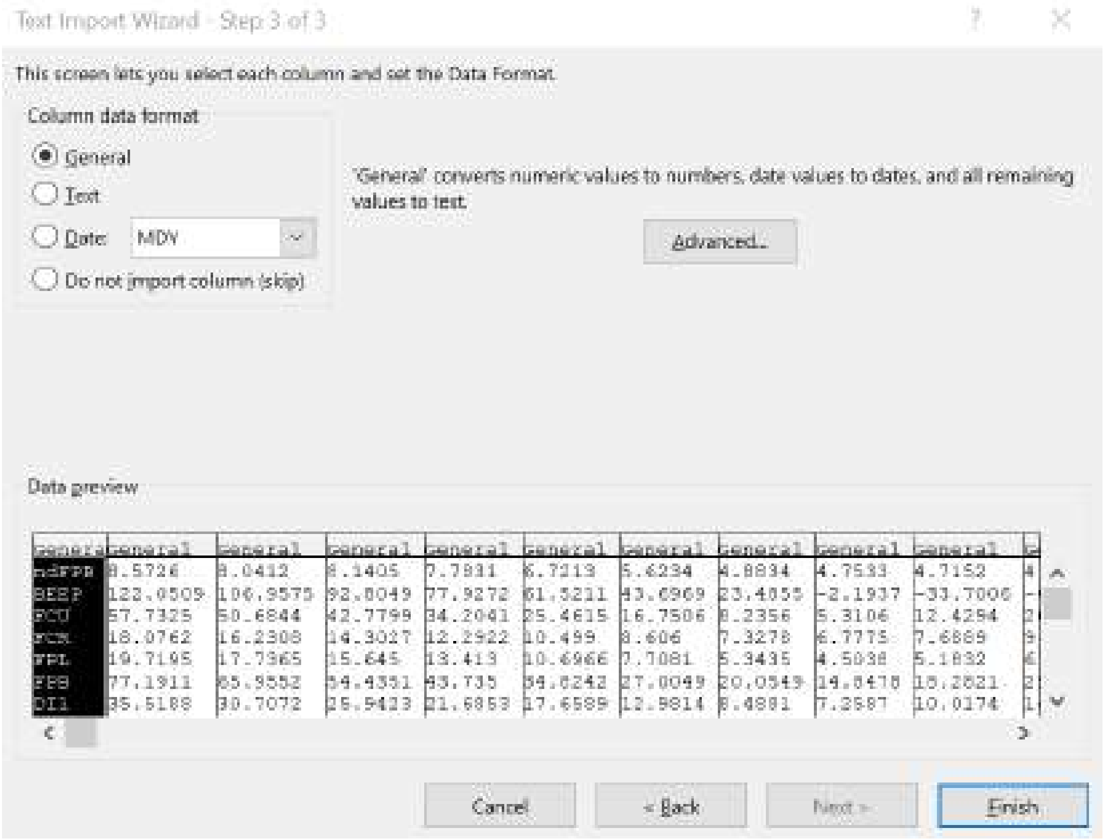

Fig. 26. **Import Text Wizard** window importing .txt file into an **Microsoft Excel** spreadsheet [51].

19.5 Once formatted, create an average from the 1000 ms data (0.4 ms per cell) by following this formula for each muscle (see figure 27):
=AVERAGE(B1:NTQ1)

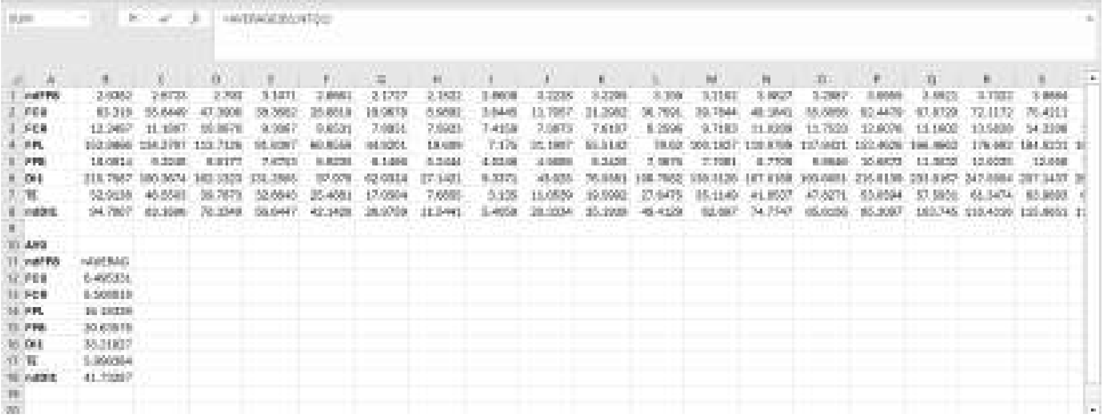

Fig. 27. **Microsoft Excel** spreadsheet [51] with stone tool cutting task ("Aim" stage) exported sEMG data.

19.6 Add the MVCs of each muscle to the worksheet to calculate the %MVC (see figure 28).

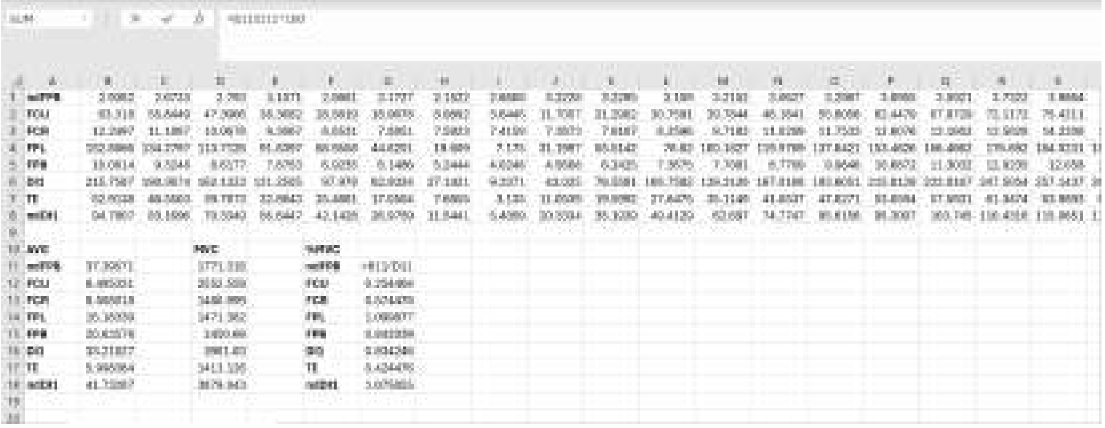

Fig. 28. **Microsoft Excel** spreadsheet [51] with stone tool cutting task ("Aim" stage) and MVC values for each muscle.

Note

%MVCs are the values used for all further analysis and statistical testing.

20 Calculating %MVCs

20.1 To create the %MVC value for each data point (every muscle in each stage of the task), follow this basic percentage creation formula:
(Average EMG data) ÷ (MVC value) × 100

20.2 Each value is now a %MVC value and can be compared intra and inter-individually. Values should not exceed 100%.

Note

In the rare instance that values during the task exceed the MVC values, replace the MVC value with this higher value, and recalculate the %MVC.

STEP CASE

surface Electromyography 12 steps

3.1 Locate all muscles/muscle groups to be monitored. Use an anatomical atlas (i.e. [9]) and Table 1 to determine the precise location of the muscles and ensure proper identification and correct placement of the sEMG **EasyCap Multitrode electrodes B18 (ref: B18-HSR-120)** on the skin's surface. Proper sensor placement is essential to ensure the detection of quality EMG signals [10,11].

A	B
**Muscles/Muscle Groups**	**Movement Action**
First dorsal *interosseous* (DI1)	Abducts and rotates the index finger, assists in adduction of the thumb.
Hypothenar eminence (HTE)	The muscle group contributes to the flexion, abduction, and lateral rotation of the fifth digit.
Flexor carpi radialis (FCR)	Flexes and radially abducts the wrist.
Flexor carpi ulnaris (FCU)	Adducts and flexes the wrist.
Flexor pollicis longus (FPL)	Flexes the thumb and assists in wrist flexion.
Thenar eminence (TE)	The muscle group contributes to the adduction, abduction, flexion, and medial rotation of the thumb.
Non-dominant first dorsal *interosseous* (ndDI1)	Abducts and rotates the index finger, assists in adduction of the thumb.
Non-dominant thenar eminence (ndTE)	The muscle group contributes to the adduction, abduction, flexion, and medial rotation of the thumb.

Table 1. The eight muscles/muscle groups monitored and their movement action. Adapted from Eteson et al. [12], following the anatomical placements based on Standring's [9] description.

Note

Muscles within the thenar and hypothenar eminences were grouped together due to the small surface area of the skin and their similar functionality, as surface electrodes are not able to accurately detect singular muscular activation. Crowding of electrodes can cause internal noise and artifacts for surrounding muscles [10].

Note

All muscles/muscle groups included in Table 1 are known for their importance during precision grasping and stone tool use [13–15]. Both the first and second digits in the non-dominant hand have also been shown to play an important role in stabilization during stone tool production and use [15–17], and therefore were also monitored as cutting tasks, like those described in this protocol, require stabilization of the target object (faux leather).

3.2 Instruct the participant to perform the relevant exercise of each muscle (see Table 1) to determine its exact location.

3.3 Once the location of each muscle has been established, ensure skin is free from dirt, excessive hair, and any creams or products that may affect the electrode signal or adherence to the skin's surface.

3.4 Prepare the surface of the skin by cleansing the area with **alcohol wipes N94842 (ref: 501 075, Winner Medical Co., LTD)**.

4 **Application of Electrolyte Gel**

4.1 Once prepped, apply two pea-sized amounts of **Abrasive Electrolyte-Gel (ref: 219-001-6-R, EasyCap Abralyt HiCl)** onto the skin directly along the fibers of the muscle belly, spatially separated (see figure 9).

Note

For each muscle recorded, two bipolar **EasyCap Multitrode electrodes B18 (ref: B18-HSR-120)** are attached to the skin's surface and connected to the **BrainVision LiveAmp wireless amplifier (ref: BP-200-3000)** via a **BrainVision BIP$_2$AUX Adapter (ref: label 001 11/2014)**. Bipolar electrodes have been found to reduce common noise and retain signals of interest ensuring a cleaner and more accurate signal of the muscles monitored [18].

Note

Ensure the **EasyCap Multitrode electrodes B18 (ref: B18-HSR-120)** and their **BrainVision BIP$_2$AUX Adapters (ref: label 001 11/2014)** correctly correspond to the AUX channels renamed in the **BrainVision Recorder software (version 1.24.0101, Brain Products GbmH, Gilching, Germany)** [1] (see figure 7).

4.2 Two **EasyCap Multitrode electrodes B18 (ref: B18-HSR-120)** are connected via each **BrainVision BIP$_2$AUX Adapters (ref: label 001 11/2014)**. Place one over the **Abrasive Electrolyte-Gel (ref: 219-001-6-R, EasyCap Abralyt HiCl)** on either side of the muscle and

gently push it down onto the skin. Once placed, secure the electrodes with **Kinesiotape TrueTape (True Tape Sports GmbH)** to enable the participant to move freely (see figure 10).

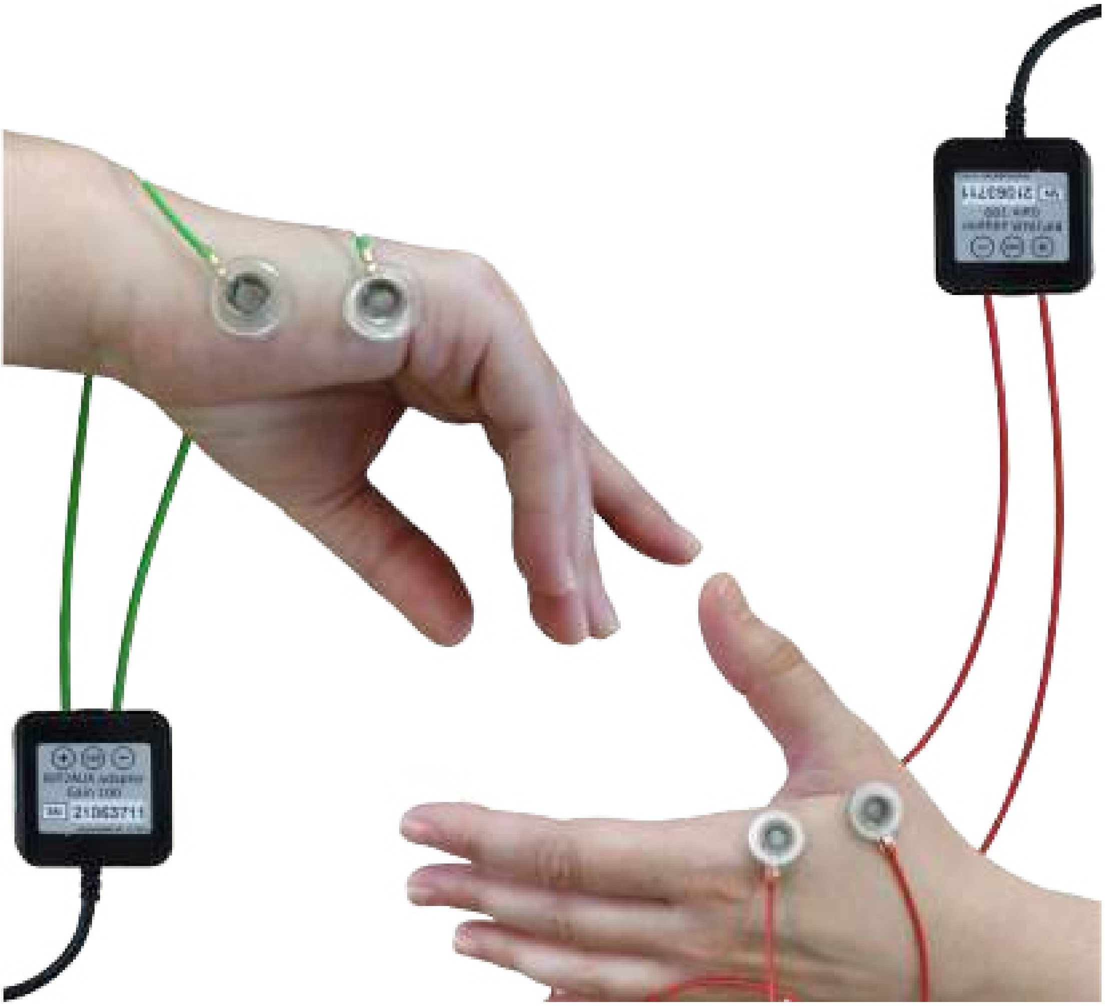

Fig. 9. Surface bipolar electrode application on the hypothenar eminence (green) and first dorsal interosseous (red).

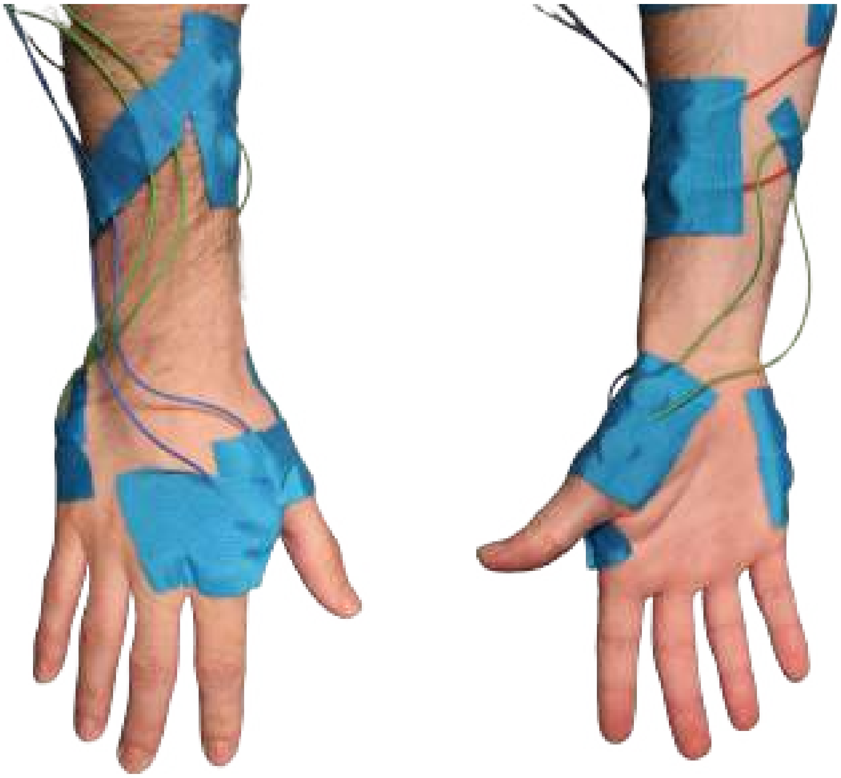

Fig. 10. **Kinesiotape TrueTape (True Tape Sports GmbH)** securing the electrodes to the skin's surface.

Note

Sweat can cause problems with electrode adherence on the skin’s surface. To avoid this, use **Kinesiotape TrueTape (True Tape Sports GmbH)** to secure electrodes onto the participant’s skin.

4.3 Repeat Steps 3.1 through 4.2, attaching all bipolar **EasyCap Multitrode electrodes B18 (ref: B18-HSR-120)** to the corresponding muscles.

4.4 Use the additional **EasyCap Multitrode electrode B18 (ref: B18-HSR-120)** in the center **GND** AUX of one **BrainVision BIP$_2$AUX Adapter (ref: label 001 11/2014)** to place a ground electrode. Place this electrode on connective tissue (i.e. a bony eminence or tendon [19]). The ground electrode is a common reference used to reduce interferences in sEMG applications [20].

Note

EMG signals are easily contaminated by external and internal noise, artifacts, and interference, which leads to loss of real muscle activation or inaccurate data recording [21]. These types of noise can be quelled through the use of bipolar and ground electrodes, proper placement, strict guidelines for participants during the experiment, and pre-processing of the data.

4.5 Ensure all **BrainVision BIP_2AUX Adapters (ref: label 001 11/2014)** are connected to the **BrainVision LiveAmp actiCAP Adapter (ref: BP-210-2100)**.

4.6 Ensure the **EasyCap Multitrode electrode B18 (ref: B18-HSR-120)**, including the wires attached to the **BrainVision BIP_2AUX Adapters (ref: label 001 11/2014)**, do not come in direct contact with external materials or surfaces such as clothing, table, chairs, or other body parts, etc. This is to avoid electrode contact being read as a true EMG signal.

Note

In addition to common external noise, electrodes placed close to the palm can cause interference, particularly during experiments with tool use, such as the stone tool tasks described in this protocol. Muscle placements have been decided meticulously to ensure this should not occur. Muscles on the palmer side of the hand and digits were not analyzed as direct electrode contact causes large external artifacts to be present in the recorded data. Alternatively, participants may sustain unnatural grasping patterns to avoid direct contact with the electrodes. However, it is particularly important to adhere to natural conditions as much as possible and record the natural grasping patterns acquired during the stone tools use, i.e. a pad-to-side precision grip during flake cutting, or a cradle five jaw power grip used during hammerstone pounding.

4.7 Check the signal strength of all muscles in the **BrainVision Recorder software (version 1.24.0101, Brain Products GbmH, Gilching, Germany)** [1] to ensure correct muscle placement and any potential external noise that can be eliminated before recording. To check muscle strength, repeat the exercises previously performed by the participant to ensure each sEMG signal is recording the expected amplitude. All muscles' amplitude varies, as the greater number of muscle fiber action potentials produce an approximated increase in sEMG amplitude [22]. However, if the signal amplitude is particularly low, or is not responding when the exercises for each of the muscles are performed, the placement may not be accurate. In this case, thoroughly re-do Steps 3.1 through 4.2 on the affected electrodes/muscles. See figure 11 for how varying raw muscular signals can look, as well as signal clipping (electrode contact or noise) and signal loss/deadline.

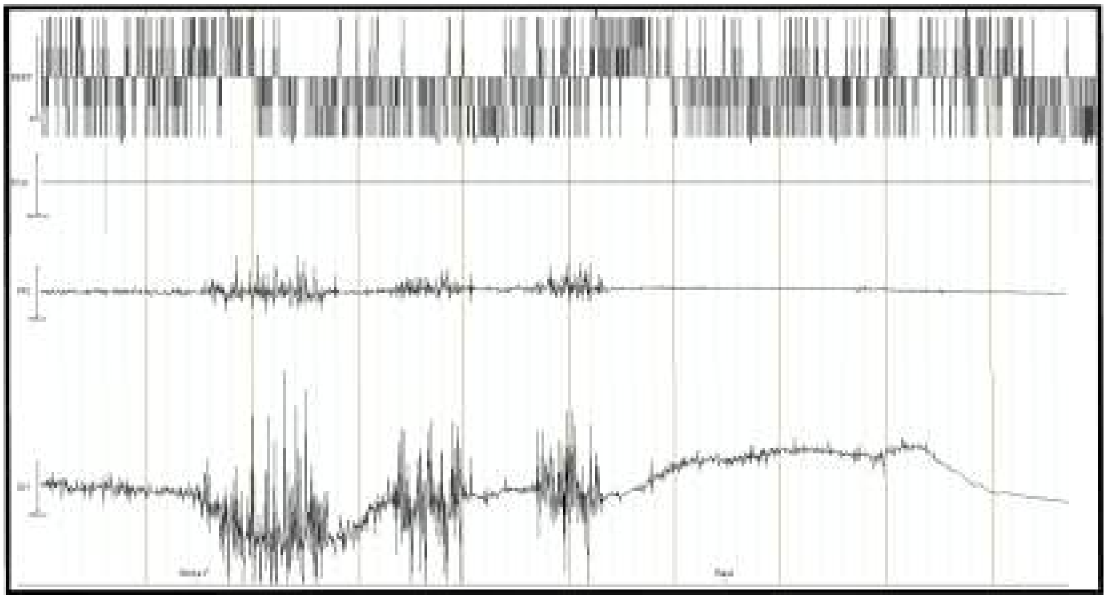

Fig. 11. Examples of various sEMG signals in **BrainVision Analyzer (version 2.2.1, Brain Products GbmH, Gilching, Germany)** [8]. Signal clipping (top "BEEP"); signal deadline (middle, "FCU"); regular sEMG signal of various muscles on the dominant hand (bottom, "FPL" and "DI1").

Protocol references

1. Brain Products. Brain Vision Recorder User Manual. 1.21.0402. Gilching: Brain Products GmbH; 2018
2. Aydin M, Carpenelli AL, Lucia S, Di Russo F. The Dominance of Anticipatory Prefrontal Activity in Uncued Sensory–Motor Tasks. Sensors (Basel). 2022 Aug 31;22(17).
3. Berchicci M, Lucci G, Pesce C, Spinelli D, Di Russo F. Prefrontal hyperactivity in older people during motor planning. Neuroimage. 2012 Sep;62(3):1750–60.
4. Berchicci M, Pontifex MB, Drollette ES, Pesce C, Hillman CH, Russo FD. From cognitive motor preparation to visual processing: The benefits of childhood fitness to brain health. Neurosci. 2015 Jul 9;298:211–9.
5. Affinito S, Eteson B, Tamayo Caceres L, Moos ET, Karakostis FA. Exploring the cognitive underpinnings of early hominin stone tool use through an experimental EEG approach. Sci Rep. 2024; (in press).
6. Reis PMR, Hebenstreit F, Gabsteiger F, von Tscharner V, Lochmann M. Methodological aspects of EEG and body dynamics measurements during motion. Front Hum Neurosci. 2014 Mar 24;8:1–19.
7. Ives J, Wigglesworth J. Sampling rate effect of surface EMG timing and amplitude measures. Clin Biomech Bristol Avon. 2003 Aug 1;18:543–52.
8. Brain Products. Brain Vision Analyzer User Manual. 2.2.1. Gilching: Brain Products GmbH; 2020
9. Standring S, editor. Gray's Anatomy: The Anatomical Basis of Clinical Practice. 42nd ed. Amsterdam: Elsevier; 2021.
10. Hermens HJ, Freriks B, Disselhorst-Klug C, Rau G. Development of recommendations for sEMG sensors and sensor placement procedures. J Electromyogr Kinesiol. 2000 Oct 1;10(5):361–74.
11. Stegeman D, Hermens H. Standards for surface electromyography: The European project Surface EMG for non-invasive assessment of muscles (SENIAM). Enschede: Roessingh Research and Development; 2007.
12. Eteson B, Affinito S, Moos ET, Karakostis FA. "How Handy was early hominin 'know-how'?" An experimental approach exploring efficient early stone tool use. Am J Biol Anthropol. 2024 Sep 2;185(3).
13. Key AJM, Farr I, Hunter R, Winter SL. Muscle recruitment and stone tool use ergonomics across three million years of Palaeolithic technological transitions. J Hum Evol. 2020 Jul 1;144.
14. Hamrick MW, Churchill SE, Schmitt D, Hylander WL. EMG of the human flexor pollicis longus muscle: implications for the evolution of hominid tool use. J Hum Evol. 1998 Feb;34(2):123–36.
15. Marzke MW, Toth N, Schick K, Reece S, Steinberg B, Hunt K, et al. EMG study of hand muscle recruitment during hard hammer percussion manufacture of Oldowan tools. Am J Phys Anthropol. 1998;105(3):315–32.
16. Marzke MW, Shackley MS. Hominid hand use in the pliocene and pleistocene: Evidence from experimental archaeology and comparative morphology. J Hum Evol. 1986 Sep 1;15(6):439–60.
17. Key AJM, Dunmore CJ. The evolution of the hominin thumb and the influence exerted by the non-dominant hand during stone tool production. J Hum Evol. 2015 Jan 1;78:60–9.
18. Mohr M, Schön T, von Tscharner V, Nigg BM. Intermuscular Coherence Between Surface EMG Signals Is Higher for Monopolar Compared to Bipolar Electrode Configurations. Front Physiol. 2018 May 17;9.
19. Islam MdJ, Ahmad S, Ferdousi A, Haque F, Reaz MBI, Bhuiyan MAS, et al. Optimizing electrode positions on forearm to increase SNR and myoelectric pattern recognition performance. Eng Appl Artif Intell. 2023 Jun 1;122.
20. Tankisi H, Burke D, Cui L, de Carvalho M, Kuwabara S, Nandedkar SD, et al. Standards of instrumentation of EMG. Clin Neurophysiol. 2020 Jan 1;131(1):243–58.
21. Boyer M, Bouyer L, Roy JS, Campeau-Lecours A. Reducing Noise, Artifacts and Interference in Single Channel EMG Signals: A Review. Sensors. 2023 Mar 8;23(6).
22. Enoka RM, Duchateau J. Inappropriate interpretation of surface EMG signals and muscle fiber characteristics impedes understanding of the control of neuromuscular function. J Appl Physiol. 2015 Dec 15;119(12):1516–8.

23. EEG caps in a nutshell [Internet]. EASYCAP GmbH; 2022 Nov [cited 2024 Mar 19]. Available from: https://www.easycap.de/wp-content/uploads/2018/02/EasyCap_CapHandling_Flyer_e.pdf

24. The 10-20 System for EEG [Internet]. TMSI Human Electrophysiology [cited 2024 Mar 25]. Available from: https://info.tmsi.com/blog/the-10-20-system-for-eeg

25. Leuchs L. Choosing your reference & why it matters [Internet]. Brain Products Press Release; 2019 [cited 2024 Mar 14]. Available from: https://pressrelease.brainproducts.com/referencing/

26. Stout D, Toth N, Schick K, Stout J, Hutchins G. Stone Tool-Making and Brain Activation: Position Emission Tomography (PET) Studies. J Archaeol Sci. 2000 Dec;27(12):1215–23.

27. Stout D, Chaminade T. The evolutionary neuroscience of tool making. Neuropsychologia. 2007;45(5):1091–100.

28. Renfrew C, Frith C, Malafouris L, Stout D, Toth N, Schick K, et al. Neural correlates of Early Stone Age toolmaking: technology, language and cognition in human evolution. Philos Trans R Soc B Biol Sci. 2008 Jun 12;363(1499):1939–49.

29. Stout D, Hecht E, Khreisheh N, Bradley B, Chaminade T. Cognitive Demands of Lower Paleolithic Toolmaking. PLOS ONE. 2015 Apr 15;10(4).

30. Hecht EE, Pargeter J, Khreisheh N, Stout D. Neuroplasticity enables bio-cultural feedback in Paleolithic stone-tool making. Sci Rep. 2023 Feb 18;13(1).

31. Hecht EE, Gutman DA, Khreisheh N, Taylor SV, Kilner J, Faisal AA, et al. Acquisition of Paleolithic toolmaking abilities involves structural remodeling to inferior frontoparietal regions. Brain Struct Funct. 2015 Jul 1;220(4):2315–31.

32. Putt SSJ, Wijeakumar S, Spencer JP. Prefrontal cortex activation supports the emergence of early stone age toolmaking skill. NeuroImage. 2019 Oct 1;199:57–69.

33. Luca J, Hazenfratz M, Monteith G, Sanchez A, Gaitero L, James F. Electrode scalp impedance differences between electroencephalography machines in healthy dogs. Can J Vet Res. 2021 Oct;85(4):309–11.

34. Górecka J, Makiewicz P. The Dependence of Electrode Impedance on the Number of Performed EEG Examinations. Sensors. 2019 Jun 8;19(11).

35. Greischar LL, Burghy CA, van Reekum CM, Jackson DC, Pizzagalli DA, Mueller C, et al. Effects of electrode density and electrolyte spreading in dense array electroencephalographic recording. Clin Neurophysiol Off J Int Fed Clin Neurophysiol. 2004 Mar;115(3):710–20.

36. Tenke CE, Kayser J. A convenient method for detecting electrolyte bridges in multichannel electroencephalogram and event-related potential recordings. Clin Neurophysiol. 2001 Mar;112(3):545–50.

37. Cronin NJ, Kumpulainen S, Joutjärvi T, Finni T, Piitulainen H. Spatial variability of muscle activity during human walking: The effects of different EMG normalization approaches. Neuroscience. 2015 Aug 6;300:19–28.

38. Boudewyn MA, Luck SJ, Farrens JL, Kappenman ES. How Many Trials Does It Take to Get a Significant ERP Effect? It Depends. Psychophysiology. 2017 Dec 20;55(6).

39. Baum F, Wolfensteller U, Ruge H. Learning-Related Brain-Electrical Activity Dynamics Associated with the Subsequent Impact of Learnt Action-Outcome Associations. Front Hum Neurosci. 2017 May 15;11.

40. Amin HU, Ullah R, Reza MF, Malik AS. Single-trial extraction of event-related potentials (ERPs) and classification of visual stimuli by ensemble use of discrete wavelet transform with Huffman coding and machine learning techniques. J NeuroEngineering Rehabil. 2023 Jun 2;20.

41. Riehle A, Vaadia E, editors. Motor Cortex in Voluntary Movements: A Distributed System for Distributed Functions. 1st ed. Boca Raton: CRC Press; 2004.

42. Gage K. Signal Quality Monitor – EMGworks [Internet]. Delsys Europe. [cited 2024 Apr 2]. Available from: https://delsyseurope.com/emgworks/signal-quality-monitor/

43. De Luca CJ, Donald Gilmore L, Kuznetsov M, Roy SH. Filtering the surface EMG signal: Movement artifact and baseline noise contamination. J Biomech. 2010 May;43(8):1573–9.
44. Potvin JR, Brown SHM. Less is more: high pass filtering, to remove up to 99% of the surface EMG signal power, improves EMG-based biceps brachii muscle force estimates. J Electromyogr Kinesiol. 2004 Jun 1;14(3):389–99.
45. Merletti R. Standards for Reporting EMG Data (1999). J Electromyogr Kinesiol. 2018 Oct; 42.
46. Kappenman ES, Luck SJ. The Effects of Electrode Impedance on Data Quality and Statistical Significance in ERP Recordings. Psychophysiol. 2010 Sep 1;47(5):888–904.
47. Kappenman ES, Luck SJ. The Effects of Electrode Impedance on Data Quality and Statistical Significance in ERP Recordings. Psychophysiology. 2010 Sep 1;47(5):888–904.
48. Rose W. Raw signal amplification – Electromyogram Analysis [Internet]. Mathematics and Signal Processing for Biomechanics; 2019 Oct [cited 2022 Apr 18] Available from: https://www.udel.edu/biology/rosewc/kaap686/notes/EMG-analysis.pdf
49. Negro F, Keenan K, Farina D. Power spectrum of the rectified EMG: when and why is rectification beneficial for identifying neural connectivity? J Neural Eng. 2015 Jun;12(3).
50. Neto OP, Christou EA. Rectification of the EMG signal impairs the identification of oscillatory input to the muscle. J Neurophysiol. 2010 Feb;103(2):1093–103.
51. Excel help & learning [Internet] Microsoft Support. [cited 2024 Apr 5]. Available from: https://support.microsoft.com/en-gb/excel
52. Hammer, Ø., Harper, D. A. T., & Ryan, P. D. Past: Paleontological statistics software package for education and data analysis. Palaeontol Electronica. 2001;4:1–9.
53. Field A. Discovering Statistics using IBM SPSS Statistics. 4th ed. London: SAGE Publications. 2013.
54. Resampling [Internet]. ILCB Center of Experimental Resources. [cited 2024 Apr 2]. Available from: https://blricrex.hypotheses.org/ressources/eeg/pre-processing-for-erps/resampling
55. Gonçales LJ, Farias K, Kupssinskü L, Segalotto M. The effects of applying filters on EEG signals for classifying developers' code comprehension. J Appl Res Technol. 2021 Dec 31;19(6):584–602.
56. Yao D, Qin Y, Hu S, Dong L, Bringas Vega ML, Valdés Sosa PA. Which Reference Should We Use for EEG and ERP practice? Brain Topogr. 2019 Apr 29;32:530–49.
57. Yao D. A method to standardize a reference of scalp EEG recordings to a point at infinity. Physiol Meas. 2001 Oct 1;22(4):693–711.
58. Dong L, Li F, Liu Q, Wen X, Lai Y, Xu P, et al. MATLAB Toolboxes for Reference Electrode Standardization Technique (REST) of Scalp EEG. Front Neurosci. 2017 Oct 30;11.
59. Villasana FC. Getting to know EEG artifacts and how to handle them in BrainVision Analyzer. 2022 [cited 2024 Apr 2]. In: Brain Products Press Release [Internet]. Gilching: Brain Products. Available from: https://pressrelease.brainproducts.com/eeg-artifacts-handling-in-analyzer/
60. Luck S. Hints for ICA-based artifact correction. 2018 Jun 24 [cited 2024 Apr 2]. In: ERP Methodology Blog [Internet]. ERP Works. Available from: https://erpinfo.org/blog/2018/6/18/hints-for-using-ica-for artifact-correction
61. Plank M. Ocular Correction ICA. 2013 Dec 1 [cited 2024 Apr 2]. In: Support & Tips, ICA - Independent Component Analysis, Transformations [Internet]. Brain Products. Available from: https://www.brainproducts.com/support-resources/ocular-correction-ica/
62. Abo-Zeid MAZ, Ahmed SM, Abbas SN. A New EEG Acquisition Protocol for Biometric Identification Using Eye Blinking Signals. Int J Intell Syst Appl. 2015 May 1; 7(6):48–54.
63. Subramaniyam NP. Getting Rid of Eye Blink in the EEG. 2018 Jun 11. [cited 2024 Apr 2]. In: Lab Talk [Internet]. Sapien Labs. Available from: https://sapienlabs.org/getting-rid-of-eye-blink-in-the-eeg/

64. Baseline Correction. [cited 2024 Apr 9]. In: EEG Data Processing [Internet]. Bern: Cognitive Computational Neuroscience. Available from: https://neuro.inf.unibe.ch/AlgorithmsNeuroscience/YOUR%20URL/AlgorithmsNeuroscience/Tutorial_fils/BaselineCorrection.html
65. Liland KH, Rukke EO, Olsen EF, Isaksson T. Customized baseline correction. Chemom Intell Lab Syst. 2011 Nov 15;109(1):51–6.
66. Baseline correction of neural signals. [cited 2024 Apr 4]. In: Tutorials [Internet]. Toronto: Auditory Aging. Available from: https://www.auditoryaging.com/tutorial-baselinecorrection
67. Jiang X, Bian GB, Tian Z. Removal of Artifacts from EEG Signals: A Review. Sensors. 2019 Feb 26;19(5).
68. Graham R. Exploring the minimum number of trials needed to accurately detect concealed information using EEG. The Plymouth Student Scientist. 2021 Dec 24; 42(2):532–47.
69. Al-Fahoum AS, Al-Fraihat AA. Methods of EEG Signal Features Extraction Using Linear Analysis in Frequency and Time-Frequency Domains. Int Sch Res Notices. 2014 Feb 13.
70. Spectral Analysis using FFT 2018 Mar. [cited 2024 Jul 2]. In: BrainVision Analyzer 2 Webinar [Internet]. Gilching: Brain Products. Available from: https://www.brainproducts.com/files/webinar/2018_WB_3_FFT.pdf
71. Vallat R. Bandpower of an EEG signal. 2018 May. [cited 2024 Apr 24]. [Internet]. Available from: https://raphaelvallat.com/bandpower.html
72. Dempster J. Signal Analysis and Measurement. In: Dempster J, editor. The Laboratory Computer (Biological Techniques Series). London: Academic Press; 2001. p. 136–71.
73. Young CS. The Compromise of Electromagnetic Signals. In: Young CS, editor. Information Security Science. Oxford: Syngress; 2016. p. 159–84.
74. Rugg MD. Event-related/Evoked Potentials. In: Smelser NJ, Baltes PB, editors. International Encyclopedia of the Social & Behavioral Sciences. Oxford: Pergamon; 2001. p. 4962–6.
75. Borgognone MG, Bussi J, Hough G. Principal component analysis in sensory analysis: covariance or correlation matrix? Food Qual Prefer. 2001 Jul 1;12(5):323–6.
76. Jackson DA. Stopping Rules in Principal Components Analysis: A Comparison of Heuristical and Statistical Approaches. Ecol. 1993 Dec 1;74(8):2204–14.
77. Kirstein C. Sleeping and Dreaming. In: Enna SJ, Bylund DB, editors. xPharm: The Comprehensive Pharmacology Reference. New York: Elsevier; 2008. p. 1–4. Available from: https://www.sciencedirect.com/science/article/pii/B9780080552323603198
78. Pfurtscheller G, Lopes da Silva FH. Event-related EEG/MEG synchronization and desynchronization: basic principles. Clin Neurophysiol. 1999 Nov;110(11):1842–57.
79. Nakayashiki K, Saeki M, Takata Y, Hayashi Y, Kondo T. Modulation of event-related desynchronization during kinematic and kinetic hand movements. J Neuroeng Rehabil. 2014 May 30;11.
80. Jasper H, Penfield W. Electrocorticograms in man: Effect of voluntary movement upon the electrical activity of the precentral gyrus. Arch Psychiatr Nervenkr. 1949 Jan;183:163–74.
81. Tzagarakis C, Ince NF, Leuthold AC, Pellizzer G. Beta-Band Activity during Motor Planning Reflects Response Uncertainty. J Neurosci. 2010 Aug 25;30(34):11270–7.
82. Pfurtscheller G. Functional brain imaging based on ERD/ERS. Vision Res. 2001 May 1;41(10):1257–60.
83. Formaggio E, Storti SF, Boscolo Galazzo I, Gandolfi M, Geroin C, Smania N, et al. Modulation of event-related desynchronization in robot-assisted hand performance: brain oscillatory changes in active, passive and imagined movements. J Neuroeng Rehabil. 2013 Feb 26;10.

84. Plummer TW, Oliver JS, Finestone EM, Ditchfield PW, Bishop LC, Blumenthal SA, et al. Expanded geographic distribution and dietary strategies of the earliest Oldowan hominins and Paranthropus. Science. 2023 Feb 10;379(6632):561-6.
85. Bril B, Parry R, Dietrich G. How similar are nut-cracking and stone-flaking? A functional approach to percussive technology. Philos Trans R Soc B Biol Sci. 2015 Nov 19;370(1682).
86. Toth N, Schick K. The Oldowan: The Tool Making of Early Hominins and Chimpanzees Compared. Annu Rev Anthropol. 2009 Oct 21;38(2009):289–305.
87. Susman RL. Who Made the Oldowan Tools? Fossil Evidence for Tool Behavior in Plio-Pleistocene Hominids. J Anthropol Res. 1991 Jul;47(2):129–51.
88. Newson JJ, Thiagarajan TC. EEG Frequency Bands in Psychiatric Disorders: A Review of Resting State Studies. Front Hum Neurosci. 2019 Jan 9;12:521.
89. Nayak CS, Anilkumar AC. EEG Normal Waveforms. In: StatPearls [Internet]. Treasure Island (FL): StatPearls Publishing; 2024. Available from: http://www.ncbi.nlm.nih.gov/books/NBK539805/
90. Banoczi W. How some drugs affect the electroencephalogram (EEG). Am J Electroneurodiagnostic Technol. 2005 Jun 1;45(2):118–29.
91. Fisher DJ, Daniels R, Jaworska N, Knobelsdorf A, Knott VJ. Effects of acute nicotine administration on behavioral and neural (EEG) correlates of working memory in non-smokers. Brain Res. 2012 Jan 6;1429:72–81.
92. Tcheslavski GV. Effects of tobacco smoking and schizotypal personality on spectral contents of spontaneous EEG. Int J Psychophysiol. 2008 Oct;70(1):88–93.
93. Gilbert DG, Dibb WD, Plath LC, Hiyane SG. Effects of nicotine and caffeine, separately and in combination, on EEG topography, mood, heart rate, cortisol, and vigilance. Psychophysiology. 2000 Sep;37(5):583–95.
94. Gladilin VN, Sitliviy VI. On the Pre-Oldowan Development Stage of the Society: To the Memory of Glynn Isaac — Outstanding Investigator in African Prehistory. Anthropol 1962-. 1987;25(3):193–204.
95. Hayden B. What Were They Doing in the Oldowan? an Ethnoarchaeological Perspective on the Origins of Human Behavior. Lithic Technol. 2008 Sep 1;33(2):105–39.
96. Titton S, Barsky D, Bargallo A, Vergès JM, Guardiola M, Solano JG, et al. Active percussion tools from the Oldowan site of Barranco León (Orce, Andalusia, Spain): The fundamental role of pounding activities in hominin lifeways. J Archaeol Sci. 2018 Aug 1;96:131–47.
97. Rogers C. Design Made Easy with Inkscape: A practical guide to your journey from beginner to pro-level vector illustration. Birmingham: Packt Publishing; 2023.

Acknowledgements

This research was supported by the German Research Foundation (DFG FOR 2237: Words, Bones, Genes, Tools – Tracking Linguistic, Cultural and Biological Trajectories of the Human Past; PIs; Katerina Harvati and Gerhard Jäger). We are very grateful to Elena Theresa Moos for their expertise and production of all stone tools used in the study, and to Lourdes Tamayo Cáceres for their invaluable technical support. Additionally, we are thankful to the Max Planck Institute for Intelligent Systems (Prof. B. Schölkopf and B. Battes) for kindly granting us access to their facilities, software, and equipment. Finally, thanks are due to all the participants who volunteered to participate in this study (from the University of Tübingen).
