# Supplementary material for: The mind & muscles: Introducing a validated EEG/EMG protocol for recording cognitive-muscular interactions in experimental archaeology
Source: PLoS One. 2025 May 23;20(5):e0324103. doi: 10.1371/journal.pone.0324103 (PMC12101640; doi:10.1371/journal.pone.0324103)
Supplement: S2 Table — Muscle abbreviations are as follows: FCR, flexor carpi radialis; FCU, flexor carpi ulnaris; FPL, flexor pollicis longus; TE, thenar eminence (consisting of abductor pollicis brevis, flexor pollicis brevis, and opponens pollicis); DI1, first dorsal interosseus; HTE, hypothenar eminence (consisting of abductor digiti minimi, flexor digiti minimi, and opponens digit minimi). (PDF) [file pone.0324103.s002.pdf]

				Factor loadings					
**Task**	**Principal Component**	**Eigenvalue**	**% of variance**	**DI1**	**HTE**	**FCR**	**FCU**	**FPL**	**TE**
**Flake-Cutting**	PC 1	4.22	70.27	0.43	0.37	0.42	0.43	0.43	0.35
	PC 2	0.70	11.59	-0.19	0.43	-0.10	-0.25	-0.44	0.72
