# Supplementary figures and images for: The mind & muscles: Introducing a validated EEG/EMG protocol for recording cognitive-muscular interactions in experimental archaeology
Source: PLoS One. 2025 May 23;20(5):e0324103. doi: 10.1371/journal.pone.0324103 (PMC12101640; doi:10.1371/journal.pone.0324103)

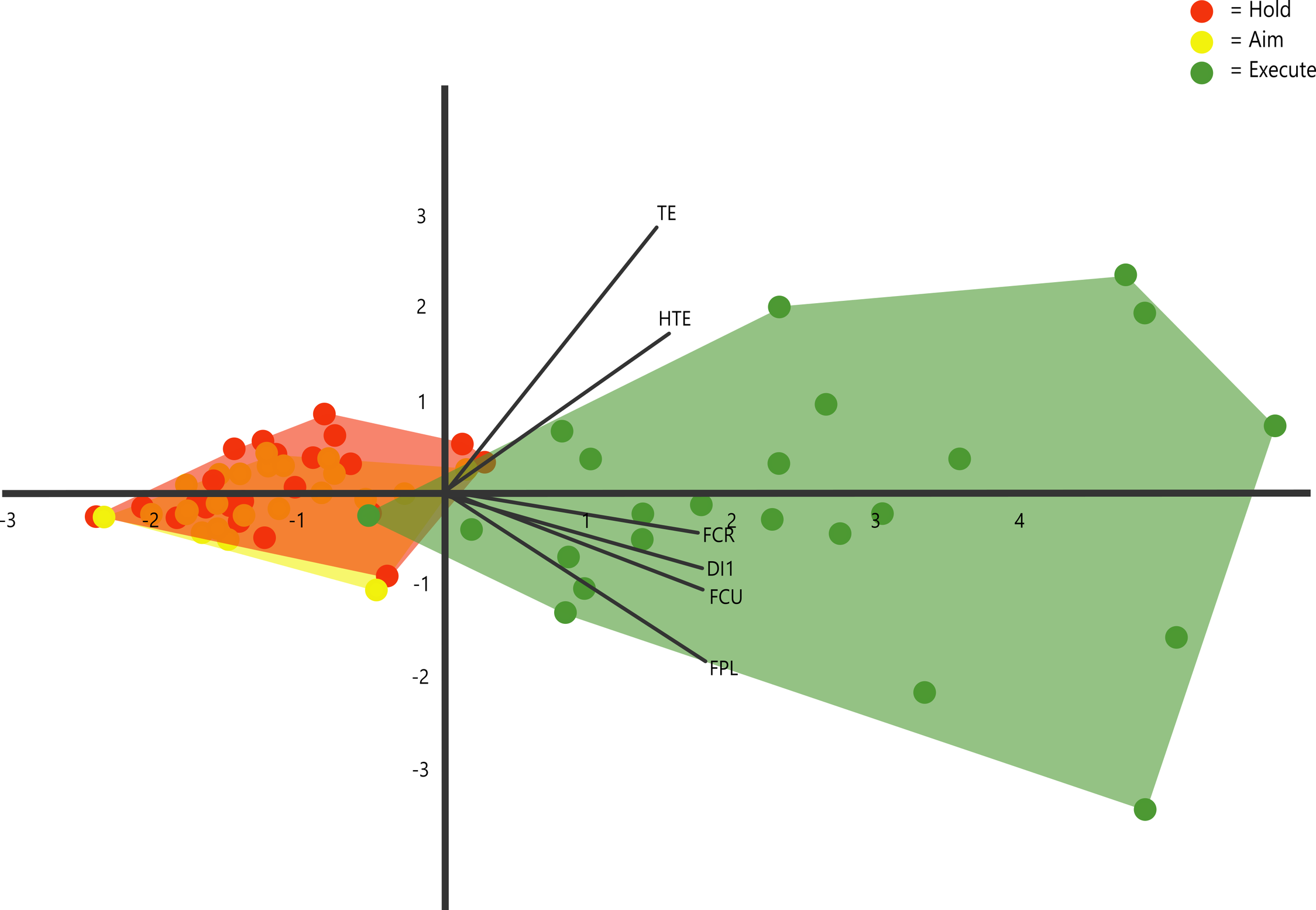

Supplement: S3 Fig — Phases are color-labeled (Red = Hold; Yellow = Aim; Green = Execute). PC 1 (70.27% of variance) maintains a clear distinction between Execute and the other phases of the task (Hold and Aim). The Execute phase displays increased muscular activation across all dominant hand muscles (factor loadings are listed in S2 Table). (TIF) [file pone.0324103.s003.tif]
